# New multicomponent reactions in water: a facile synthesis of 1,3-dioxo-2-indanilidene-heterocyclic scaffolds and indenoquinoxalines through reaction of ninhydrin-malononitrile adduct with diverse *N*-binucleophiles†

**DOI:** 10.1039/d2ra06469c

**Published:** 2022-11-24

**Authors:** Zahra Rahimi, Mohammad Bayat, Hajar Hosseini

**Affiliations:** Department of Chemistry, Faculty of Science, Imam Khomeini International University Qazvin Iran bayat_mo@yahoo.com m.bayat@sci.ikiu.ac.ir

## Abstract

We report here a highly efficient green approach for the synthesis of imidazolidin-2-ylidene-indenedione, pyrimidine-2-ylidene-indenedione and indenoquinoxaline derivatives through the one-pot three-component reaction between ninhydrin, malononitrile and various diamines in water medium under catalyst-free conditions. High yields (73–98%) of the target products were achieved with short reaction times at room temperature. Simple workup, no column chromatography, good to excellent yields, rapid reaction and green solvent are the prominent advantages of this protocol.

## Abstract 2

A one-pot three-component synthesis of indenoquinoxalines and various exocyclic enaminones, imidazolidin-2-ylidene-indenedione, pyrimidine-2-ylidene-indenedione, in water.

## Graphical abstract

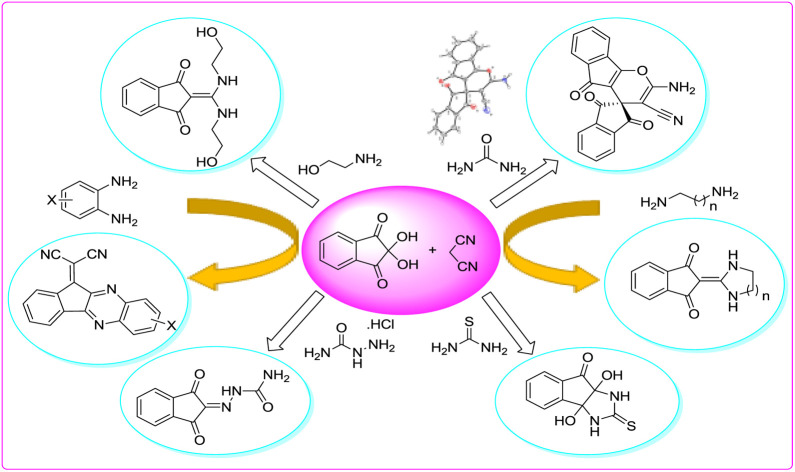

## Introduction

Multicomponent reactions (MCRs), which involve at least three starting substrates, have become a useful tool for the synthesis of important chemical and biological compounds. These reactions have provided environmentally benign, operationally simple and economically viable protocols to give products in high yields and minimum waste generation.^1–4^

In the past two decades, the principles of green chemistry have influenced organic synthesis. Based on this, the synthesis processes including organic compounds have been considered on waste prevention, safer solvents, design for high energy efficiency, creation of atom economy and use of renewable raw materials.^5^ Minimizing the amount of reactants (*e.g.*, starting materials, solvents, catalysts, and auxiliaries) which do not become part of the desired product, is the first and most important principle of green chemistry. Accordingly, the solvents used for chemical reactions and in the separation and purification steps are the main sources of waste.^6^

It has become clear that chemical and related industries, such as pharmaceuticals, are facing serious environmental and health concerns. Many classical synthetic methods with a wide range of applications, generate a large amount of waste.^7^

Thus, there is a need for developing facile, efficient, and non-polluting synthetic procedures that reduce or eliminate the use of organic solvents and hazardous substances. Nowadays, searching for a reaction media to replace volatile, flammable and toxic solvents that are usually employed in organic synthesis, has become a priority for the expansion of green chemical processes.^8,9^ This “green” approach includes the identification of alternative chemical reactions to achieve environmental and economic advantages.^10,11^ Therefore the synthetic value of MCRs will definitely increase when they are carried out in an aqueous medium.

From both economic and environmental points of view, water has appeared as the medium of choice for carrying out the organic reactions, because it is the safest, most environmentally acceptable and most abundant solvent.^12,13^ In addition, water generally facilitates the work-up procedures, because most organic compounds are easily separated from aqueous media due to their lipophilicity. Also, many organic reactions that occur in water, exhibit significant rate enhancements.^14^ Finally, water can create unique reactivity and selectivity with new solvation processes.^15^ Considering these advantages, efforts to design one-pot multicomponent reactions in aqueous media for the synthesis of diverse and functionalized heterocyclic scaffolds have become an attractive research area.

The prevalence of imidazolidine family in pharmaceutical chemistry journals and patents has increased significantly in recent years. There is a variety of imidazolidine containing compounds that have been reported as CCR1 antagonists with anti-inflammatory effects,^16^ and as sodium channel inhibitor that it is commonly applied to treat cardiac arrhythmia.^17^ A short set of compounds using an imidazolidin-4-one scaffold were synthesized and evaluated for inhibitory activity in human leukocytes elastase for treatment of emphysema.^18^

In addition imidazolidine derivatives form a group of very useful organic catalysts generally referred to as iminium catalysts. They have a well understood mode of activation that can be used in a variety of different stereoselective reactions.^19^

**Fig. 1:**
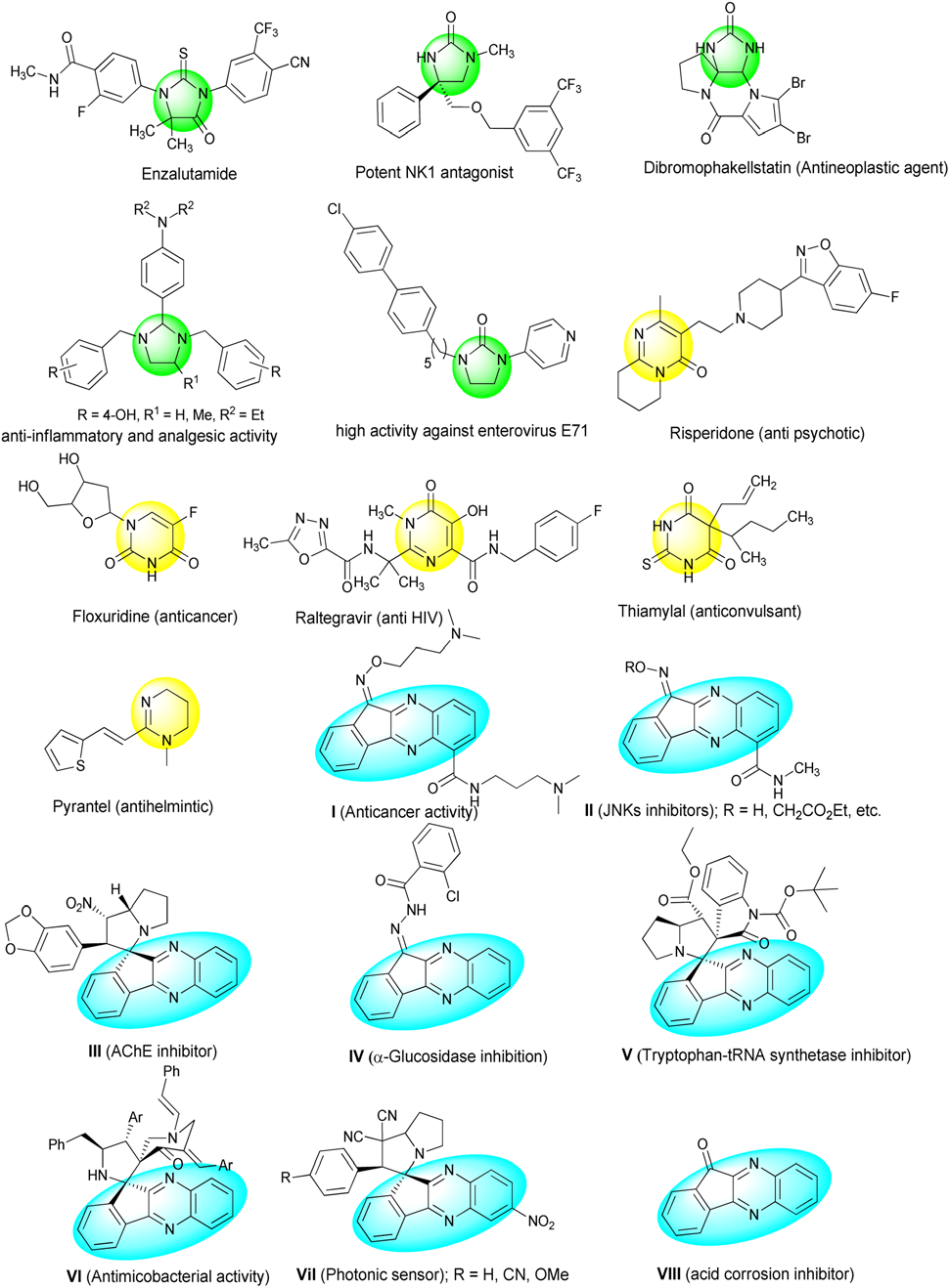
Selected bioactive imidazoidine, pyrimidine and indeno[1,2-*b*]quinoxaline derivatives.

In medicinal chemistry pyrimidine nucleus is also well known for its remarkable therapeutic applications including anticancer,^20^ antiviral,^21^ antimicrobial,^22^ anti-inflammatory,^23^ analgesic,^24^ antioxidant^25^ and antimalaria^26,27^ activities. Some biologically active drugs with imidazolidine and pyrimidine skeletons are shown in Fig. 1.^28–30^

Indeno[1,2-*b*]quinoxaline skeleton exist in a large number of drug candidates (Fig. 1).^31^ They exhibit various biological properties, such as antitumor activity (I),^32^ c-Jun N-terminal kinase (JNK) inhibition (II),^33^ acetylcholinesterase (AChE) inhibitory activity (III),^34^ α-glucosidase inhibition (IV),^35^ tryptophan-tRNA synthetase (TrpRS) inhibition (V)^36^ and antimicobacterial activity (VI).^37^ In addition, substituted spiro indeno[1,2-*b*]quinoxaline (VII) can be utilized as a photonic sensor to detect fluorescent dyes in the waste effluents of textiles, dyes, paper, and other industrial products^38^ and 11*H*-indeno[1,2-*b*]quinoxalin-11-one (VIII) shows acid corrosion inhibitory effect on mild steel surfaces.^39^ (Fig. 1).

Obviously, the synthesis of new classes of these nuclei may give a library of structures as possible candidates for various biological activities. Herein, we describe an efficient synthesis of novel imidazolidine and pyrimidine scaffolds having an exocyclic double bond linked to 1,3-indendione. In addition, the synthesis of 2-(11*H*-indeno[1,2-*b*]quinoxalin-11-ylidene)malononitriles are reported. All these reactions were carried out in a one-pot operation in water at room temperature.

## Results and discussion

**Scheme 1:**
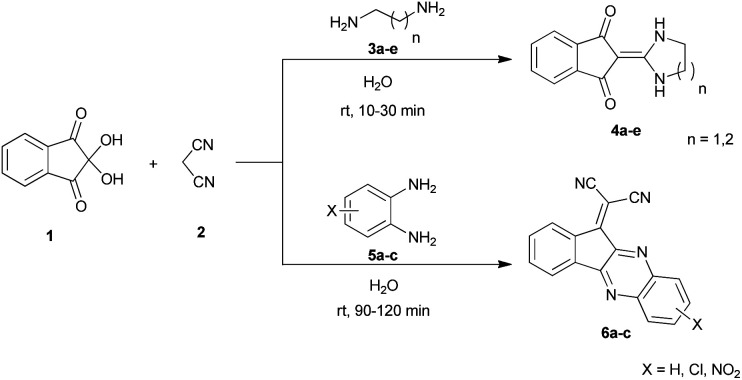
Synthetic scheme for the generation of products 4, 6.

As a result of our continuing efforts on one-pot processes, we want to report a one-pot three-component reaction of ninhydrin 1, malononitrile 2 and various diamines 3 in water. These reactions led to the synthesis of exocyclic enaminones including imidazolidine-2-ylidene-indenedione and pyrimidine-2-ylidene-indenedione derivatives 4 as well as indenoquinoxaline products 6 (Scheme 1). To the best of our knowledge, the use of a one-pot reaction to construct these products has not been previously reported.^40,41^

### Optimization of the conditions

Initially, ninhydrin 1, malononitrile 2 and ethylenediamine 3a were used as model substrates to optimize the reaction conditions (Table 1). The experimental results showed when water was used as solvent without any catalyst at room temperature, the reaction was completed in 10 minutes and the yield of product, 2-(imidazolidin-2-ylidene)-1*H*-indene-1,3(2*H*)-dione 4a, was 98% (Table 1, entry 1). We also studied the effect of other solvents. As can be seen in Table 1, the reaction in ethanol and acetonitrile also led to the desired product (entry 2 and 3). In these cases, the time to complete the reaction was longer and the efficiency was lower compared to water. With dichloromethane and chloroform the desired products were not formed at all (entry 4 and 5).

**Table 1:** Optimization conditions for the formation of 4a^a^

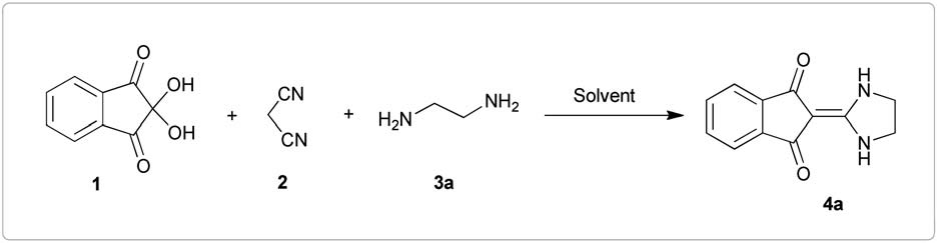				
Entry	Solvent	Time (min)	Temp (°C)	Yield (%)
**1**	**H**_**2**_ **O**	**10**	**rt**	**98**
2	EtOH	30	rt	82
3	CH_3_CN	30	rt	75
4	CH_2_Cl_2_	120	rt	No reaction
5	CHCl_3_	120	rt	No reaction

a Reagents and conditions: ninhydrin (1 mmol), malononitrile (1 mmol), diamine (1 mmol), solvent (10 ml).

With information obtained from optimization conditions table, we could synthesize target compounds 2-(imidazolidin-2-ylidene)-1*H*-indene-1,3(2*H*)-diones, 2-(tetrahydropyrimidin-2(1*H*)-ylidene)-1*H*-indene-1,3(2*H*)-diones 4a–e and 2-(11*H*-indeno[1,2-*b*]quinoxalin-11-ylidene)malononitriles 6a–c in good to high yields (73–98%) using ninhydrin 1, malononitrile 2, linear diamines 3a–e or aryl diamines 5a–c as starting materials (Scheme 1).

The reactions were completed after 10–30 min to afford corresponding heterocyclic enaminones 4a–e. For the synthesis of indenoquinoxalines 6a–c, the reaction time was 90–120 min. The results are summarized in Table 2.

**Table 2:** Compounds 4a–e and 6a–c^a^

Entry	Diamine	Product	Time (min)	Yield (%)	Mp (°C)
1	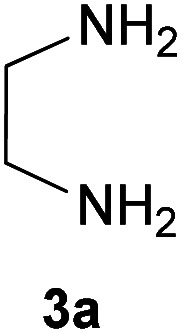	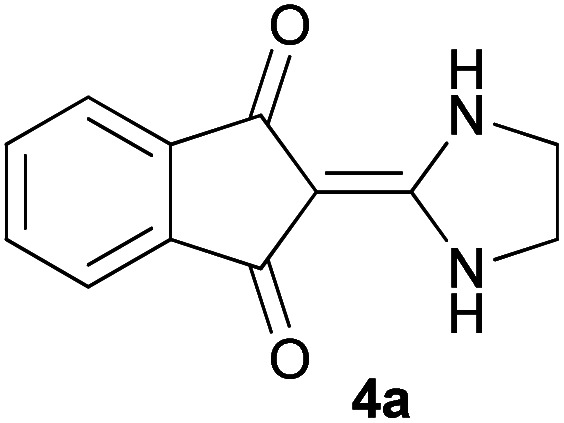	10	98	227–229
2	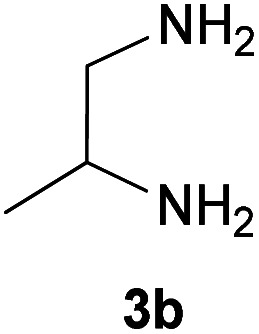	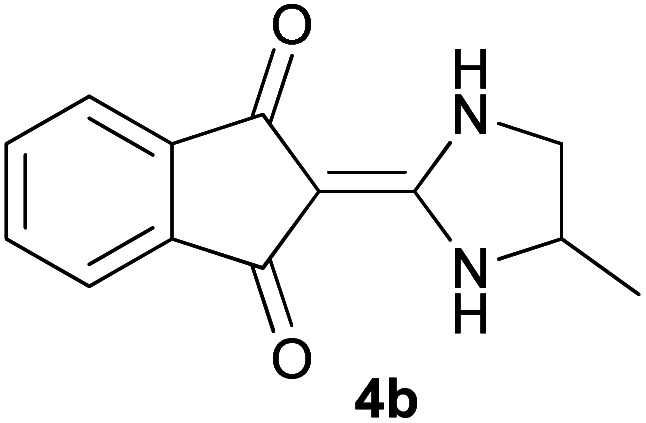	25	76	240–242
3	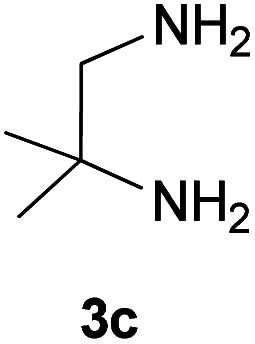	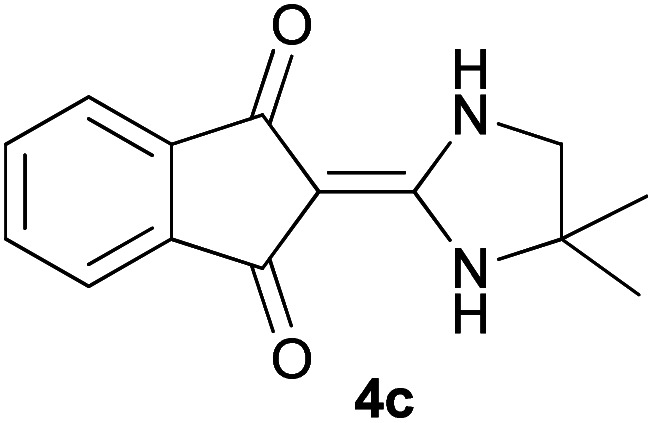	30	85	280–282
4	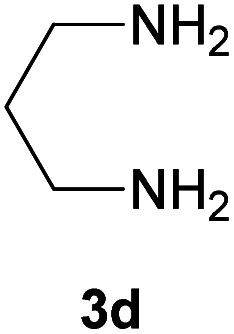	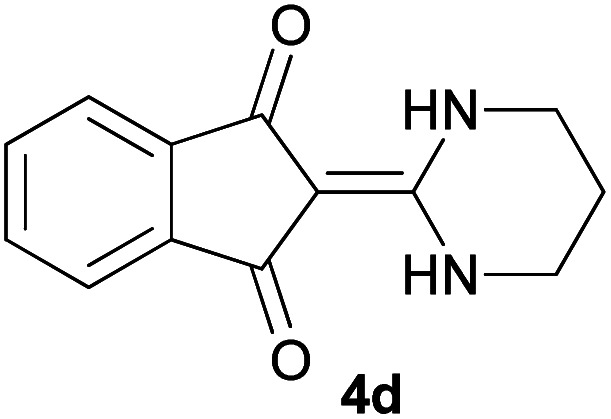	15	80	231–233
5	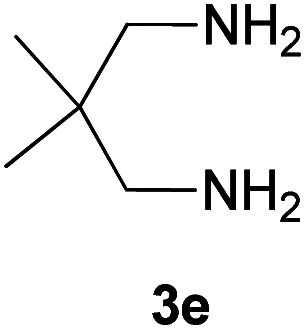	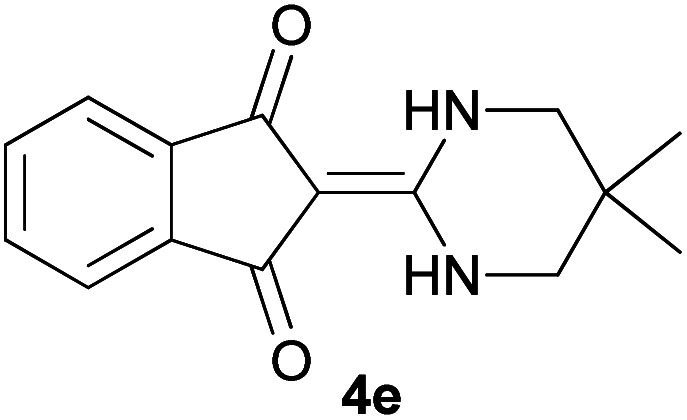	20	73	278–280
6	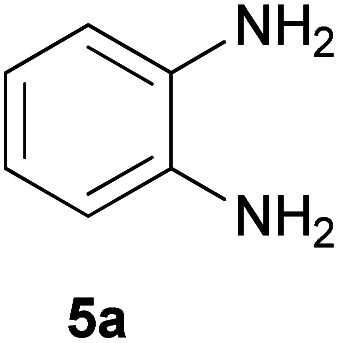	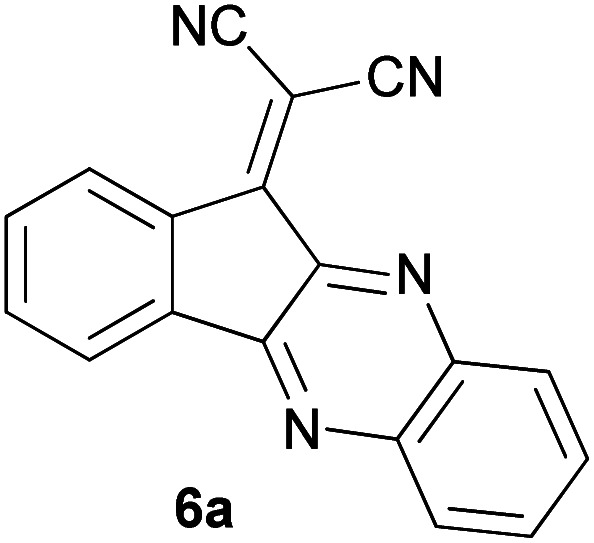	120	84	314–317
7	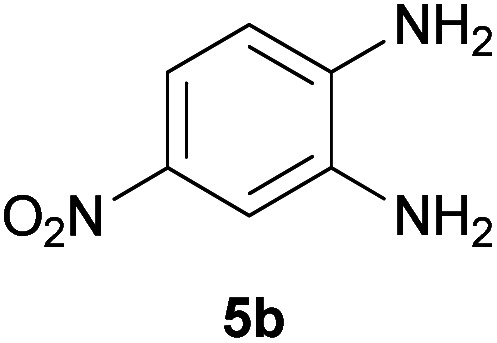	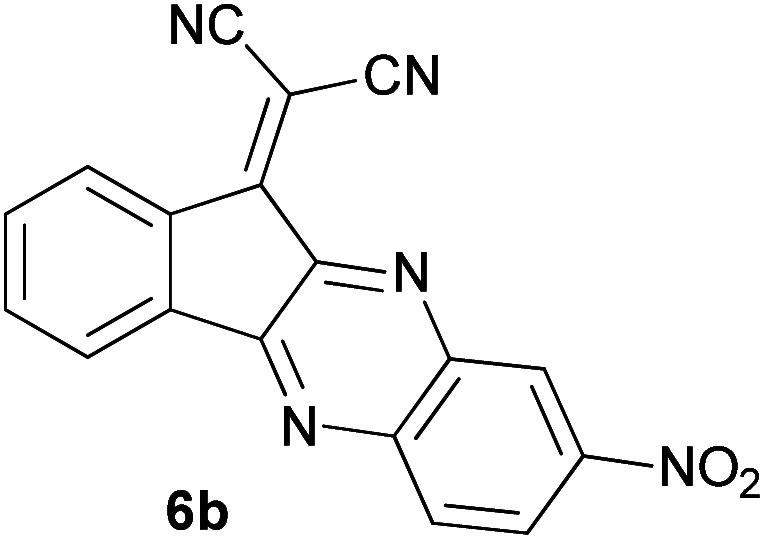	90	82	241–243
8	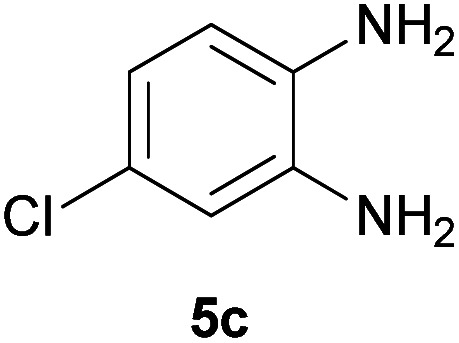	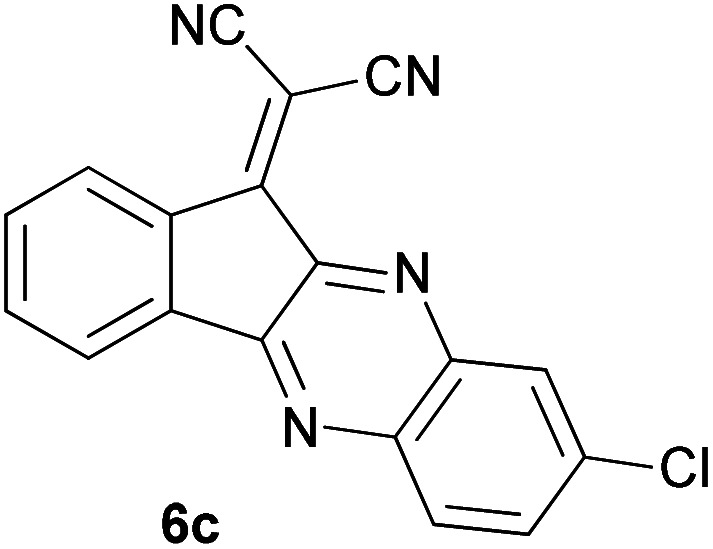	100	78	185–188

a The reactions were performed using ninhydrin (1 mmol), malononitrile (1 mmol), diamine (1 mmol), H_2_O (10 ml).

In the reaction with aryl diamines, it was initially expected that enaminone structures would be formed as in the reaction with linear diamines. But with the release of malononitrile instead of two nitrile groups, indenoquinoxalines are formed instead of benzimidazoles. It seems that in the case of aryl diamines, the formation of imine bonds is preferable to the formation of enaminones.

In fact, quinoxaline products 6 are obtained as a result of the primary elimination of malononitrile by the attack of the amine groups on the carbonyl groups. It can be said that stability due to aromaticity is the reason why these products are preferred over benzimidazole structures.

### Scope and limitations

The reaction was performed with other derivatives of linear diamines (1,4-diaminobutane and 1,2-diaminocyclohexane) under the same conditions, which did not lead to the product. Also the use of other active methylene compounds, ethyl and methyl cyanoacetate, instead of malononitrile resulted in no product formation.

**Scheme 2:**
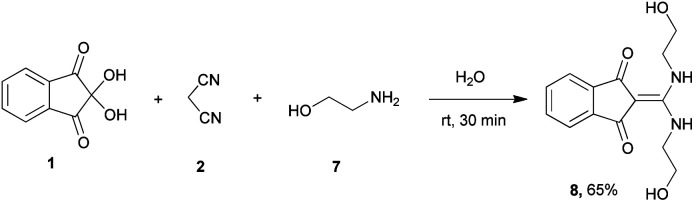
Synthetic scheme for the generation of product 8.

Based on these results, it was expected that the reaction with ethanolamine and cysteamine would also lead to the formation of oxazolidine and thiazolidine structures respectively. But the spectral data showed that the reaction with ethanolamine under the same conditions, produces the open chain enaminone product 8 (Scheme 2). Even when one-to-one ratios of starting materials were used, the same product 8 was obtained. The reaction with cysteamine did not lead to product formation.

Next, it was tried to perform the reaction with mono-functional aliphatic amines such as ethyl amine and propyl amine. Unfortunately, these reactions resulted in no identifiable product formation.

**Scheme 3:**
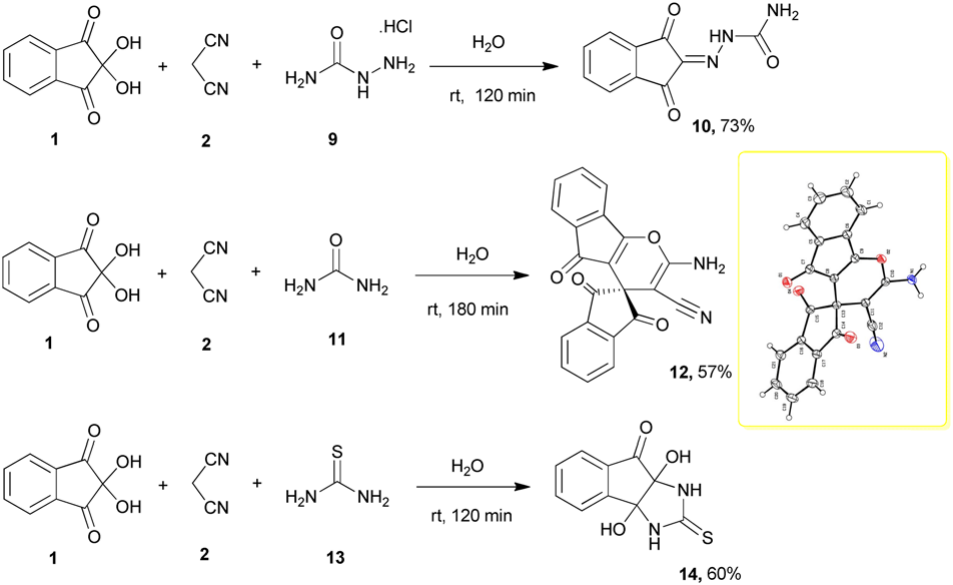
Synthetic scheme for the generation of products 10, 12, 14.

In the following, we tried to study the process of the mentioned reactions by changing the third component to obtain various products. Therefore, we performed the reactions with semicarbazide 9, urea 11 and thiourea 13. The results are shown in Scheme 3.

As can be seen, the product of each reaction is different from the other. In the reaction with semicarbazide hydrochloride 9, 2-(1,3-dioxo-1*H*-inden-2(3*H*)-ylidene)hydrazinecarboxamide 10 was formed. The reaction with urea led to an unexpected product, spiro[indene-2,4′-indeno[1,2-*b*]pyran]-3′-carbonitrile 12 with the participation of two moles of ninhydrin. Unambiguous evidence for this structure was obtained from single-crystal X-ray analyses. The ORTEP diagram of 12 is shown in Scheme 3.

The product of the three-component reaction with thiourea 13 was dihydroxy-2-thioxo-tetrahydroindeno[1,2-*d*]imidazolone 14. These reactions were also carried out in water at room temperature and led to the mentioned products with good yields.

It is necessary to mention this point that in all reported reactions, the method of adding the reactants is sequential. That is, first, ninhydrin and malononitrile were mixed together for 3 minutes and then the third component was added to it. More details are explained in the mechanism section.

Based on the result obtained in the synthesis of spiropyran 12, we decided to carry out the reaction with two components, ninhydrin and malononitrile, in the ratio of 2 to 1 under the same conditions. From the comparison of TLC and melting points, it was determined that product 12 is formed again.

### Structure determination

The formation of suggested products was clearly verified by IR, ^1^H NMR, ^13^C NMR spectroscopic and mass spectrometric data of the crude products (see the ESI†).

**Fig. 2:**
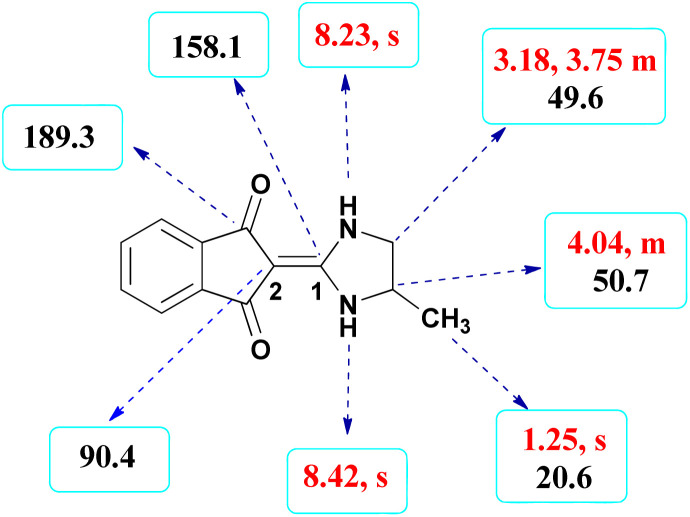
^1^H and ^13^C NMR chemical shifts of 4b.

Here we investigate the key signals of ^1^H and ^13^C NMR chemical shifts of product 4b as a representative case in Fig. 2. The ^1^H NMR spectrum of 4b showed two signals at *δ* 8.42 and 8.23 ppm identified as two NH groups. These peaks were exchangeable with D_2_O. The protons of aromatic ring were seen at *δ* 7.50–7.55 ppm. The signal at *δ* 4.04 was related to proton of the C–H group. Two protons of methylene group appeared at *δ* 3.75 and 3.18 ppm. The protons of the CH_3_ were observed at *δ* 1.25 ppm.

The ^1^H-decoupled ^13^C NMR spectrum of 4b indicated 9 distinct resonances in accordance to desired product: two signals of olefinic carbons (C-1 and C-2) were observed at *δ* 158.1 and 90.4 ppm respectively. The signals at *δ* 49.6 and 50.7 ppm were related to CH_2_ and CH carbons. The signals at *δ* 138.9, 132.0 and 119.9 ppm were assigned to carbons of aryl ring. The carbon of methyl group appeared at *δ* 20.6 ppm. The carbonyl groups were observed at *δ* 189.3 ppm (Fig. 2).

The mass spectrum of 4b showed a molecular-ion peak at *m*/*z* 228 in agreement with the proposed product. The IR spectrum of 4b showed absorption bands at 3253 and 2956 cm^−1^ due to NH_2_ and aliphatic CH groups respectively. Strong absorption of carbonyl groups was seen at 1626 cm^−1^ (see the corresponding spectra on pages 8–12 in the ESI†).

### Mechanism

**Scheme 4:**
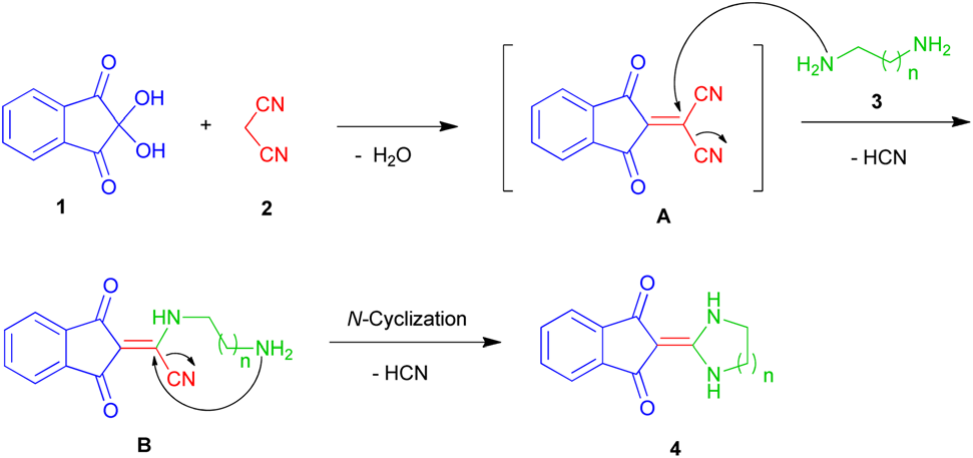
Proposed mechanism for the formation of products 4.

A reasonable mechanism for the formation of imidazolidin/tetrahydropyrimidine-2-ylidene-1,3-indenediones 4 is shown in Scheme 4. Initially, addition of malononitrile 2 to ninhydrin 1 leads to the formation of Knoevenagel product A. Then, nucleophilic attack of ethylenediamine 3a to C-1 in A leads to the intermediate B with HCN elimination. This intermediate then undergoes intramolecular cyclization *via* nucleophilic addition of the NH_2_ to C-1 followed by removing another HCN molecule to form the final product 4 (Scheme 4).

It should be noted that in the method of performing all reactions, first, ninhydrin and malononitrile were mixed in water for 3 minutes, which led to the formation of a yellow precipitate. Then the third component (*N*-binucleophilic compound) was added to it. Therefore, the formation of 2-(dicyanomethylene)-1,3-indandione A is expected as a product of the first step.

**Scheme 5:**
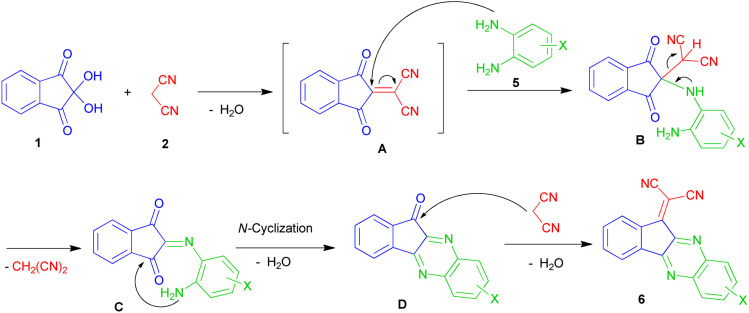
Proposed mechanism for the formation of products 6.

The formation of compounds 6 can be rationalized on the basis of extrusion of a dicyanomethylene molecule from the adduct A by the nucleophilic attack of 5 followed by the closure of the pyrazine ring and Knoevenagel condensation of D with malononitrile (Scheme 5).

**Scheme 6:**
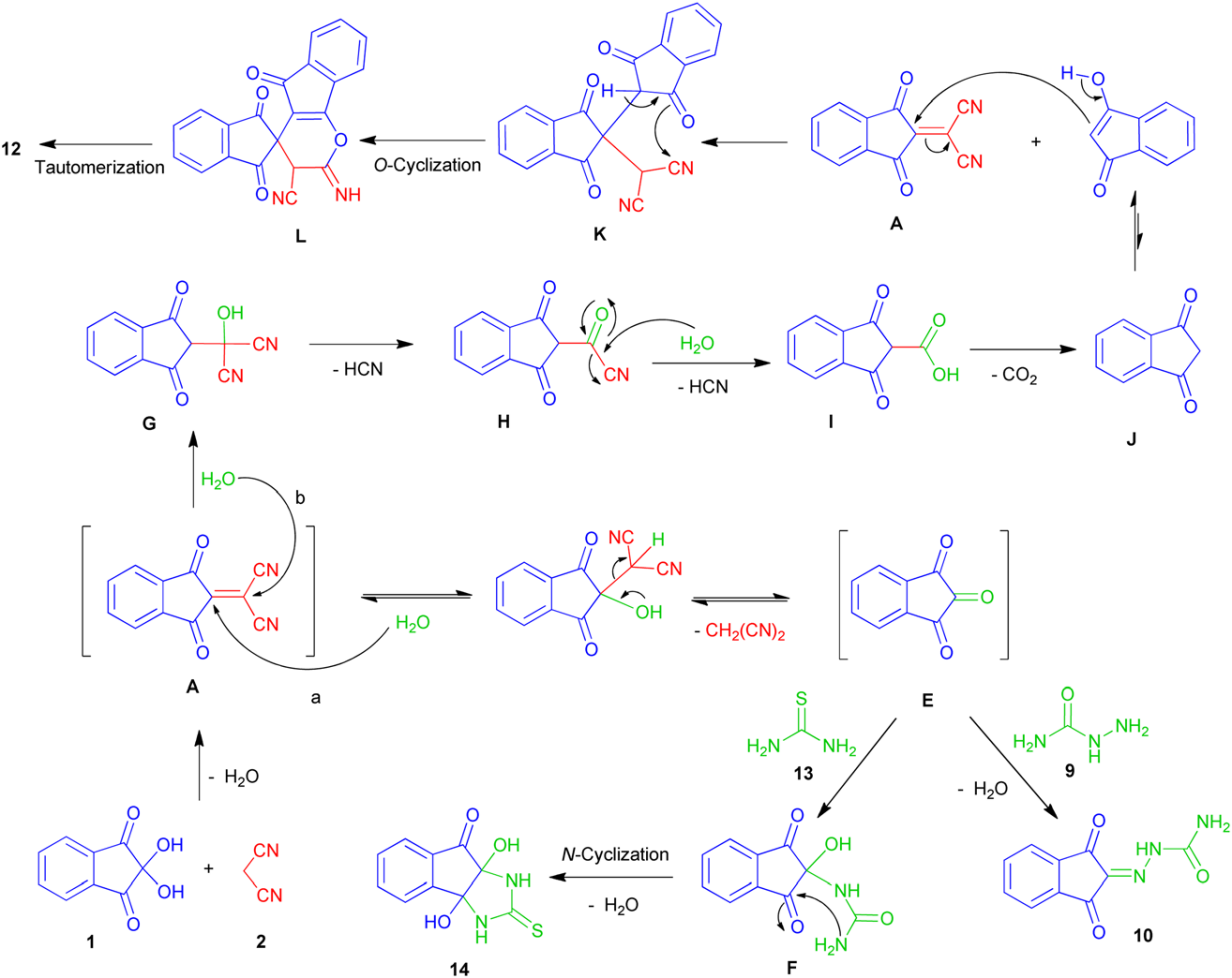
Proposed mechanism for the formation of products 10, 12, 14.

The proposed mechanism for the synthesis of products 10, 12 and 14 is presented in Scheme 6. After the formation of the Knoevenagel product A, the presence of water can cause two different paths. In pathway (a), the attack of water on C-2 and the subsequent release of malononitrile leads to the formation of indane-1,2,3-trione E. This intermediate reacts with semicarbazide to produce hydrazinecarboxamide 10. The reaction between E and thiourea 13, affords dihydroxy indenoimidazole 14 through intermediate F.

In path (b), the attack of water on C-1 followed by the release of hydrogen cyanide leads to the intermediate H. Hydrolysis of H to carboxylic acid I and elimination of carbon dioxide, produces 1,3-indanedione J. The attack of the enolic form of J on adduct A, leads to the intermediate K. Next, intramolecular cyclization and finally imin-enamine tautomerization affords the spiropyran 12.

Probably due to the weak nucleophilic nature of the amidic nitrogen of urea (compared to thiourea), the mechanism in the presence of urea proceeds from the path of indanedione formation (path b), and leads to the unexpected product 12. In fact, the competition between water and *N*-binucleophiles, as well as the existence of different positions for the attack of each, has led to the synthesis of various products.

## Conclusion

In summary, we have disclosed a convenient one-pot synthesis of novel heterocyclic scaffolds containing imidazolidin-2-ylidene-indenedione, tetrahydropyrimidine-2-ylidene-indenedione and indenoquinoxaline derivatives utilizing readily available starting materials. These reactions between ninhydrin, malononitrile, and various selected diamines were effectively accomplished in aqueous medium at room temperature and in a highly chemoselective manner. In addition, we were able to synthesize three other products, indenylidene hydrazinecarboxamide, spiroindene-indenopyran and dihydroxy-2-thioxo-indenoimidazolone under the same conditions. This catalyst free approach can be considered as environmentally friendly, since it uses water as the reaction medium and purification is performed by simple filtration, avoiding the use of organic solvents at any point of the experimental procedure.

## Experimental

### Materials

All commercially available reagents and other solvents were purchased from Aldrich and Merck Chemical Co. and used without further purification. The NMR spectra were recorded with a Bruker DRX-300 AVANCE instrument (300 MHz for ^1^H and 75.4 MHz for ^13^C) with DMSO-*d*_6_ as solvent. Chemical shifts are given in ppm (*δ*) and coupling constant (*J*) is reported in Hertz (Hz). Melting points were measured with an electrothermal 9100 apparatus. Mass spectra were recorded with an Agilent 5975C VL MSD with Triple-Axis detector operating at an ionization potential of 70 eV. IR spectra were measured with Bruker Tensor 27 spectrometer.

### General procedure of the synthesis of imidazolidin-2-ylidene-indenedione, pyrimidine-2-ylidene-indenedione 4a–e, indenoquinoxaline 6a–c and bis-hydroxyethyl aminomethylene-indenedione 8

The stoichiometric mixture of ninhydrin 1 (1 mmol, 0.178 g) and malononitrile 2 (1 mmol, 0.66 g) in H_2_O (10 ml) was stirred at room temperature. A yellow precipitate was formed and the reaction was completed within 3 minutes. Then 1 mmol of the third component (linear or aromatic diamine or ethanolamine) was added to the mixture. The progress of the reaction was monitored by TLC using ethyl acetate/*n*-hexane (1 : 1). After completion of the reaction, without the need for chromatography or recrystallization, the precipitated product was collected by filtration and washed with a mixture of water and ethanol (1 : 1) to give the pure products 4, 6 in high yields.

### Procedure of the synthesis of 1,3-dioxoindenylidene-hydrazinecarboxamide 10

A mixture of ninhydrin 1 (1 mmol, 0.178 g) and malononitrile 2 (1 mmol, 0.66 g) in H_2_O (10 ml) was stirred at room temperature. After 3 minutes, semicarbazide hydrochloride 9 (1 mmol, 0.111 g) was added to it. After 2 hours, TLC shows the consumption of the starting components. So the crude product was isolated with simple filtration and washed with H_2_O/EtOH (1 : 1).

### Procedure of the synthesis of spiroindene-indenopyran 12 and 2-thioxo-tetrahydroindeno-imidazolone 14

A mixture of ninhydrin 1 (1 mmol, 0.178 g) and malononitrile 2 (1 mmol, 0.66 g) in H_2_O (10 ml) was stirred at room temperature for 3 minutes. Then urea 11 or thiourea 13 was added to it. After the completion of the reaction, which was determined by TLC, the precipitated product was collected by means of filtration and washed with H_2_O/EtOH (1 : 1) to give the pure products 12, 14.

#### 2-(Imidazolidin-2-ylidene)-1*H*-indene-1,3(2*H*)-dione (4a)

Dark orange solid; yield: 0.209 g (98%); mp: 227–229 °C; IR (KBr) (*ν*_max_/cm^−1^): 3317, 2962, 1681, 1618, 1428, 1352, 1284, 1033, 721; ^1^H NMR (300 MHz, DMSO): *δ* 3.61 (4H, s, 2CH_2_), 7.47–7.56 (4H, m, ArH), 8.27 (2H, s, 2NH); ^13^C{^1^H} NMR (75.4 MHz, DMSO): *δ* 42.7 (2CH_2_), 90.5 (**C**=C–NH), 119.9, 132.0, 138.9 (Ar), 159.0 (C=**C**–NH), 189.2 (C=O); *m*/*z* (%) = 215 (16) [M + 1]^+^, 214 (100) [M]^+^, 213 (39), 199 (2), 185 (29), 158 (26), 130 (7), 102 (15), 76 (5).

#### 2-(4-Methylimidazolidin-2-ylidene)-1*H*-indene-1,3(2*H*)-dione (4b)

Yellow solid; yield: 0.173 g (76%); mp: 240–242 °C; IR (KBr) (*ν*_max_/cm^−1^): 3253, 2956, 1622, 1439, 1290, 869, 733; ^1^H NMR (300 MHz, DMSO): *δ* 1.25 (3H, d, *J* = 6 Hz, CH_3_), 3.18 (1H, dd, CH_2_), 3.75 (1H, t, *J* = 9 Hz, CH_2_), 4.01–4.06 (4H, m, CH), 7.50–7.55 (4H, m, ArH), 8.23 (1H, s, NH), 8.42 (1H, s, NH); ^13^C{^1^H} NMR (75.4 MHz, DMSO): *δ* 20.6 (CH_3_), 49.6 (CH_2_), 50.7 (CH), 90.4 (**C**=C–NH), 119.9, 132.0, 138.9 (Ar), 158.1 (C=**C**–NH), 189.3 (C=O); *m*/*z* (%) = 229 (15) [M + 1]^+^, 228 (91) [M]^+^, 213 (100), 199 (3), 185 (8), 158 (15), 130 (5), 102 (10), 76 (5).

#### 2-(4,4-Dimethylimidazolidin-2-ylidene)-1*H*-indene-1,3(2*H*)-dione (4c)

Dark yellow solid; yield: 0.205 g (85%); mp: 280–282 °C; IR (KBr) (*ν*_max_/cm^−1^): 3255, 2957, 2872, 1621, 1439, 1203, 1053, 780; ^1^H NMR (300 MHz, DMSO): *δ* 0.98 (6H, s, 2CH_3_), 3.08 (2H, s, CH_2_), 7.43–7.53 (4H, m, ArH), 8.69 (2H, s, 2NH); ^13^C{^1^H} NMR (75.4 MHz, DMSO): *δ* 23.8 (CH_3_), 26.1 (CH_2_), 48.7 (CMe_2_), 91.0 (**C**=C–NH), 119.7, 131.9, 138.5 (Ar), 154.5 (C=**C**–NH), 190.0 (C=O); *m*/*z* (%) = 256 (100), 242 (6) [M]^+^, 241 (37), 228 (6), 213 (6), 186 (4), 173 (25), 186 (5), 126 (4), 89 (7), 55 (3).

#### 2-(Tetrahydropyrimidin-2(1*H*)-ylidene)-1*H*-indene-1,3(2*H*)-dione (4d)

Gray solid; yield: 0.182 g (80%); mp: 231–233 °C; ^1^H NMR (300 MHz, DMSO): *δ* 1.86–1.91 (2H, m, CH_2_), 7.42–7.52 (4H, m, ArH), 8.63 (2H, s, 2NH); ^13^C{^1^H} NMR (75.4 MHz, DMSO): *δ* 19.3 (CH_2_), 37.6 (CH_2_NH), 91.3 (**C**=C–NH), 119.7, 131.9, 138.5 (Ar), 155.4 (C=**C**–NH), 190.0 (C=O); *m*/*z* (%) = 229 (17) [M + 1]^+^, 228 (100) [M]^+^, 227 (24), 199 (10), 172 (13), 144 (2), 114 (6), 76 (6).

#### 2-(5,5-Dimethyltetrahydropyrimidin-2(1*H*)-ylidene)-1*H*-indene-1,3(2*H*)-dione (4e)

Shiny green solid; yield: 0.186 g (73%); mp: 278–280 °C; ^1^H NMR (300 MHz, DMSO): *δ* 0.98 (6H, s, 2CH_3_), 3.08 (4H, s, 2CH_2_), 7.45–7.51 (4H, m, ArH), 8.68 (2H, s, 2NH); ^13^C{^1^H} NMR (75.4 MHz, DMSO): *δ* 23.9 (CH_3_), 26.2 (CMe_2_), 48.8 (CH_2_NH), 91.1 (**C**=C–NH), 119.7, 132.0, 138.5 (Ar), 154.6 (C=**C**–NH), 190.0 (C=O); *m*/*z* (%) = 257 (20) [M + 1]^+^, 256 (100) [M]^+^, 241 (36), 213 (6), 173 (25), 154 (4), 104 (6), 76 (6).

#### 2-(11*H*-Indeno[1,2-*b*]quinoxalin-11-ylidene)malononitrile (6a)

Orange solid; yield: 0.235 g (84%); mp: 314–317 °C; IR (KBr) (*ν*_max_/cm^−1^): 3433, 3073, 1725, 1573, 1337, 1191, 736; ^1^H NMR (300 MHz, DMSO): *δ* 7.57–8.17 (4H, m, ArH); *m*/*z* (%) = 281 (19) [M + 1]^+^, 280 (100) [M]^+^, 279 (21), 262 (42), 232 (4), 194 (7), 152 (6), 140 (8), 103 (9), 76 (12), 50 (9).

#### 2-(8-Nitro-11*H*-indeno[1,2-*b*]quinoxalin-11-ylidene)malononitrile (6b)

Light yellow solid; yield: 0.266 g (82%); mp: 241–243 °C; IR (KBr) (*ν*_max_/cm^−1^): 3435, 3073, 1727, 1572, 1535, 1338, 1193, 738; ^1^H NMR (300 MHz, DMSO): *δ* 7.58 (1H, s, ArH), 7.79–7.82 (1H, m, ArH), 7.90–7.96 (2H, m, ArH), 8.14–8.16 (1H, d, *J* = 6 Hz, ArH), 8.33 (1H, d, *J* = 9 Hz, ArH), 8.56–8.60 (1H, m, ArH); ^13^C{^1^H} NMR (75.4 MHz, DMSO): *δ* 86.0 (C=**C**–CN), 112.5 (CN), 113.6 (CN), 124.3, 124.4, 127.0, 131.5, 135.8, 136.7, 137.7, 141.3, 149.6, 150.2 (Ar), 153.7 (C=N), 159.3 (C=N), 182.8 (**C**=C–CN); *m*/*z* (%) = 326 (21) [M + 1]^+^, 325 (100) [M]^+^, 295 (23), 279 (43), 240 (9), 225 (16), 175 (8), 146 (6), 102 (6), 75 (19), 51 (8).

#### 2-(8-Chloro-11*H*-indeno[1,2-*b*]quinoxalin-11-ylidene)malononitrile (6c)

Yellowish brown solid; yield: 0.244 g (78%); mp: 185–188 °C; IR (KBr) (*ν*_max_/cm^−1^): 3434, 3076, 2226, 1727, 1552, 1495, 1335, 1169, 1069, 836, 732; ^1^H NMR (300 MHz, DMSO): *δ* 7.83–7.90 (4H, m, ArH), 8.07–8.22 (4H, m, ArH); *m*/*z* (%) = 316 (36) [M + 2]^+^, 315 (25) [M + 1]^+^, 314 (100) [M]^+^, 279 (14), 252 (3), 202 (1), 178 (2), 157 (5), 100 (4), 75 (7), 50 (2).

#### 2-(Bis((2-hydroxyethyl)amino)methylene)-1*H*-indene-1,3(2*H*)-dione (8)

Yellow solid; yield: 0.179 g (65%); mp: 224–226 °C; IR (KBr) (*ν*_max_/cm^−1^): 3355, 3213, 1636, 1450, 1175, 1072, 749; ^1^H NMR (300 MHz, DMSO): *δ* 3.59 (8H, s, 4CH_2_), 5.08 (2H, s, 2OH), 7.42–7.54 (4H, m, ArH), 9.01 (2H, s, 2NH); ^13^C{^1^H} NMR (75.4 MHz, DMSO): *δ* 46.1 (2CH_2_NH), 59.8 (2CH_2_OH), 92.9 (**C**=C–NH), 119.7, 132.1, 138.2 (Ar), 159.6 (C=**C**–NH), 190.2 (C=O); *m*/*z* (%) = 277 (7) [M + 1]^+^, 276 (40) [M]^+^, 258 (43), 233 (32), 216 (47), 189 (32), 172 (100), 126 (7), 105 (13), 89 (17), 62 (3).

#### 2-(1,3-Dioxo-1*H*-inden-2(3*H*)-ylidene)hydrazinecarboxamide (10)

Yellowish brown solid; yield: 0.153 g (72%); mp: 204 °C (decompose); IR (KBr) (*ν*_max_/cm^−1^): 3324, 3226, 1685, 1572, 1446, 1350, 1190, 749; ^1^H NMR (300 MHz, DMSO): *δ* 7.40 (2H, s, NH_2_), 7.96 (4H, s, ArH), 12.00 (1H, s, NH); ^13^C{^1^H} NMR (75.4 MHz, DMSO): *δ* 123.1, 123.4, 134.0, 136.1, 136.2, 139.2 (Ar), 140.8 (C=N), 153.9 (CONH_2_), 185.5 (C=O), 186.7 (C=O); *m*/*z* (%) = 217 (3) [M]^+^, 208 (73), 173 (29), 146 (100), 105 (63), 76 (50), 50 (19).

#### 2′-Amino-1,3,5′-trioxo-1,3-dihydro-5′*H*-spiro[indene-2,4′-indeno[1,2-*b*]pyran]-3′-carbonitrile (12)

Orange solid; yield: 0.201 g (57%); mp: 238 °C (decompose); IR (KBr) (*ν*_max_/cm^−1^): 3389, 1706, 1582, 1355, 1240, 746; ^1^H NMR (300 MHz, DMSO): *δ* 7.33 (1H, d, *J* = 6 Hz, ArH), 7.39 (1H, d, *J* = 6 Hz, ArH), 7.47 (1H, m, ArH), 7.57–7.62 (1H, m, ArH), 8.10 (2H, s, NH_2_), 8.15 (4H, s, ArH); ^13^C{^1^H} NMR (75.4 MHz, DMSO): *δ* 51.6 (C=**C**–CN), 53.1 (C_spiro_), 105.5 (**C**=C–O), 116.8 (CN), 119.2, 122.6, 123.8, 129.9, 131.8, 134.0, 134.6, 137.7, 140.6 (Ar), 161.4 (C=**C**–O), 169.0 (**C**=C–CN), 189.3 (C=O), 198.4 (C=O); *m*/*z* (%) = 355 (8) [M + 1]^+^, 354 (35) [M]^+^, 326 (20), 271 (10), 243 (21), 214 (27), 176 (7), 139 (14), 104 (53), 76 (100), 50 (51).

#### 3*a*,8*a*-Dihydroxy-2-thioxo-1,3,3*a*,8*a*-tetrahydroindeno[1,2-*d*]imidazol-8(2*H*)-one (14)

White solid; yield: 0.141 g (60%); mp: 219–221 °C; IR (KBr) (*ν*_max_/cm^−1^): 3306, 3182, 1724, 1600, 1507, 1444, 1234, 1106, 893, 613; ^1^H NMR (300 MHz, DMSO): *δ* 6.85 (2H, br s, 2OH), 7.59–7.64 (1H, m, ArH), 7.76–7.91 (3H, m, ArH), 9.54 (1H, s, NH), 9.82 (1H, s, NH); ^13^C{^1^H} NMR (75.4 MHz, DMSO): *δ* 89.7 (C–OH), 90.2 (C–OH), 123.7, 125.5, 130.7, 132.5, 137.1, 150.9 (Ar), 178.3 (C=S), 196.5 (C=O); *m*/*z* (%) = 237 (4) [M + 1]^+^, 236 (33) [M]^+^, 208 (6), 177 (25), 132 (38), 104 (100), 90 (2), 76 (94), 50 (32).

## Conflicts of interest

There are no conflicts to declare.

## Supplementary Material

RA-012-D2RA06469C-s001
