# Supplementary material for: New multicomponent reactions in water: a facile synthesis of 1,3-dioxo-2-indanilidene-heterocyclic scaffolds and indenoquinoxalines through reaction of ninhydrin-malononitrile adduct with diverse N-binucleophiles
Source: RSC Adv. 2022 Nov 24;12(52):33772–9. doi: 10.1039/d2ra06469c (PMC9685738; doi:10.1039/d2ra06469c)
Supplement: RA-012-D2RA06469C-s001 [file RA-012-D2RA06469C-s001.pdf]

Electronic Supplementary Material (ESI) for RSC Advances.
This journal is © The Royal Society of Chemistry 2022

Supporting Information

New multicomponent reactions in water: A facile synthesis of 1,3-dioxo-2-indanilidene-heterocyclic scaffolds and indenoquinoxalines through reaction of ninhydrin-malononitrile adduct with diverse *N*-binucleophiles

Zahra Rahimi, Mohammad Bayat,* Hajar Hosseini

Department of Chemistry, Faculty of Science, Imam Khomeini International University, Qazvin, Iran.

E-mail: bayat_mo@yahoo.com, m.bayat@sci.ikiu.ac.ir

Table of Contents

Title	Page
Title, author's name, address and table of contents	1
Experimental Section; General remarks	2
**Figure 1.** Structure of all products **4,6,8,10,12,14**	3
^{1}H and ^{13}C NMR and IR and Mass spectrums of **4a**	4-7
^{1}H and D_2O exchange and ^{13}C NMR and IR and Mass spectrums of **4b**	8-12
^{1}H and ^{13}C NMR and IR and Mass spectrums of **4c**	13-16
^{1}H and ^{13}C NMR and Mass spectrums of **4d**	17-19
^{1}H and ^{13}C NMR and Mass spectrums of **4e**	20-22
^{1}H and ^{13}C NMR and IR and Mass spectrums of **6a**	23-26
^{1}H and ^{13}C NMR and IR and Mass spectrums of **6b**	27-30
^{1}H and ^{13}C NMR and IR and Mass spectrums of **6c**	31-34
^{1}H and D_2O exchange and ^{13}C NMR and IR and Mass spectrums of **8**	35-39
^{1}H and D_2O exchange and ^{13}C NMR and IR and Mass spectrums of **10**	40-44
**Figure 2.** The X-ray structure of compound **12**	45
^{1}H and ^{13}C NMR and IR and Mass spectrums of **12**	46-49
^{1}H and ^{13}C NMR and IR and Mass spectrums of **14**	50-53

Experimental Section

General remarks:

All commercially available reagents and other solvents were purchased from Aldrich and Merck chemical Co. and used without further purification. The NMR spectra were recorded with a Bruker DRX-300 AVANCE instrument (300 MHz for ^{1}H and 75.4 MHz for ^{13}C) with DMSO-d_6 as solvent. Chemical shifts are given in ppm (δ) and coupling constants (J) are reported in Hertz (Hz). Melting points were measured with an electrothermal 9100 apparatus. Mass spectra were recorded with an Agilent 5975C VL MSD with Triple-Axis detector operating at an ionization potential of 70 eV. IR spectra were measured with Bruker Tensor 27 spectrometer.

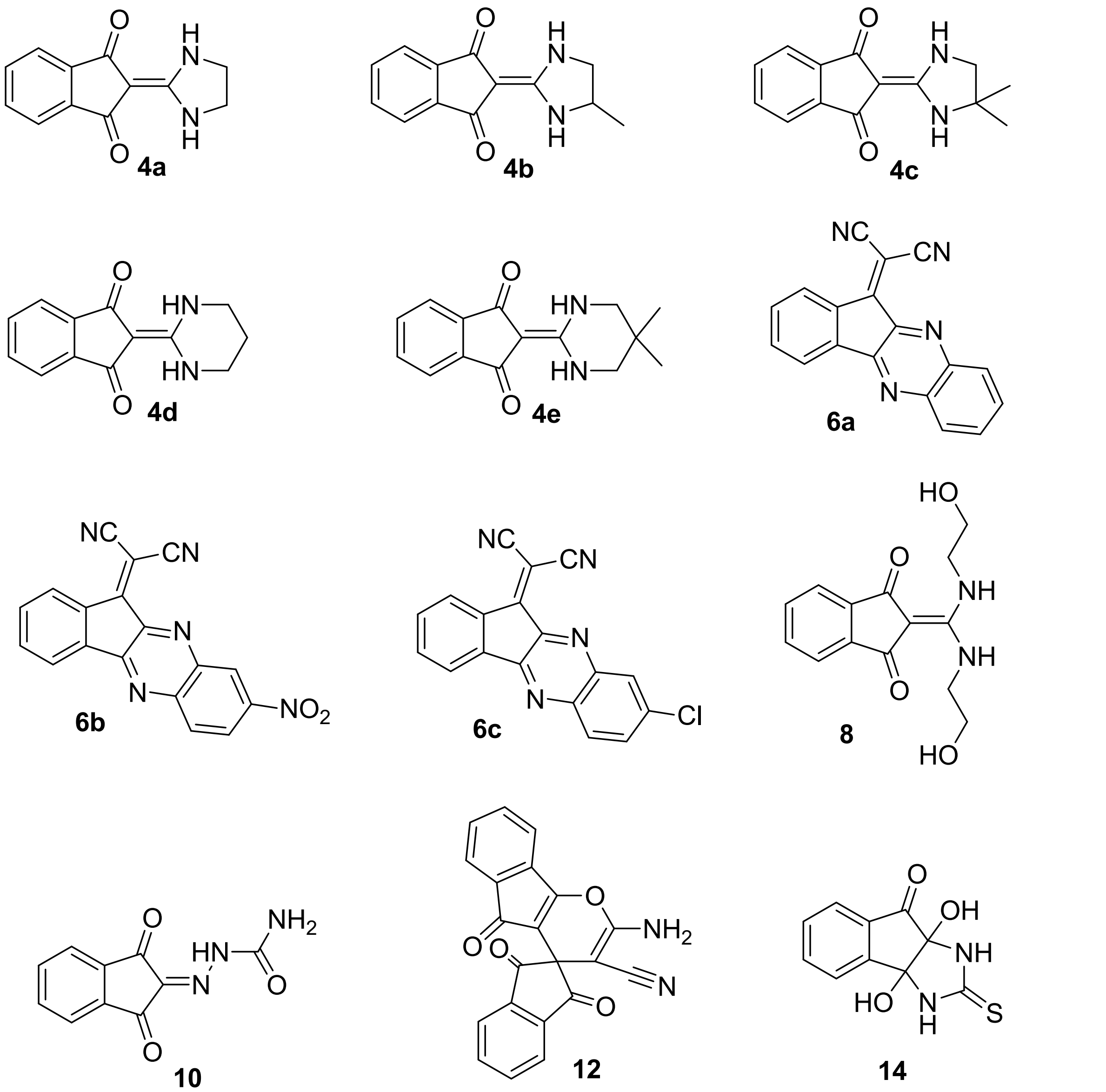

Figure 1. Structure of all products **4,6,8,10,12,14.**

The structure of the products were deduced from their IR, Mass, ^{1}H NMR, and ^{13}C NMR spectra (see the following images).

The ^{1}H and ^{13}C NMR spectra are taken in DMSO-d_6, but two derivatives **6a,6c** have low solubility, so they have no clear carbon spectra.

Parameter		Value
1	Data File Name	Z.Rahimi/ 131/ fid
2	Title	Z.Rahimi
3	Comment	G38 HNMR
4	Origin	UXNMR, Bruker Analytische Messtechnik GmbH
5	Owner	root
6	Site	
7	Spectrometer	spect
8	Author	
9	Solvent	DMSO
10	Temperature	300.0
11	Pulse Sequence	zg
12	Number of Scans	16
13	Receiver Gain	456
14	Relaxation Delay	2.0000
15	Pulse Width	9.0000
16	Acquisition Time	1.3665
17	Acquisition Date	2009-01-26T10:54:00
18	Modification Date	2009-01-26T10:55:52
19	Spectrometer Frequency	299.87
20	Spectral Width	5995.2
21	Lowest Frequency	4.4
22	Nucleus	1H
23	Acquired Size	8192
24	Spectral Size	16384

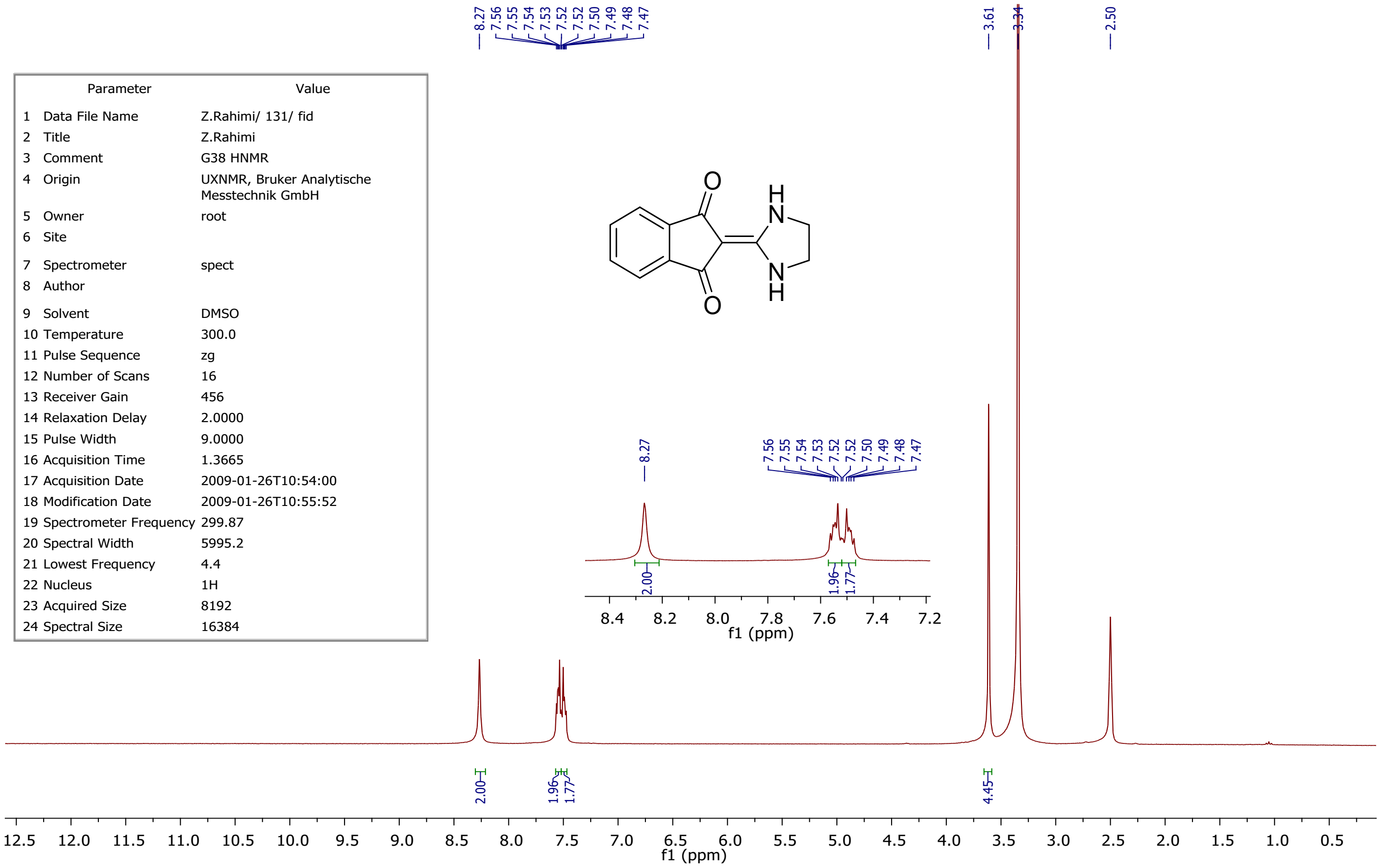

^{1}H NMR of 4a

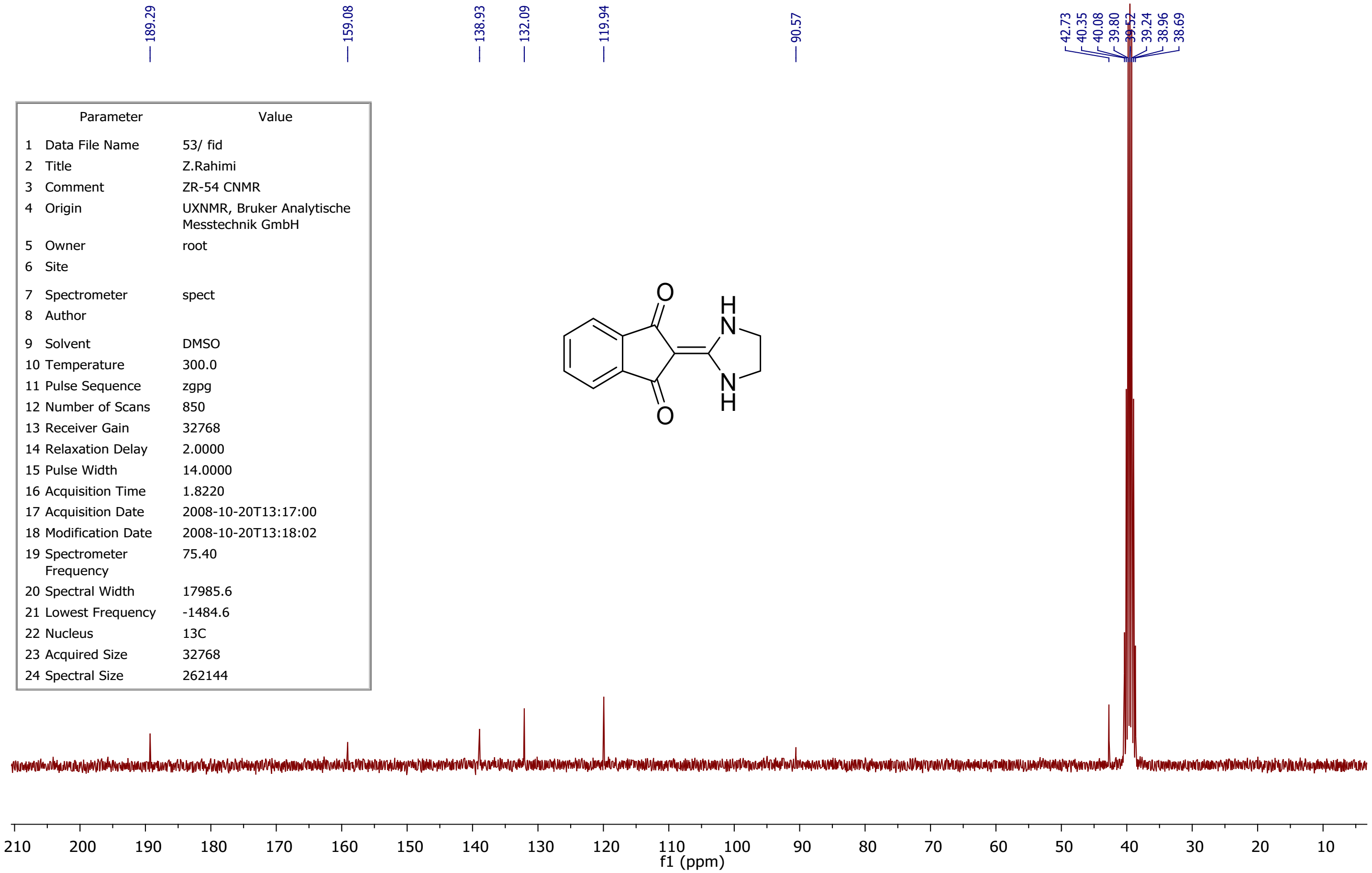
189.29
159.08
138.93
132.09
119.94
90.57
42.73
40.35
40.08
39.80
39.52
39.24
38.96
38.69
Parameter Value
1 Data File Name 53/ fid
2 Title Z.Rahimi
3 Comment ZR-54 CNMR
4 Origin UXNMR, Bruker Analytische Messtechnik GmbH
5 Owner root
6 Site
7 Spectrometer spect
8 Author
9 Solvent DMSO
10 Temperature 300.0
11 Pulse Sequence zgpg
12 Number of Scans 850
13 Receiver Gain 32768
14 Relaxation Delay 2.0000
15 Pulse Width 14.0000
16 Acquisition Time 1.8220
17 Acquisition Date 2008-10-20T13:17:00
18 Modification Date 2008-10-20T13:18:02
19 Spectrometer Frequency 75.40
20 Spectral Width 17985.6
21 Lowest Frequency -1484.6
22 Nucleus 13C
23 Acquired Size 32768
24 Spectral Size 262144
O
H
N
N
H
O
210 200 190 180 170 160 150 140 130 120 110 100 90 80 70 60 50 40 30 20 10
f1 (ppm)

^{13}C NMR of 4a

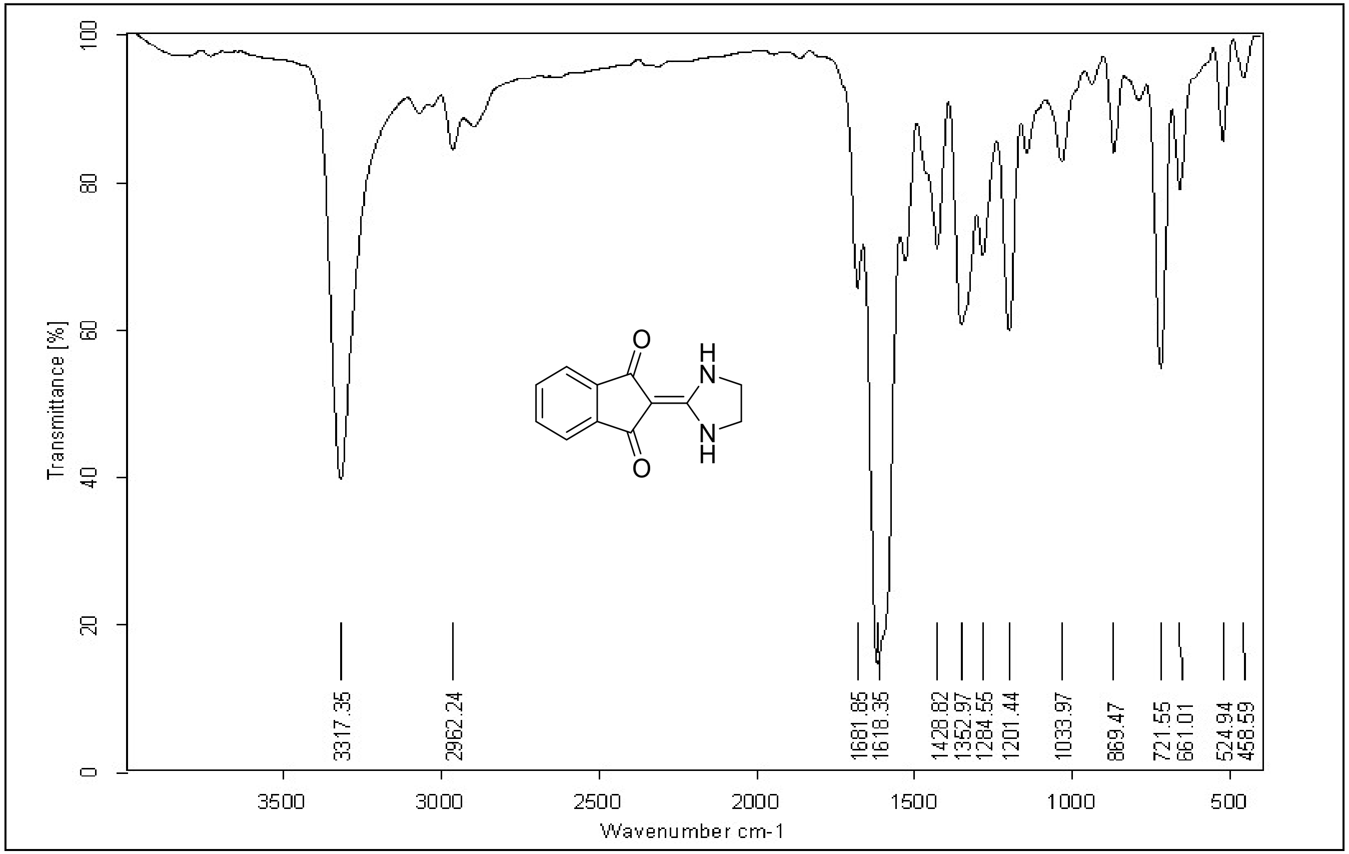
Transmittance [%]
Wavenumber cm-1
3317.35
2962.24
1681.85
1618.35
1428.82
1352.97
1284.55
1201.44
1033.97
869.47
721.55
661.01
524.94
458.59

IR of 4a

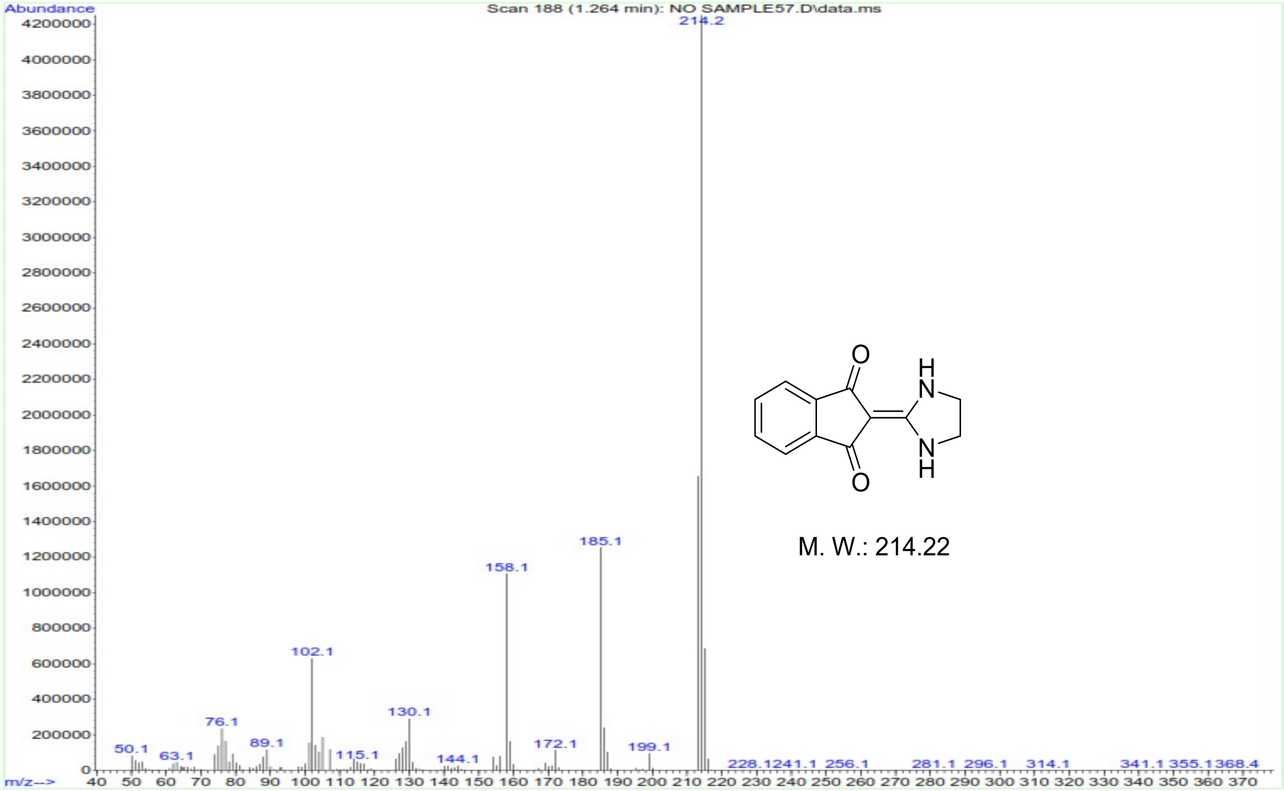
Abundance
Scan 188 (1.264 min): NO SAMPLE57.D\data.ms
214.2
185.1
158.1
102.1
130.1
76.1
89.1
50.1
63.1
115.1
144.1
172.1
199.1
228.1
241.1
256.1
281.1
296.1
314.1
341.1
355.1
368.4
m/z-->
M. W.: 214.22

MS of 4a

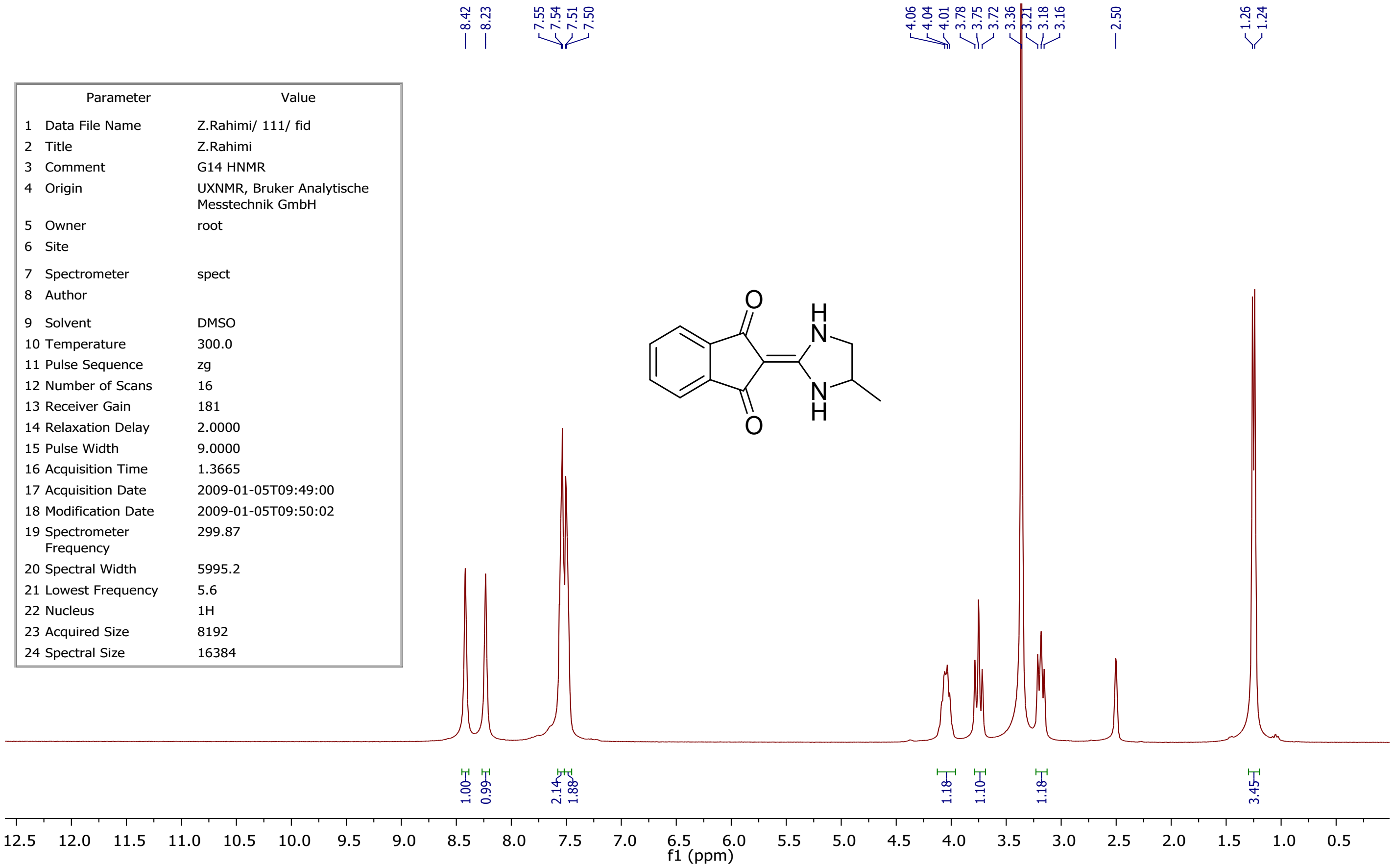

	Parameter	Value
1	Data File Name	Z.Rahimi/ 111/ fid
2	Title	Z.Rahimi
3	Comment	G14 HNMR
4	Origin	UXNMR, Bruker Analytische Messtechnik GmbH
5	Owner	root
6	Site	
7	Spectrometer	spect
8	Author	
9	Solvent	DMSO
10	Temperature	300.0
11	Pulse Sequence	zg
12	Number of Scans	16
13	Receiver Gain	181
14	Relaxation Delay	2.0000
15	Pulse Width	9.0000
16	Acquisition Time	1.3665
17	Acquisition Date	2009-01-05T09:49:00
18	Modification Date	2009-01-05T09:50:02
19	Spectrometer Frequency	299.87
20	Spectral Width	5995.2
21	Lowest Frequency	5.6
22	Nucleus	1H
23	Acquired Size	8192
24	Spectral Size	16384

^{1}H NMR of 4b

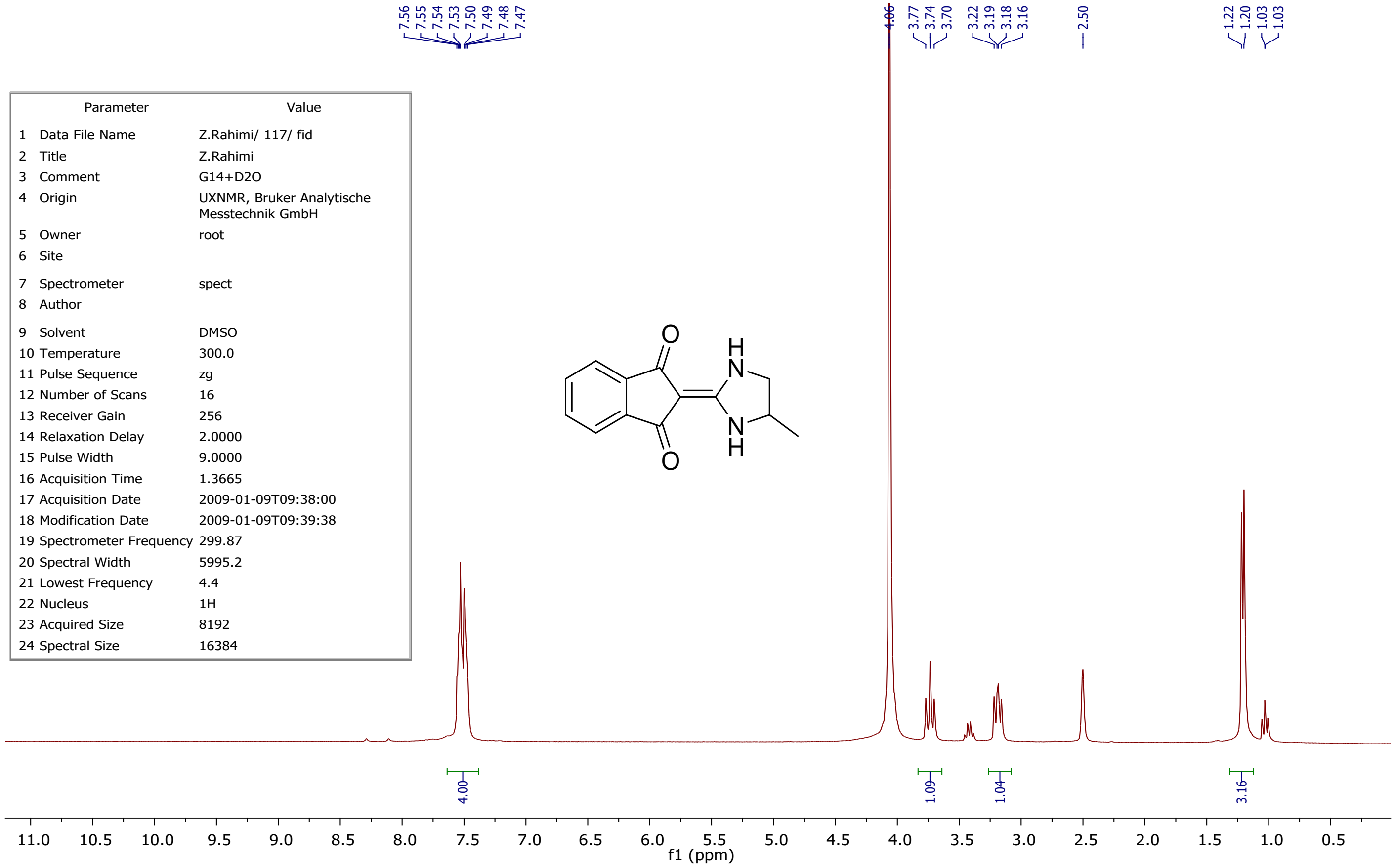

^{1}H-NMR with D_2O exchange of 4b

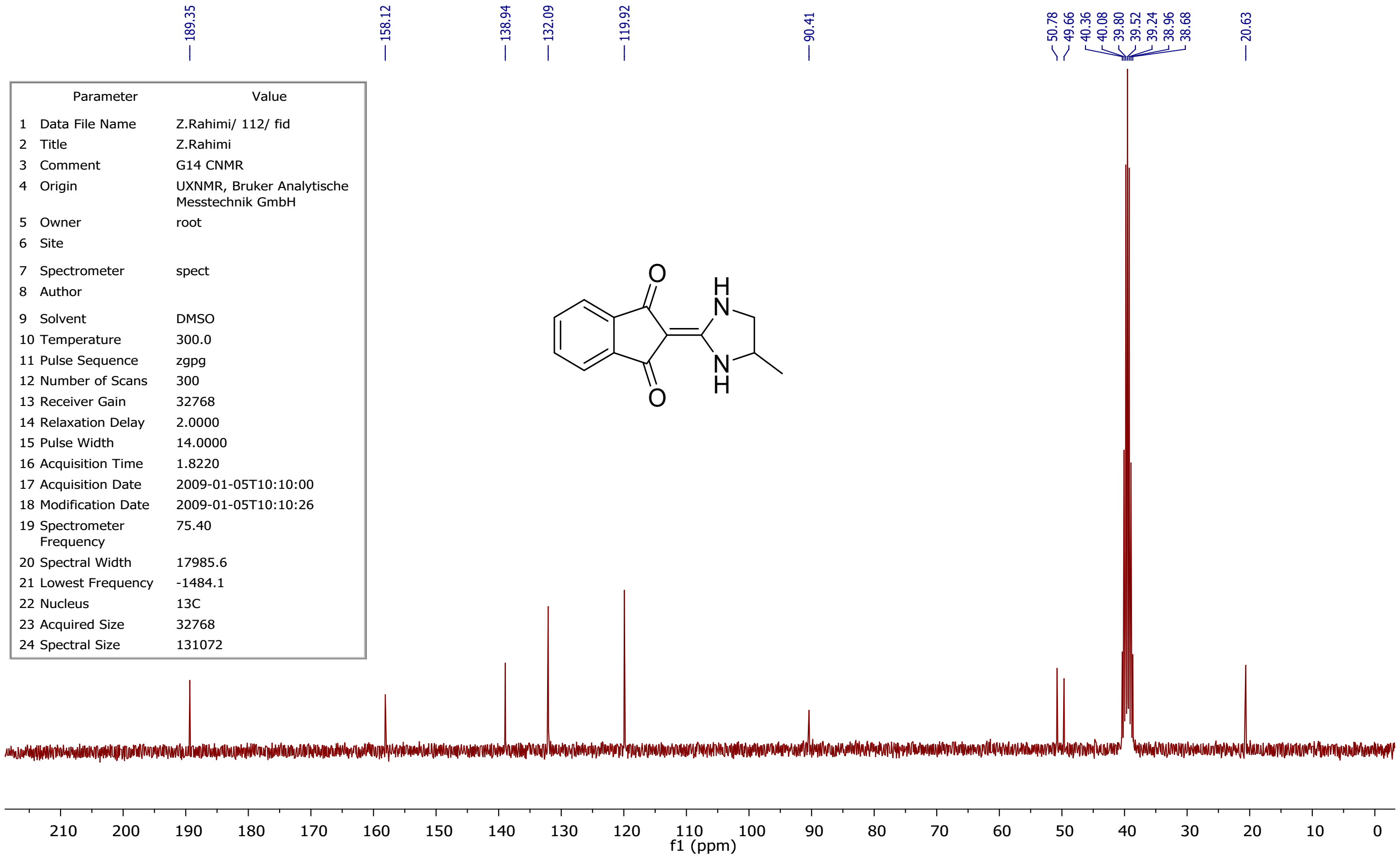

	Parameter	Value
1	Data File Name	Z.Rahimi/ 112/ fid
2	Title	Z.Rahimi
3	Comment	G14 CNMR
4	Origin	UXNMR, Bruker Analytische Messtechnik GmbH
5	Owner	root
6	Site	
7	Spectrometer	spect
8	Author	
9	Solvent	DMSO
10	Temperature	300.0
11	Pulse Sequence	zgpg
12	Number of Scans	300
13	Receiver Gain	32768
14	Relaxation Delay	2.0000
15	Pulse Width	14.0000
16	Acquisition Time	1.8220
17	Acquisition Date	2009-01-05T10:10:00
18	Modification Date	2009-01-05T10:10:26
19	Spectrometer Frequency	75.40
20	Spectral Width	17985.6
21	Lowest Frequency	-1484.1
22	Nucleus	13C
23	Acquired Size	32768
24	Spectral Size	131072

^{13}C NMR of 4b

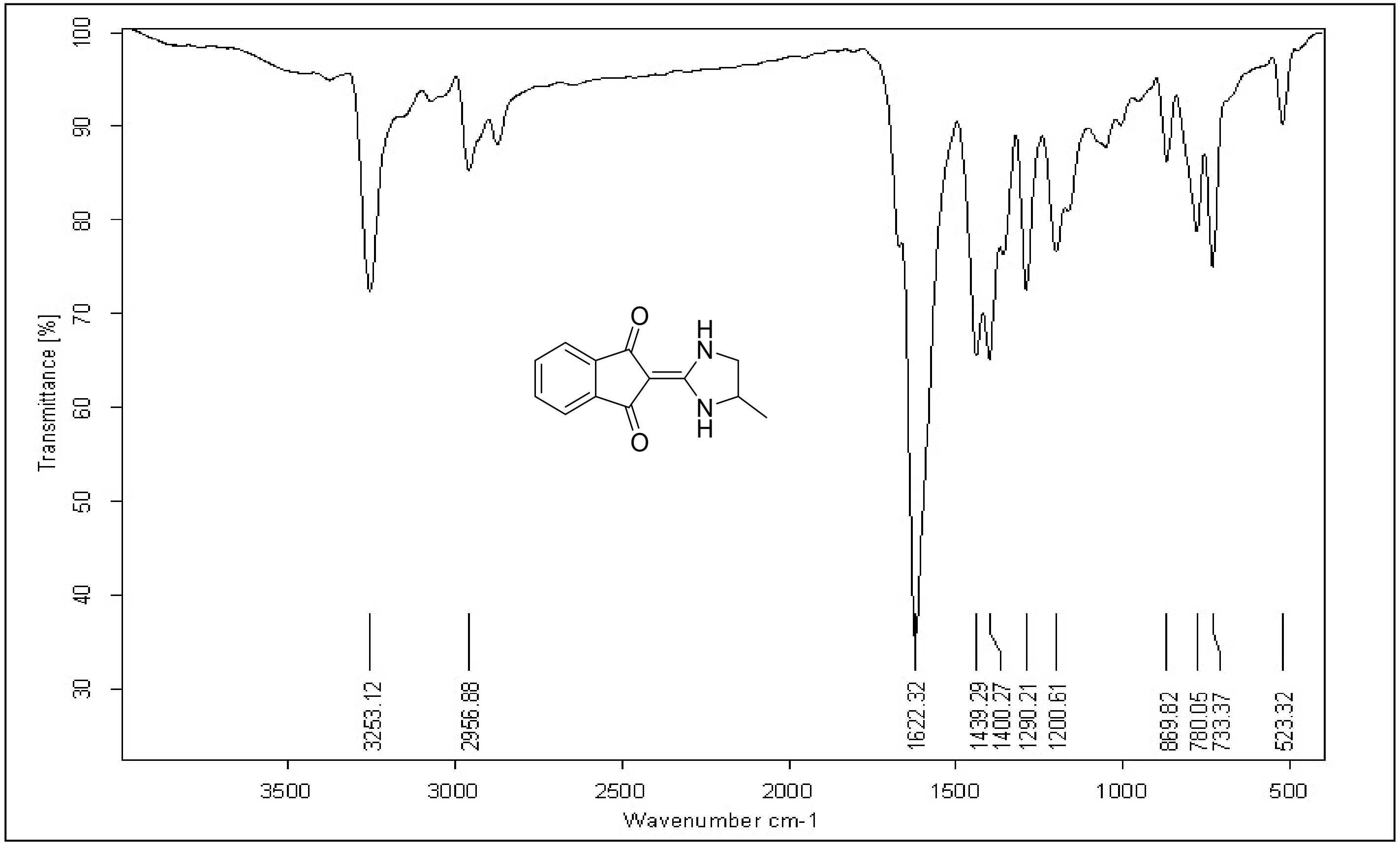
Transmittance [%]
100
90
80
70
60
50
40
30
3253.12
2956.88
1622.32
1439.29
1400.27
1290.21
1200.61
869.82
780.05
733.37
523.32
3500
3000
2500
2000
1500
1000
500
Wavenumber cm-1
O
O
H
N
N
H

IR of 4b

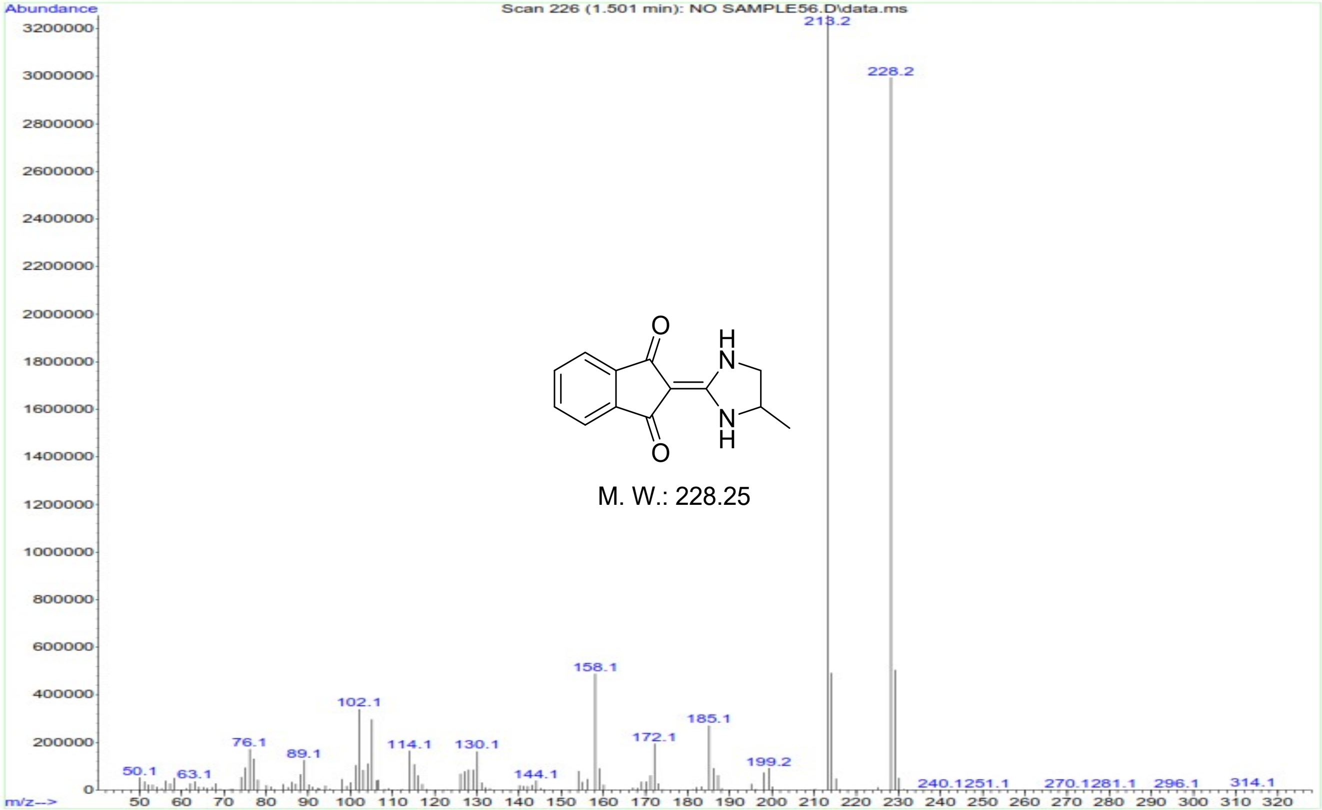
Abundance
Scan 226 (1.501 min): NO SAMPLE56.D\data.ms
3200000
3000000
2800000
2600000
2400000
2200000
2000000
1800000
1600000
1400000
1200000
1000000
800000
600000
400000
200000
0
m/z-->
50 60 70 80 90 100 110 120 130 140 150 160 170 180 190 200 210 220 230 240 250 260 270 280 290 300 310 320
50.1
63.1
76.1
89.1
102.1
114.1
130.1
144.1
158.1
172.1
185.1
199.2
213.2
228.2
240.1251.1
270.1281.1
296.1
314.1
O
H
N
N
H
O
M. W.: 228.25

MS of 4b

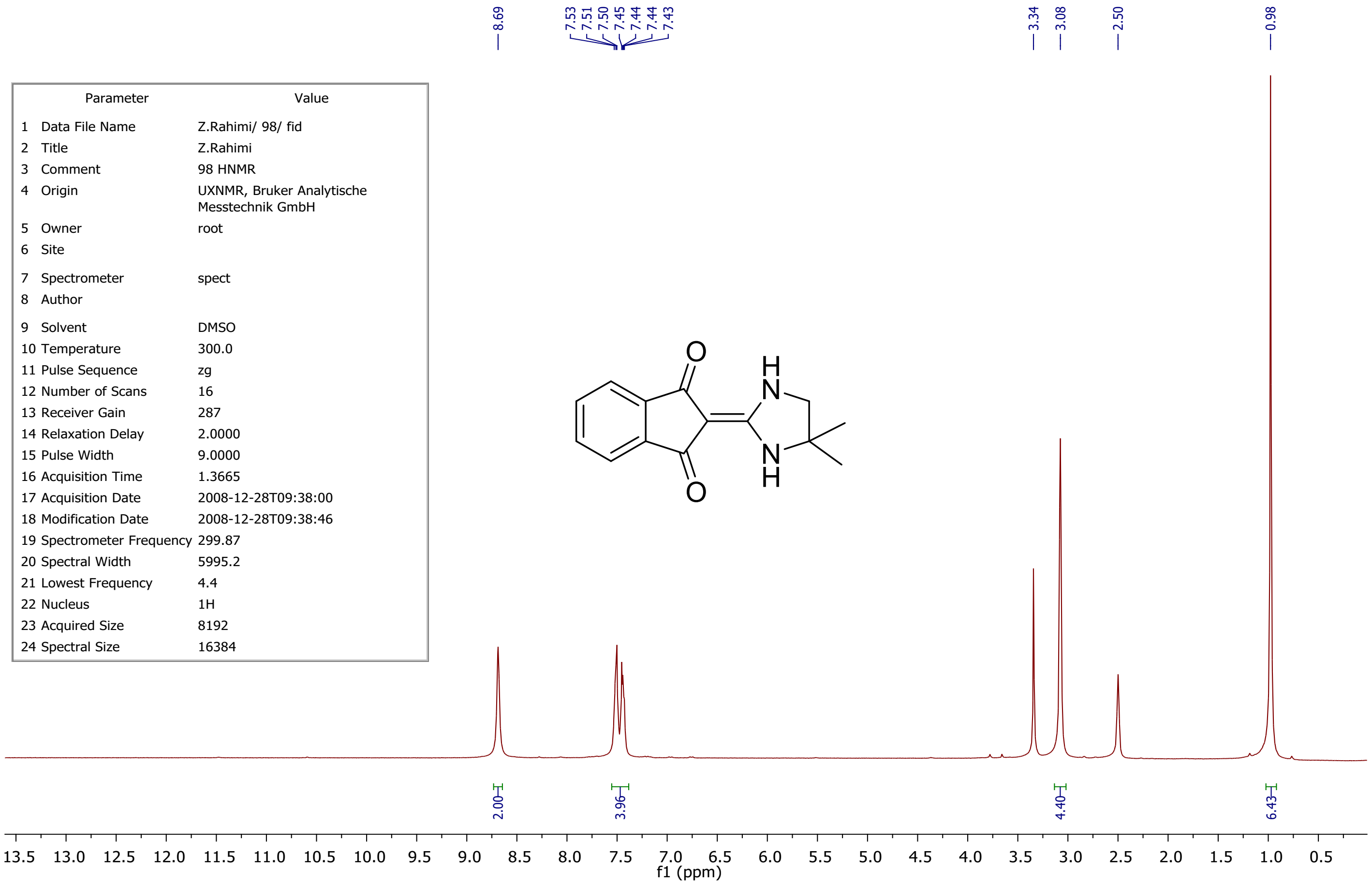
8.69
7.53
7.51
7.50
7.45
7.44
7.44
7.43
3.34
3.08
2.50
0.98
Parameter Value
1 Data File Name Z.Rahimi/ 98/ fid
2 Title Z.Rahimi
3 Comment 98 HNMR
4 Origin UXNMR, Bruker Analytische Messtechnik GmbH
5 Owner root
6 Site
7 Spectrometer spect
8 Author
9 Solvent DMSO
10 Temperature 300.0
11 Pulse Sequence zg
12 Number of Scans 16
13 Receiver Gain 287
14 Relaxation Delay 2.0000
15 Pulse Width 9.0000
16 Acquisition Time 1.3665
17 Acquisition Date 2008-12-28T09:38:00
18 Modification Date 2008-12-28T09:38:46
19 Spectrometer Frequency 299.87
20 Spectral Width 5995.2
21 Lowest Frequency 4.4
22 Nucleus 1H
23 Acquired Size 8192
24 Spectral Size 16384
O
H
N
N
H
O
2.00
3.96
4.40
6.43
13.5 13.0 12.5 12.0 11.5 11.0 10.5 10.0 9.5 9.0 8.5 8.0 7.5 7.0 6.5 6.0 5.5 5.0 4.5 4.0 3.5 3.0 2.5 2.0 1.5 1.0 0.5
f1 (ppm)

^{1}H NMR of 4c

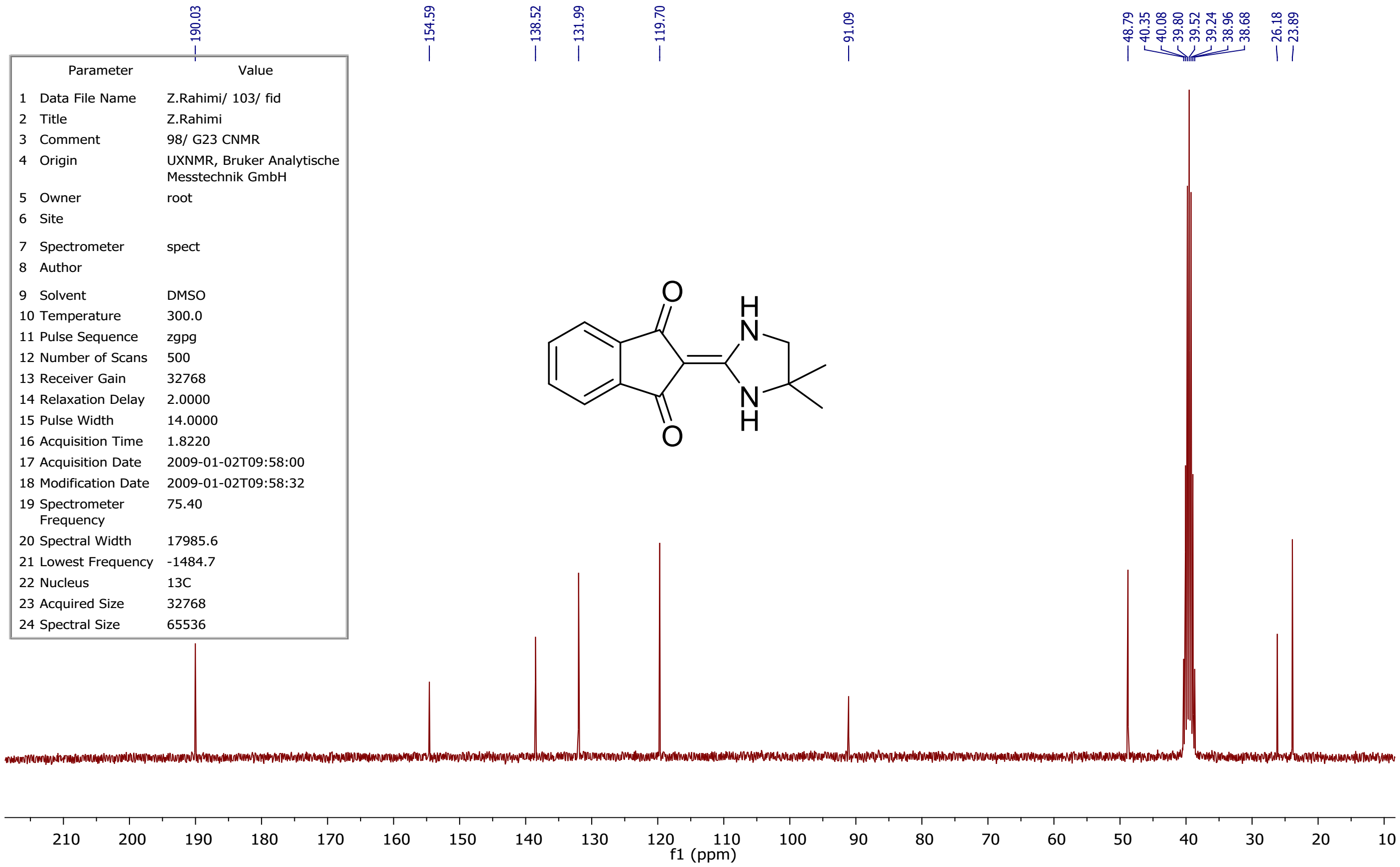

	Parameter	Value
1	Data File Name	Z.Rahimi/ 103/ fid
2	Title	Z.Rahimi
3	Comment	98/ G23 CNMR
4	Origin	UXNMR, Bruker Analytische Messtechnik GmbH
5	Owner	root
6	Site	
7	Spectrometer	spect
8	Author	
9	Solvent	DMSO
10	Temperature	300.0
11	Pulse Sequence	zgpg
12	Number of Scans	500
13	Receiver Gain	32768
14	Relaxation Delay	2.0000
15	Pulse Width	14.0000
16	Acquisition Time	1.8220
17	Acquisition Date	2009-01-02T09:58:00
18	Modification Date	2009-01-02T09:58:32
19	Spectrometer Frequency	75.40
20	Spectral Width	17985.6
21	Lowest Frequency	-1484.7
22	Nucleus	13C
23	Acquired Size	32768
24	Spectral Size	65536

^{13}C NMR of 4c

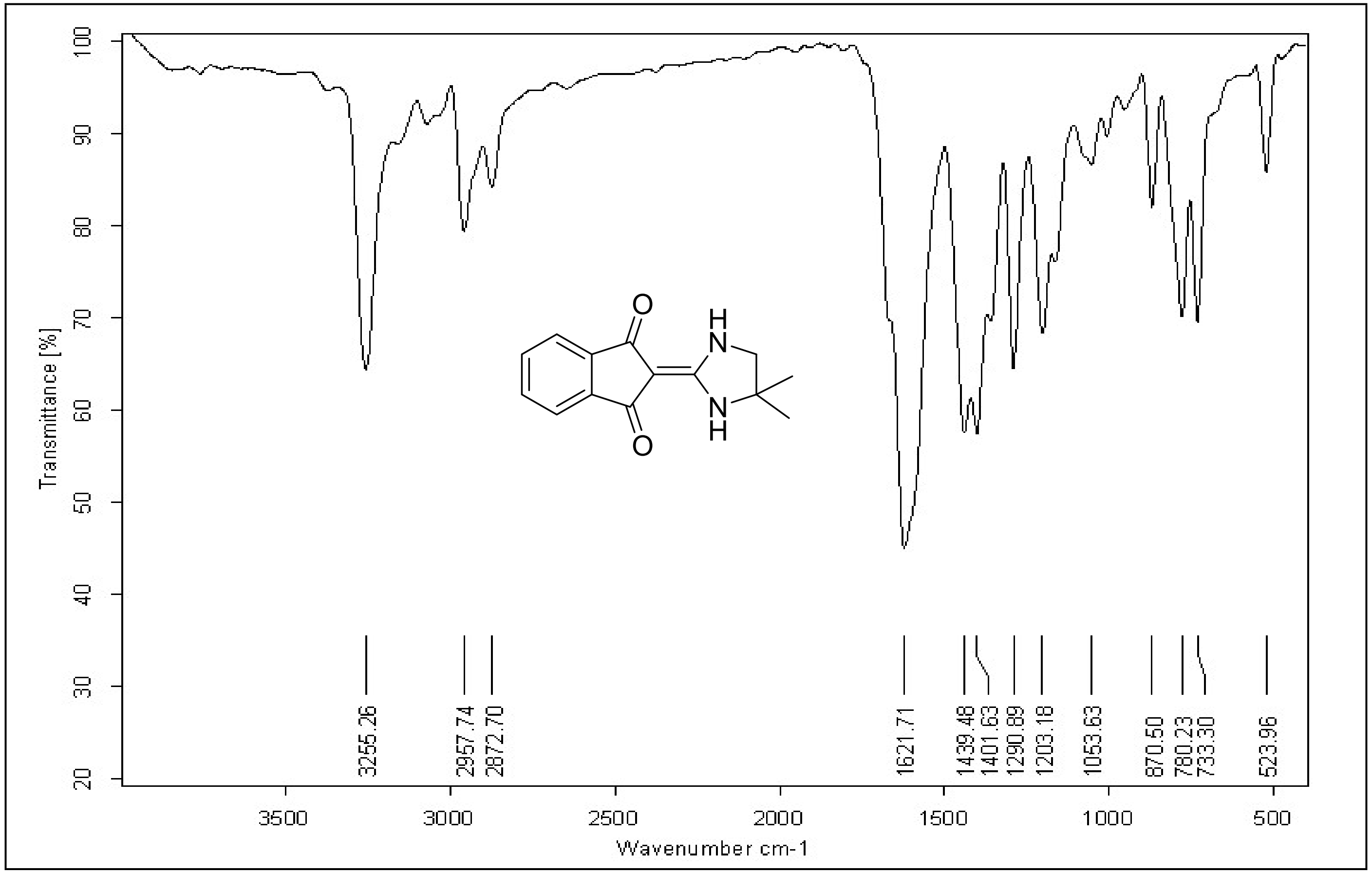
Transmittance [%]
Wavenumber cm-1
3255.26
2957.74
2872.70
1621.71
1439.48
1401.63
1290.89
1203.18
1053.63
870.50
780.23
733.30
523.96
O
O
H
N
N
H

IR of 4c

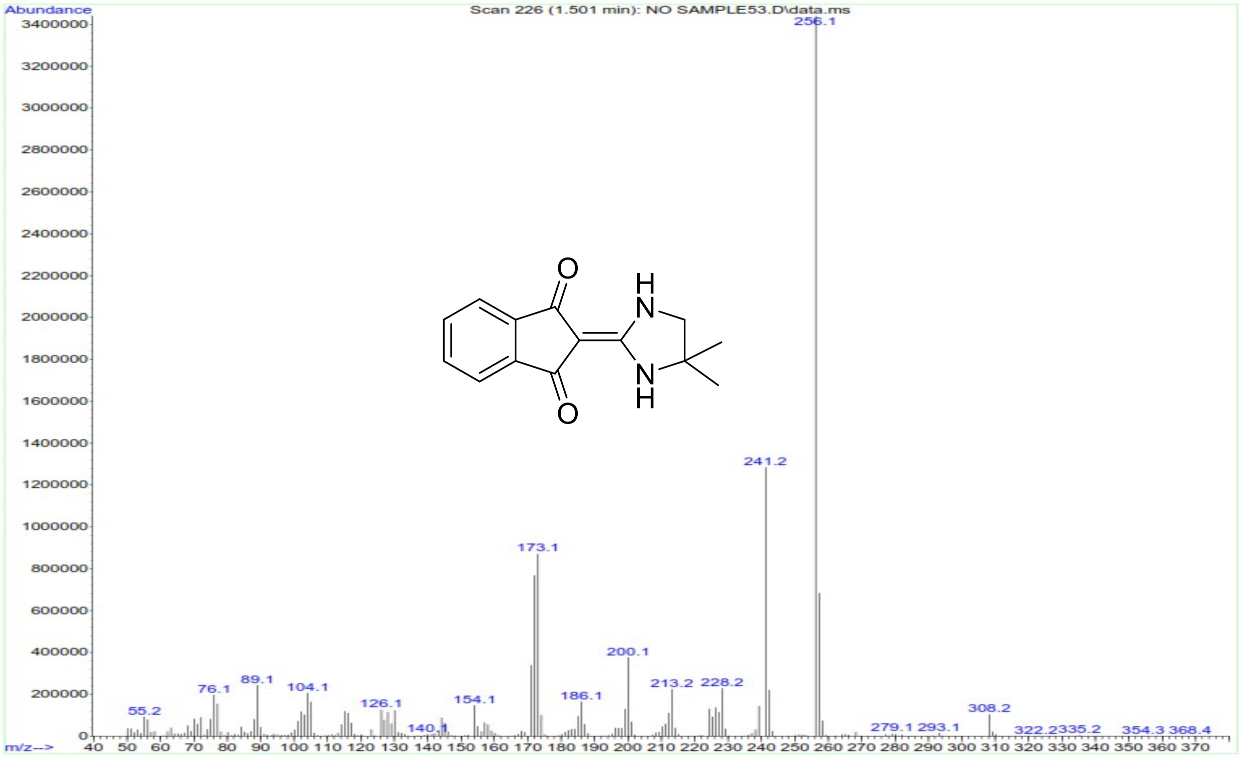

Abundance
Scan 226 (1.501 min): NO SAMPLE53.D\data.ms
256.1
241.2
173.1
200.1
186.1
213.2
228.2
154.1
126.1
140.1
104.1
89.1
76.1
55.2
279.1
293.1
308.2
322.2
335.2
354.3
368.4
m/z-->
O
H
N
N
H
O

MS of 4c

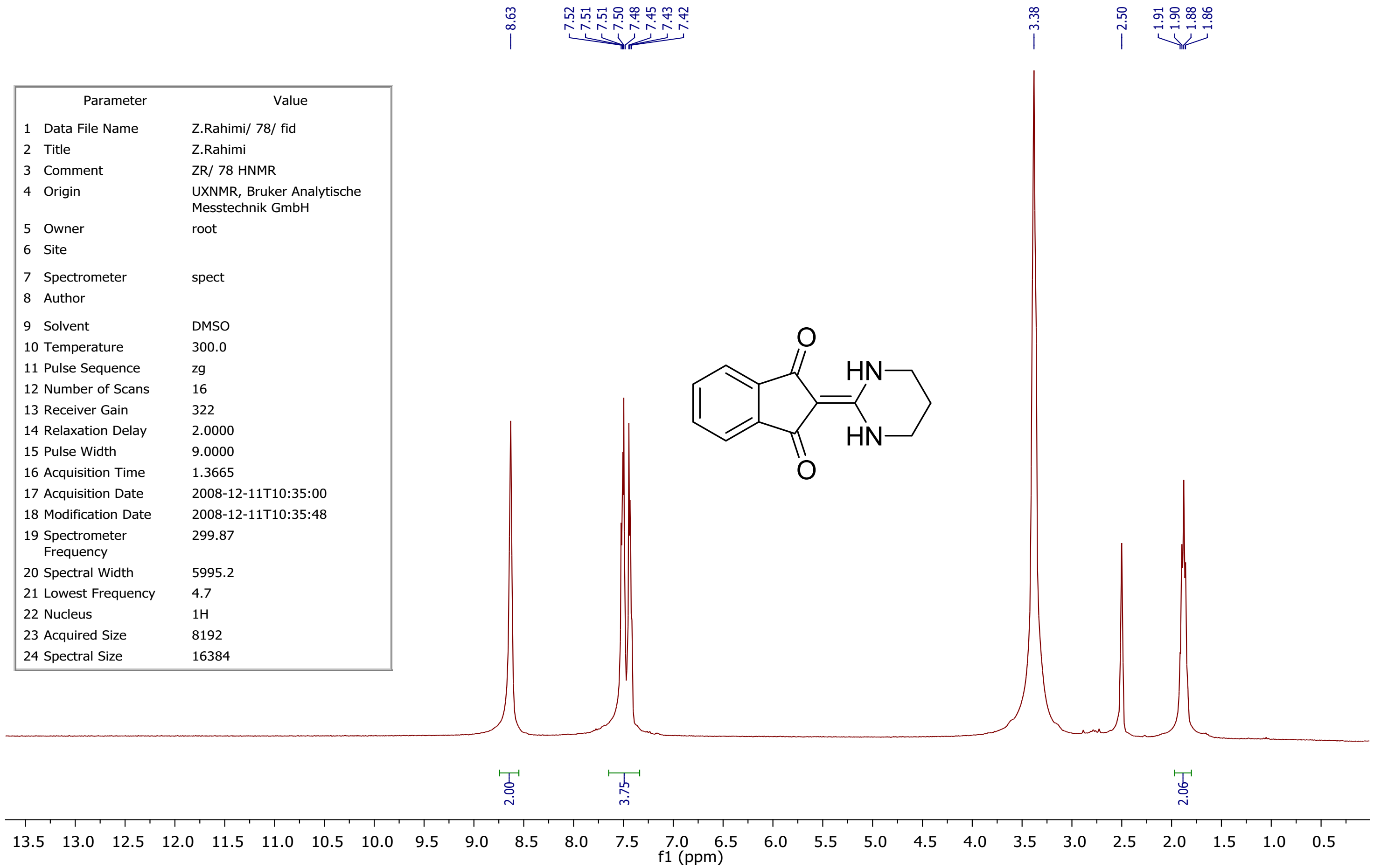
8.63
7.52
7.51
7.51
7.50
7.48
7.45
7.43
7.42
3.38
2.50
1.91
1.90
1.88
1.86
Parameter Value
1 Data File Name Z.Rahimi/ 78/ fid
2 Title Z.Rahimi
3 Comment ZR/ 78 HNMR
4 Origin UXNMR, Bruker Analytische Messtechnik GmbH
5 Owner root
6 Site
7 Spectrometer spect
8 Author
9 Solvent DMSO
10 Temperature 300.0
11 Pulse Sequence zg
12 Number of Scans 16
13 Receiver Gain 322
14 Relaxation Delay 2.0000
15 Pulse Width 9.0000
16 Acquisition Time 1.3665
17 Acquisition Date 2008-12-11T10:35:00
18 Modification Date 2008-12-11T10:35:48
19 Spectrometer Frequency 299.87
20 Spectral Width 5995.2
21 Lowest Frequency 4.7
22 Nucleus 1H
23 Acquired Size 8192
24 Spectral Size 16384
O
HN
HN
O
2.00
3.75
2.06
13.5 13.0 12.5 12.0 11.5 11.0 10.5 10.0 9.5 9.0 8.5 8.0 7.5 7.0 6.5 6.0 5.5 5.0 4.5 4.0 3.5 3.0 2.5 2.0 1.5 1.0 0.5
f1 (ppm)

^{1}H NMR of 4d

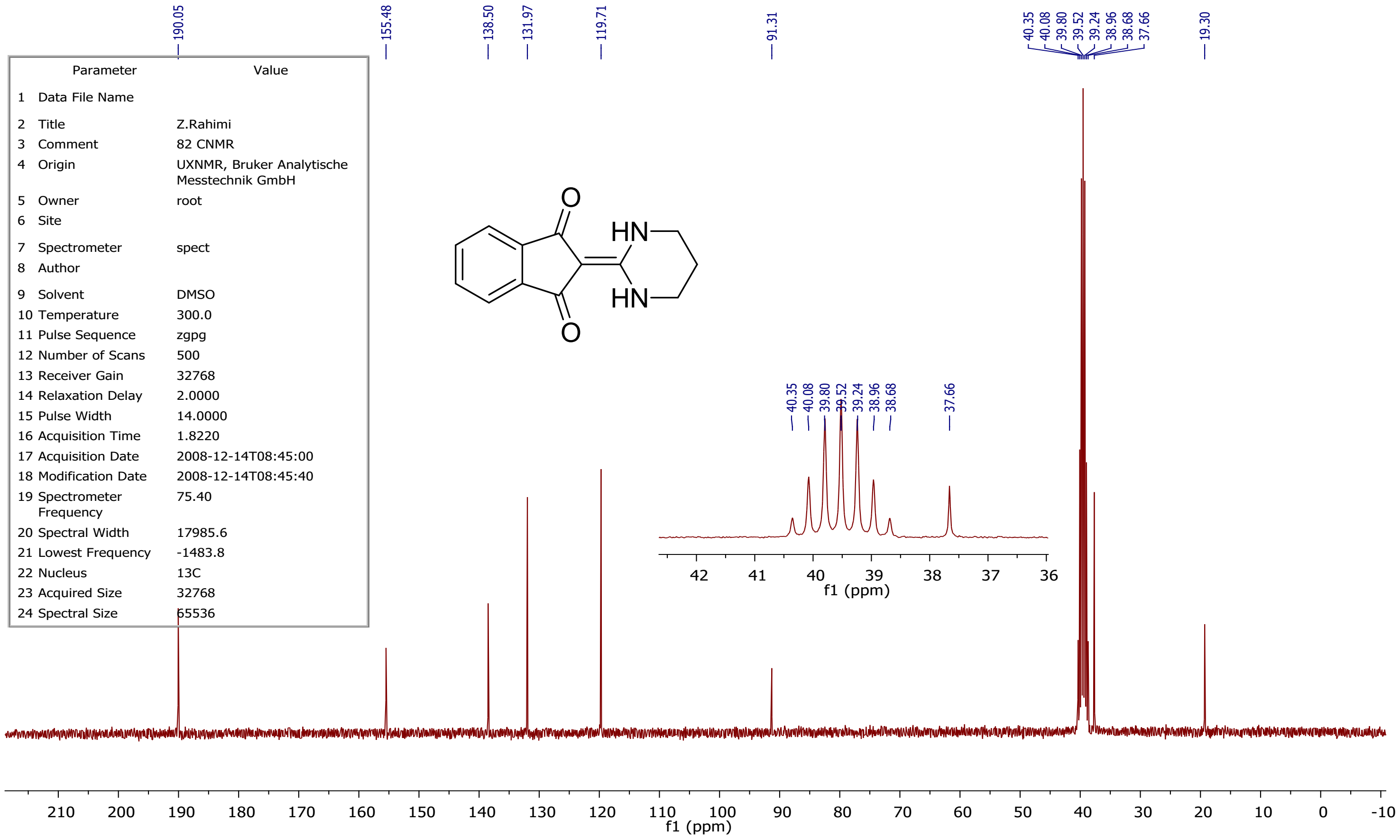
190.05
155.48
138.50
131.97
119.71
91.31
40.35
40.08
39.80
39.52
39.24
38.96
38.68
37.66
19.30
Parameter Value
1 Data File Name
2 Title Z.Rahimi
3 Comment 82 CNMR
4 Origin UXNMR, Bruker Analytische Messtechnik GmbH
5 Owner root
6 Site
7 Spectrometer spect
8 Author
9 Solvent DMSO
10 Temperature 300.0
11 Pulse Sequence zgpg
12 Number of Scans 500
13 Receiver Gain 32768
14 Relaxation Delay 2.0000
15 Pulse Width 14.0000
16 Acquisition Time 1.8220
17 Acquisition Date 2008-12-14T08:45:00
18 Modification Date 2008-12-14T08:45:40
19 Spectrometer Frequency 75.40
20 Spectral Width 17985.6
21 Lowest Frequency -1483.8
22 Nucleus 13C
23 Acquired Size 32768
24 Spectral Size 65536
O
HN
HN
O
f1 (ppm)
f1 (ppm)

^{13}C NMR of 4d

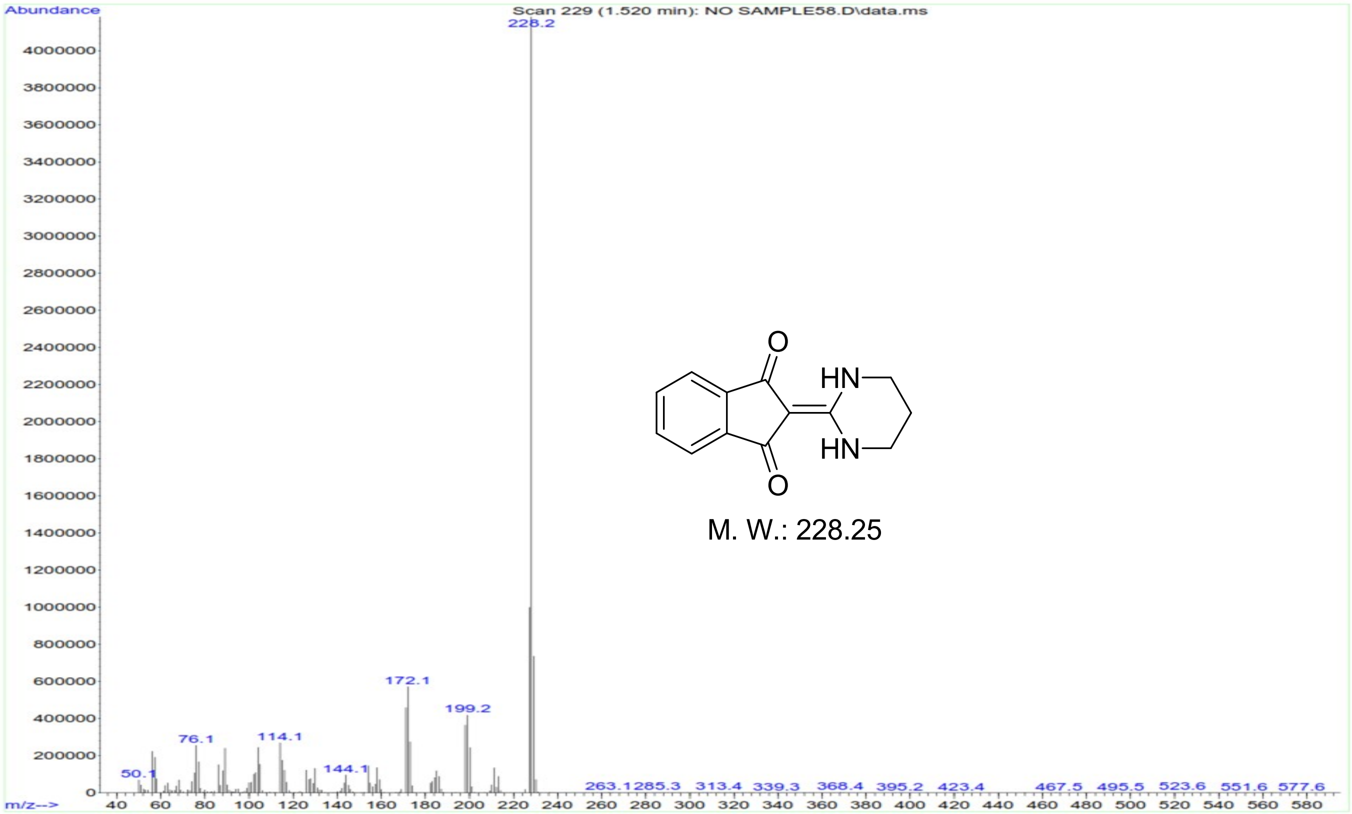
Abundance
Scan 229 (1.520 min): NO SAMPLE58.D\data.ms
228.2
172.1
199.2
114.1
76.1
144.1
50.1
263.1
285.3
313.4
339.3
368.4
395.2
423.4
467.5
495.5
523.6
551.6
577.6
m/z-->
O
HN
HN
O
M. W.: 228.25

MS of 4d

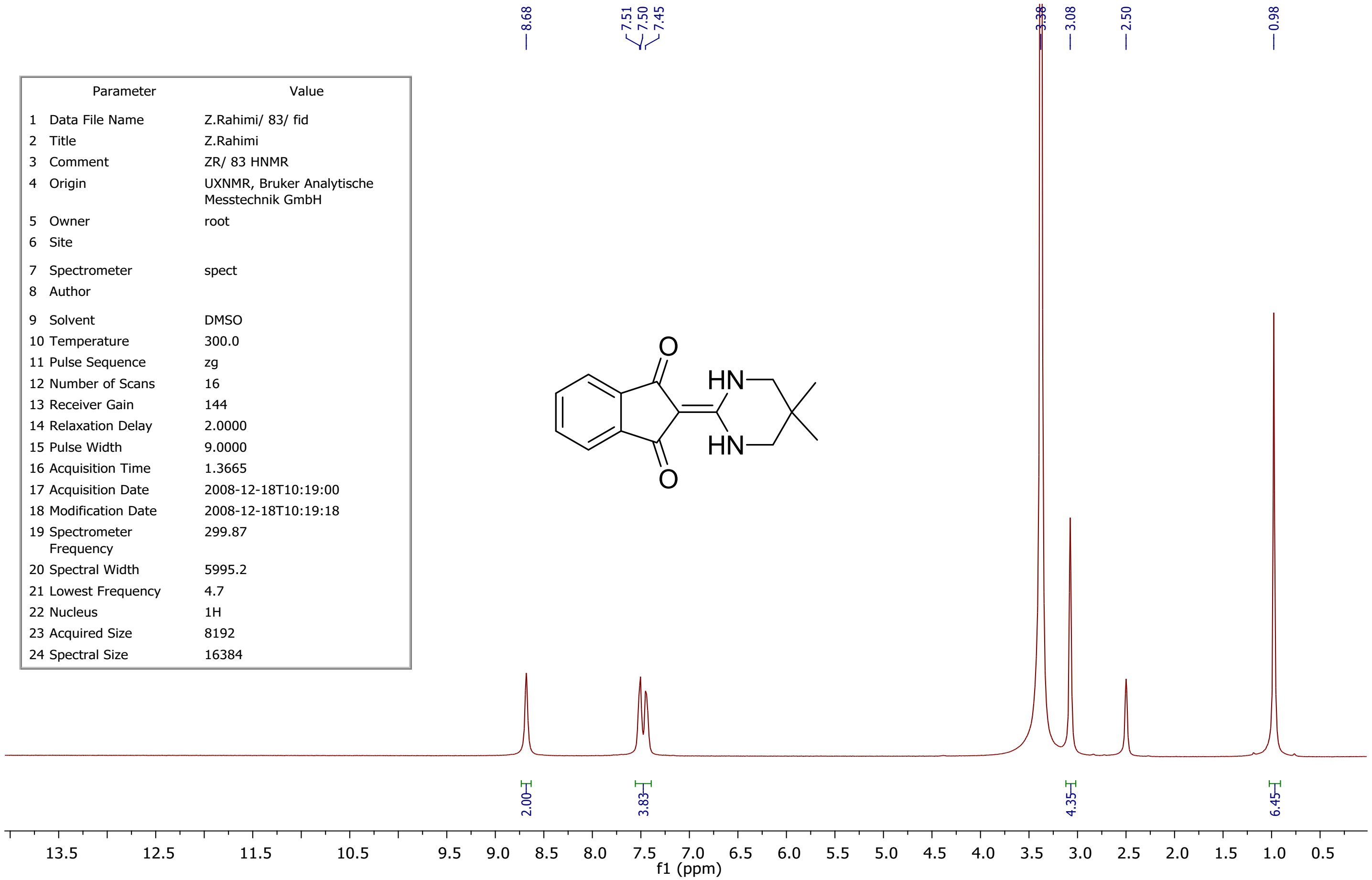

	Parameter	Value
1	Data File Name	Z.Rahimi/ 83/ fid
2	Title	Z.Rahimi
3	Comment	ZR/ 83 HNMR
4	Origin	UXNMR, Bruker Analytische Messtechnik GmbH
5	Owner	root
6	Site	
7	Spectrometer	spect
8	Author	
9	Solvent	DMSO
10	Temperature	300.0
11	Pulse Sequence	zg
12	Number of Scans	16
13	Receiver Gain	144
14	Relaxation Delay	2.0000
15	Pulse Width	9.0000
16	Acquisition Time	1.3665
17	Acquisition Date	2008-12-18T10:19:00
18	Modification Date	2008-12-18T10:19:18
19	Spectrometer Frequency	299.87
20	Spectral Width	5995.2
21	Lowest Frequency	4.7
22	Nucleus	1H
23	Acquired Size	8192
24	Spectral Size	16384

^{1}H NMR of 4e

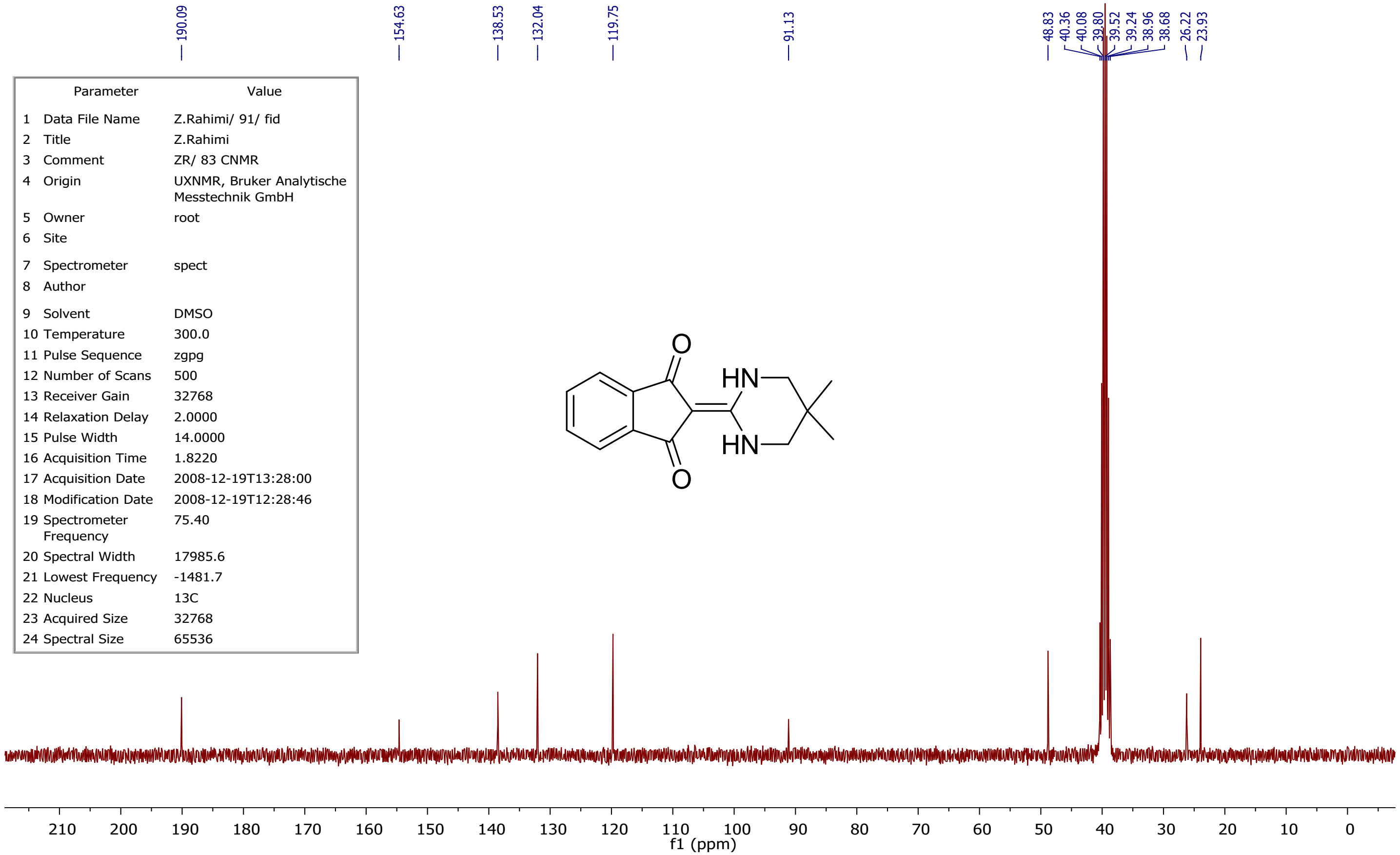

	Parameter	Value
1	Data File Name	Z.Rahimi/ 91/ fid
2	Title	Z.Rahimi
3	Comment	ZR/ 83 CNMR
4	Origin	UXNMR, Bruker Analytische Messtechnik GmbH
5	Owner	root
6	Site	
7	Spectrometer	spect
8	Author	
9	Solvent	DMSO
10	Temperature	300.0
11	Pulse Sequence	zgpg
12	Number of Scans	500
13	Receiver Gain	32768
14	Relaxation Delay	2.0000
15	Pulse Width	14.0000
16	Acquisition Time	1.8220
17	Acquisition Date	2008-12-19T13:28:00
18	Modification Date	2008-12-19T12:28:46
19	Spectrometer Frequency	75.40
20	Spectral Width	17985.6
21	Lowest Frequency	-1481.7
22	Nucleus	13C
23	Acquired Size	32768
24	Spectral Size	65536

^{13}C NMR of 4e

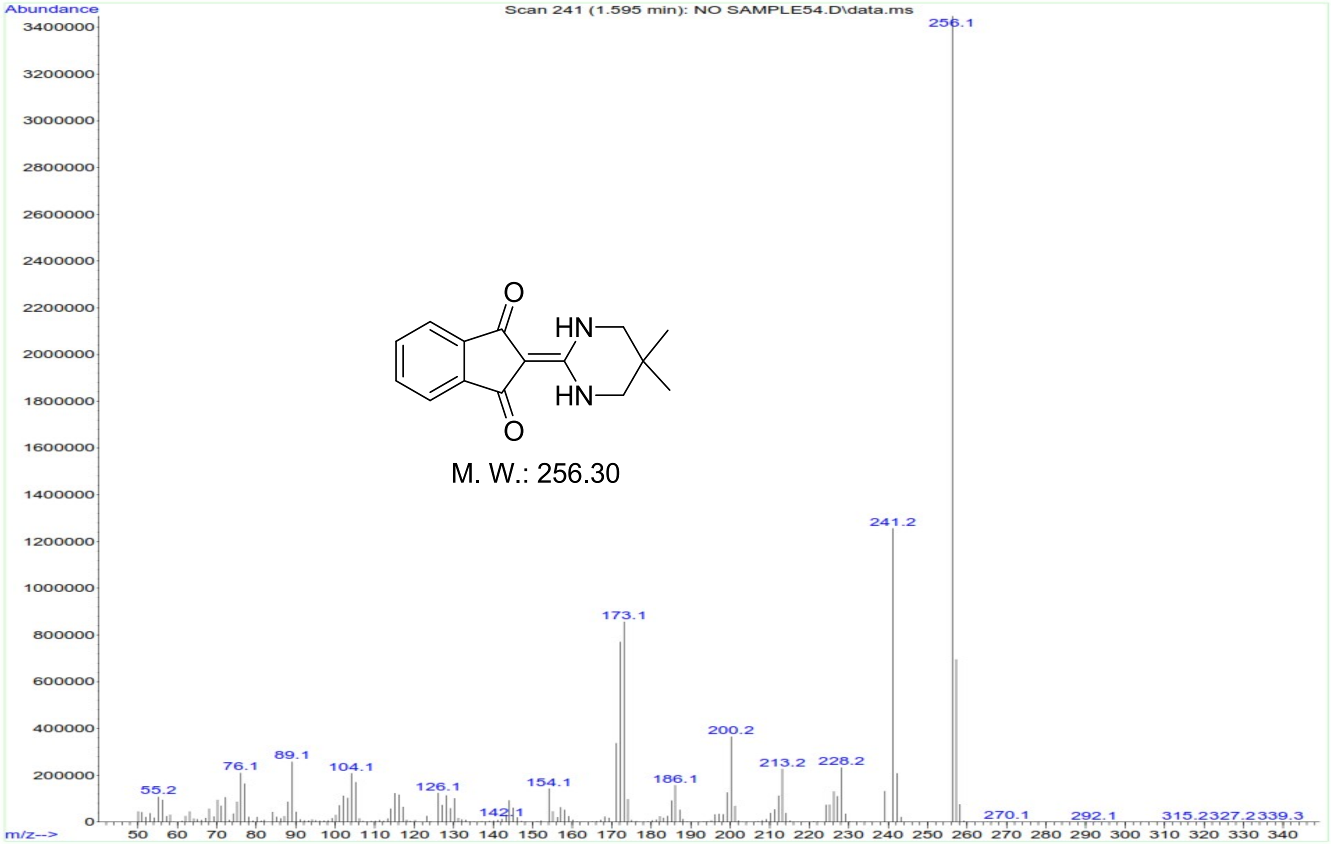
Abundance
Scan 241 (1.595 min): NO SAMPLE54.D\data.ms
3400000
3200000
3000000
2800000
2600000
2400000
2200000
2000000
1800000
1600000
1400000
1200000
1000000
800000
600000
400000
200000
0
m/z-->
50 60 70 80 90 100 110 120 130 140 150 160 170 180 190 200 210 220 230 240 250 260 270 280 290 300 310 320 330 340
55.2
76.1
89.1
104.1
126.1
142.1
154.1
173.1
186.1
200.2
213.2
228.2
241.2
256.1
270.1
292.1
315.2327.2339.3
O
HN
HN
O
M. W.: 256.30

MS of 4e

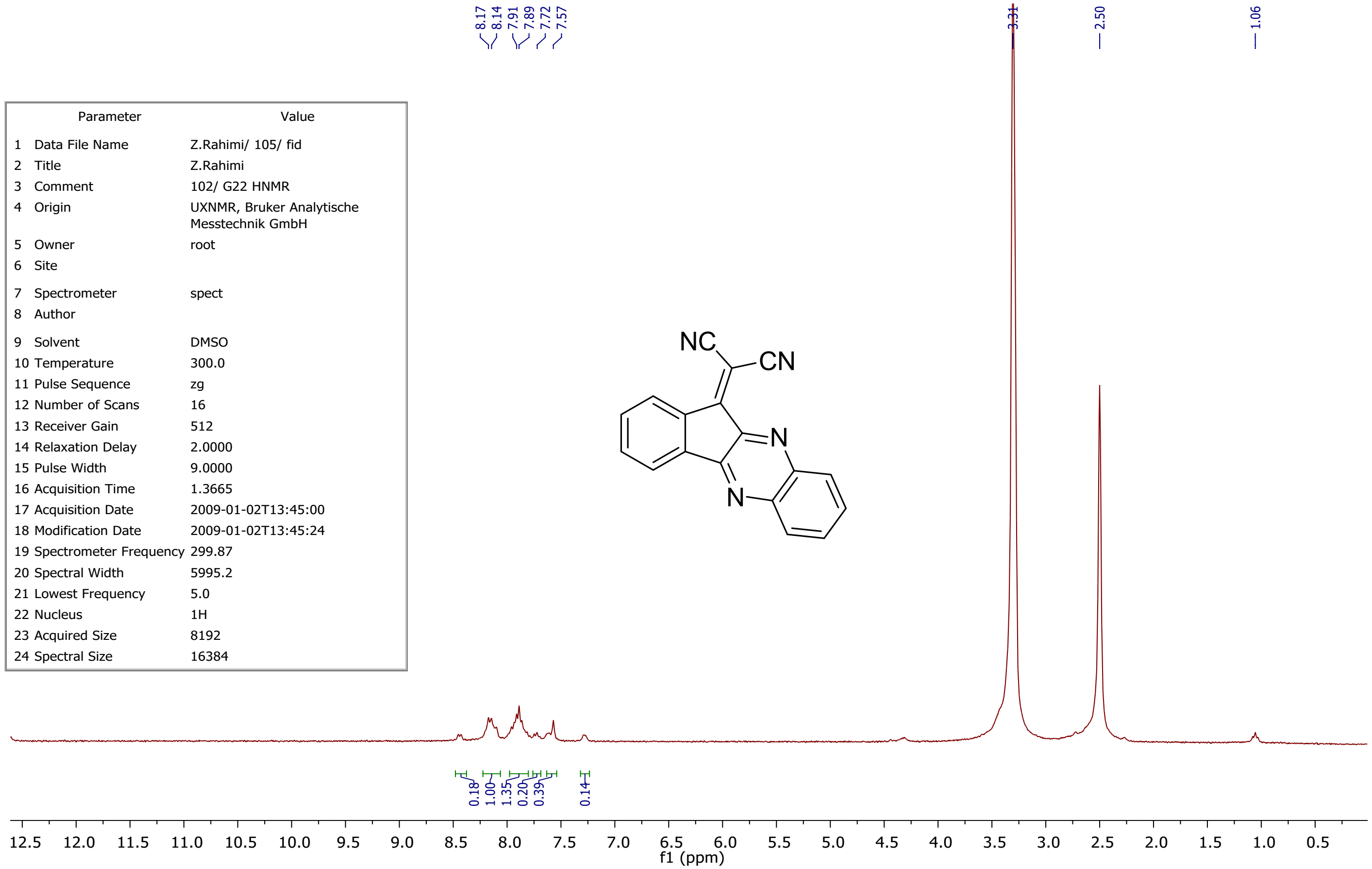

	Parameter	Value
1	Data File Name	Z.Rahimi/ 105/ fid
2	Title	Z.Rahimi
3	Comment	102/ G22 HNMR
4	Origin	UXNMR, Bruker Analytische Messtechnik GmbH
5	Owner	root
6	Site	
7	Spectrometer	spect
8	Author	
9	Solvent	DMSO
10	Temperature	300.0
11	Pulse Sequence	zg
12	Number of Scans	16
13	Receiver Gain	512
14	Relaxation Delay	2.0000
15	Pulse Width	9.0000
16	Acquisition Time	1.3665
17	Acquisition Date	2009-01-02T13:45:00
18	Modification Date	2009-01-02T13:45:24
19	Spectrometer Frequency	299.87
20	Spectral Width	5995.2
21	Lowest Frequency	5.0
22	Nucleus	1H
23	Acquired Size	8192
24	Spectral Size	16384

^{1}H NMR of 6a

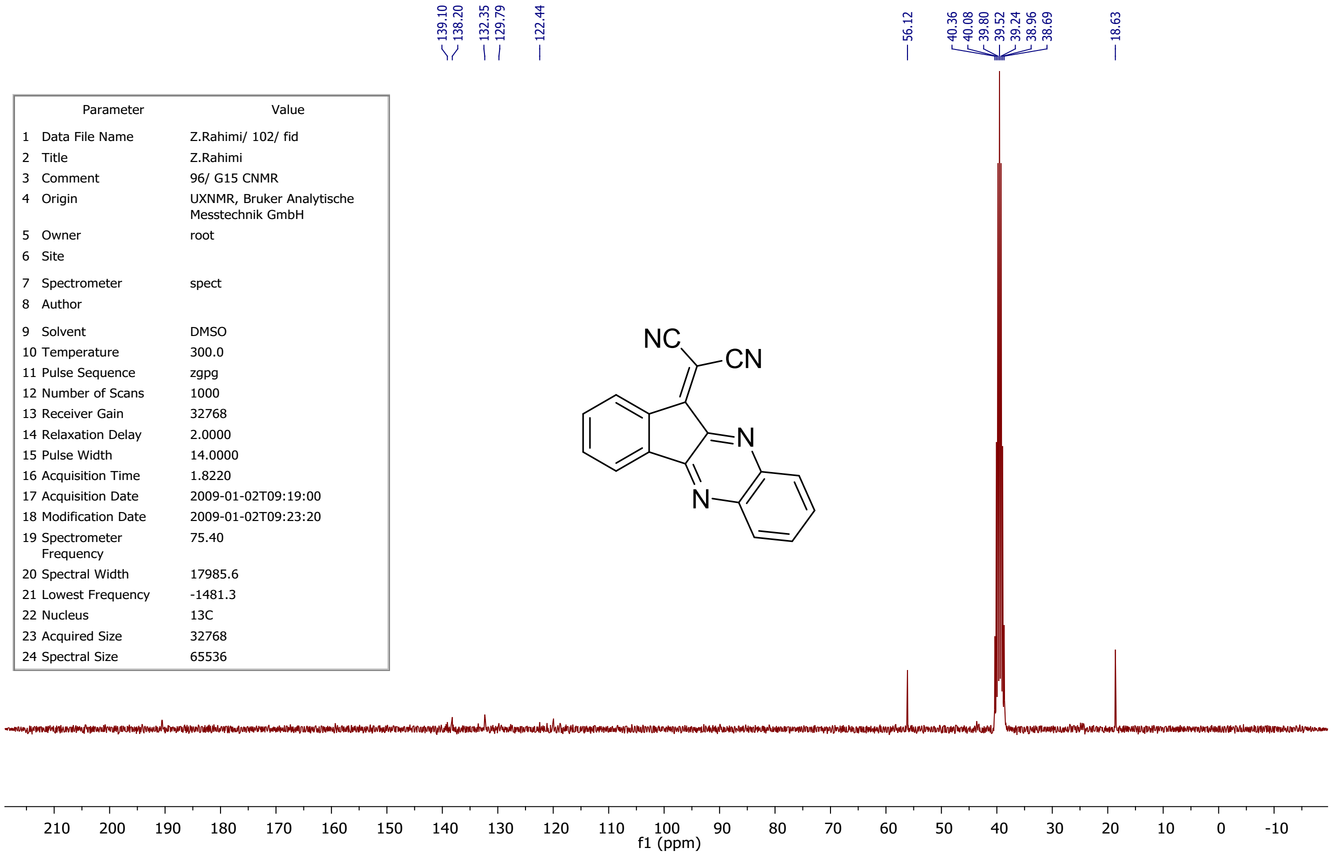

	Parameter	Value
1	Data File Name	Z.Rahimi/ 102/ fid
2	Title	Z.Rahimi
3	Comment	96/ G15 CNMR
4	Origin	UXNMR, Bruker Analytische Messtechnik GmbH
5	Owner	root
6	Site	
7	Spectrometer	spect
8	Author	
9	Solvent	DMSO
10	Temperature	300.0
11	Pulse Sequence	zgpg
12	Number of Scans	1000
13	Receiver Gain	32768
14	Relaxation Delay	2.0000
15	Pulse Width	14.0000
16	Acquisition Time	1.8220
17	Acquisition Date	2009-01-02T09:19:00
18	Modification Date	2009-01-02T09:23:20
19	Spectrometer Frequency	75.40
20	Spectral Width	17985.6
21	Lowest Frequency	-1481.3
22	Nucleus	13C
23	Acquired Size	32768
24	Spectral Size	65536

^{13}C NMR of 6a

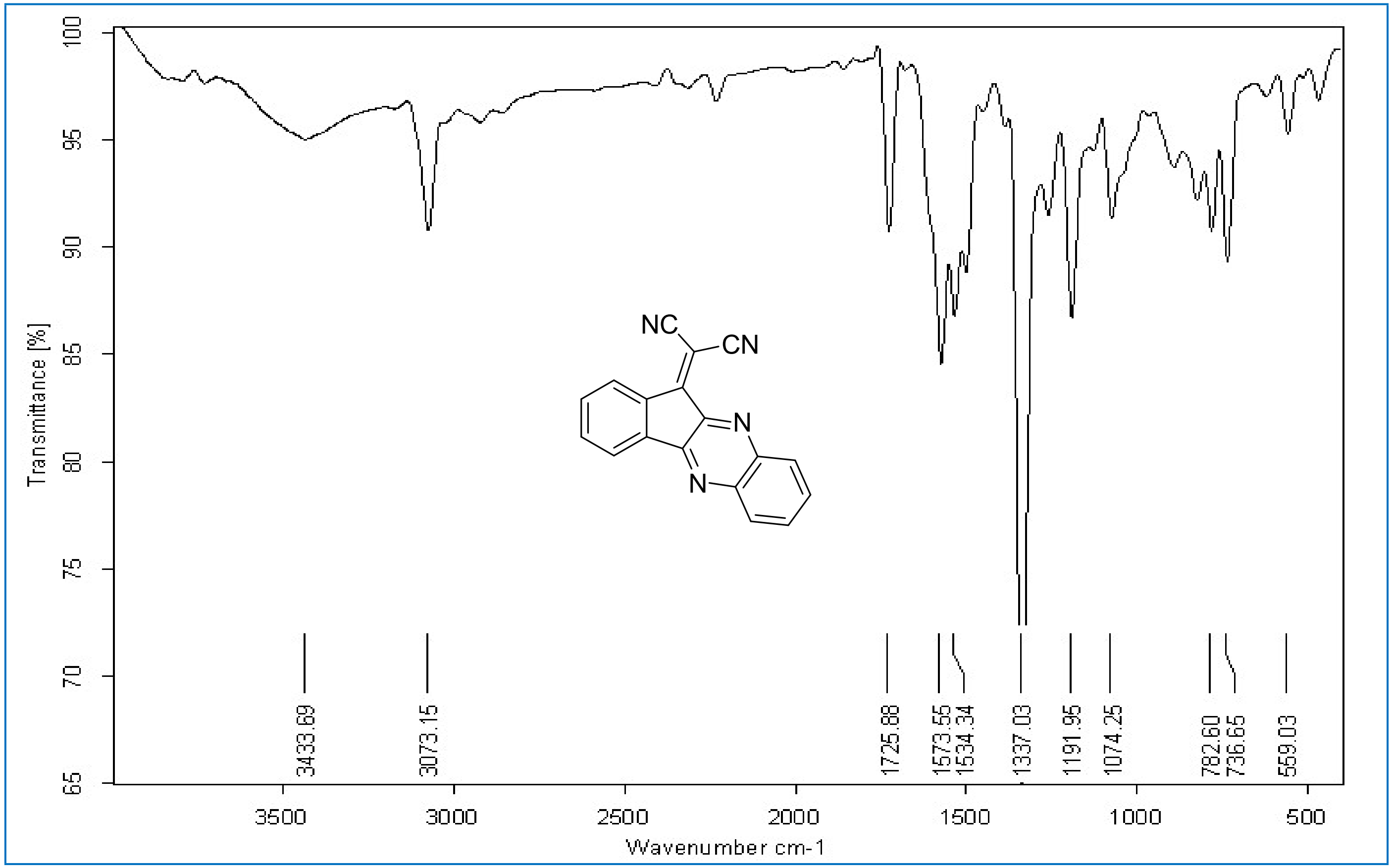
Transmittance [%]
Wavenumber cm-1
100
95
90
85
80
75
70
65
3500
3000
2500
2000
1500
1000
500
3433.69
3073.15
1725.88
1573.55
1534.34
1337.03
1191.95
1074.25
782.60
736.65
559.03
NC
CN
N
N

IR of 6a

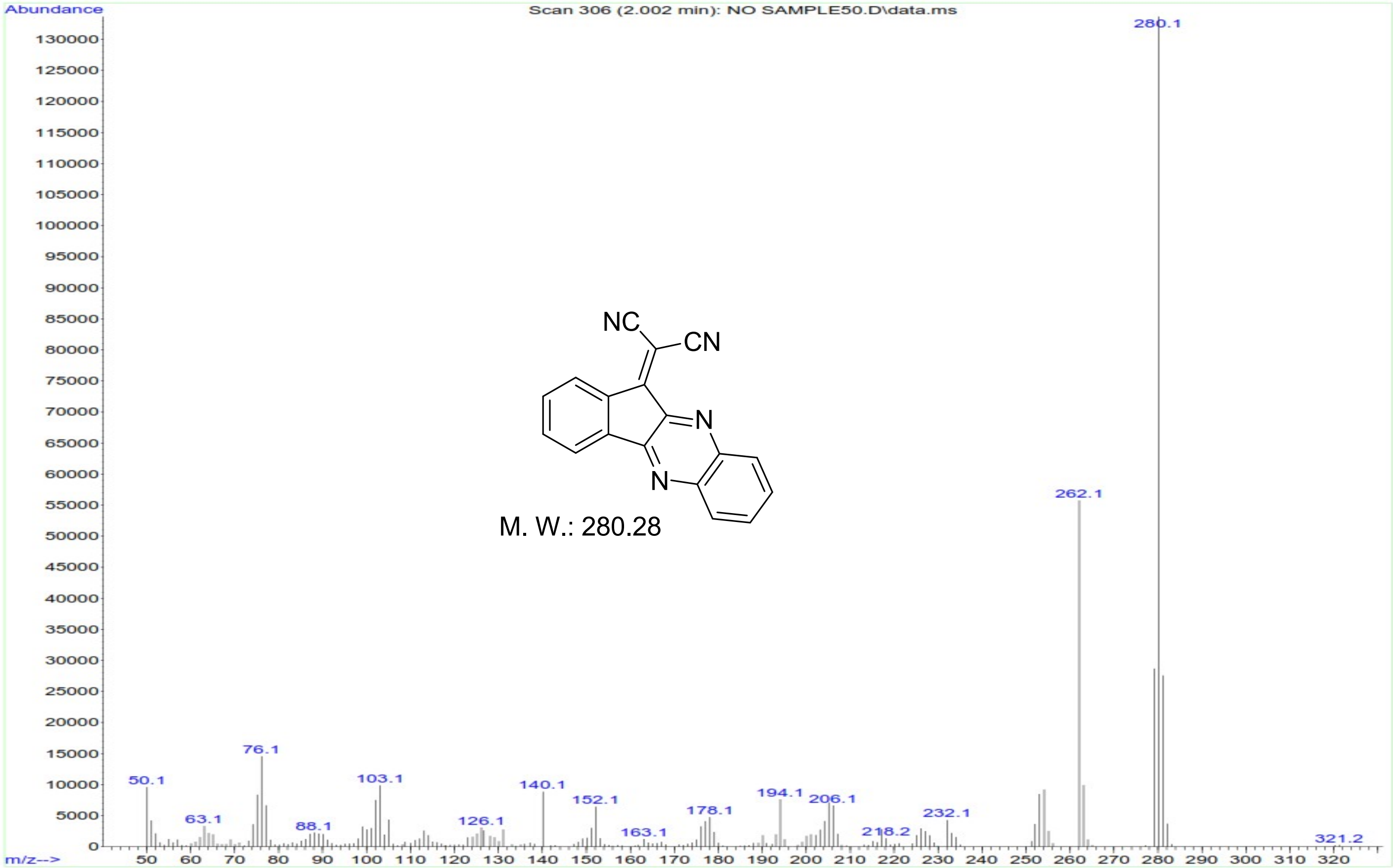
Abundance
Scan 306 (2.002 min): NO SAMPLE50.D\data.ms
280.1
262.1
76.1
50.1
63.1
88.1
103.1
126.1
140.1
152.1
163.1
178.1
194.1
206.1
218.2
232.1
321.2
NC
CN
N
N
M. W.: 280.28
m/z-->

MS of 6a

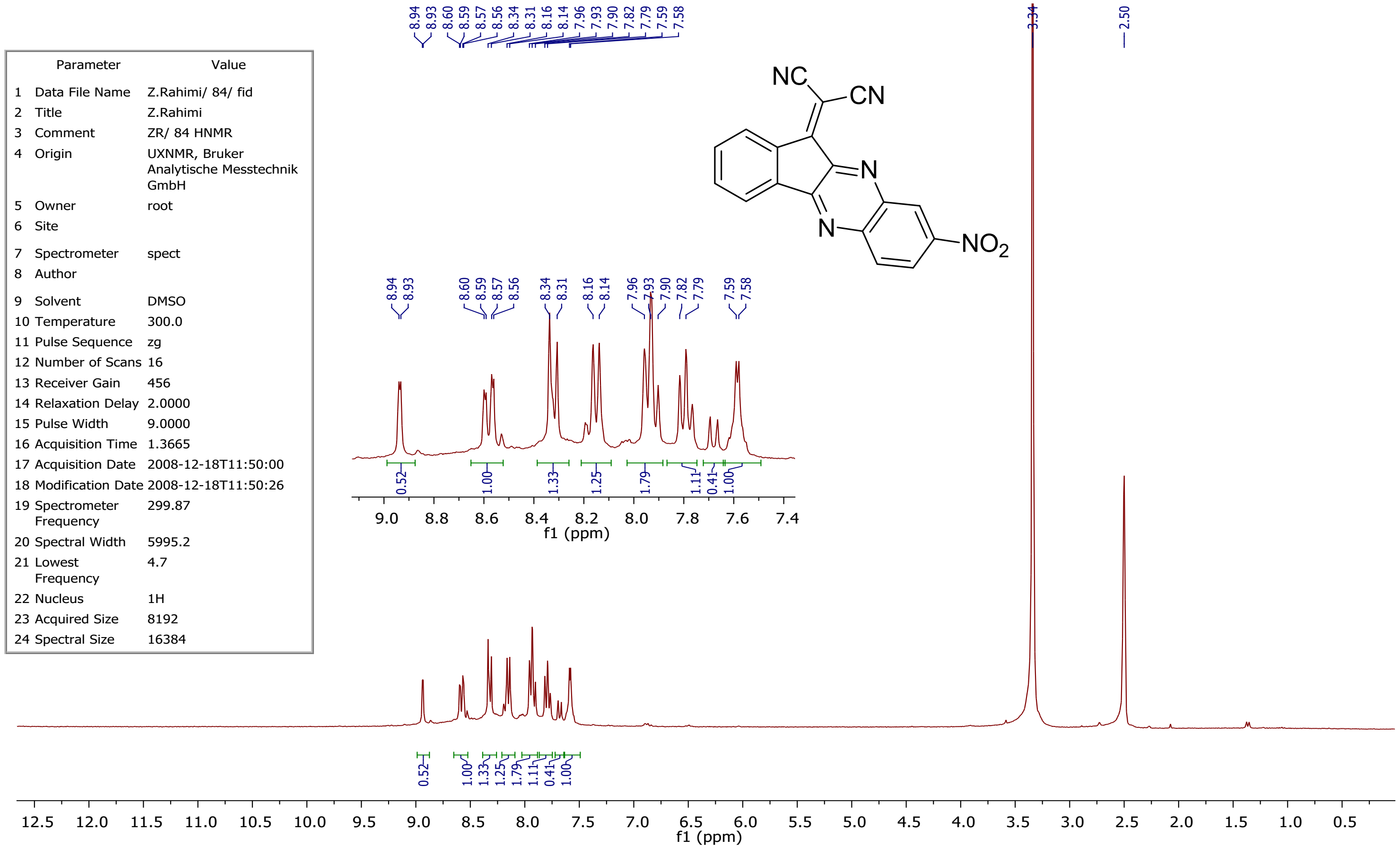
Parameter Value
1 Data File Name Z.Rahimi/ 84/ fid
2 Title Z.Rahimi
3 Comment ZR/ 84 HNMR
4 Origin UXNMR, Bruker Analytische Messtechnik GmbH
5 Owner root
6 Site
7 Spectrometer spect
8 Author
9 Solvent DMSO
10 Temperature 300.0
11 Pulse Sequence zg
12 Number of Scans 16
13 Receiver Gain 456
14 Relaxation Delay 2.0000
15 Pulse Width 9.0000
16 Acquisition Time 1.3665
17 Acquisition Date 2008-12-18T11:50:00
18 Modification Date 2008-12-18T11:50:26
19 Spectrometer Frequency 299.87
20 Spectral Width 5995.2
21 Lowest Frequency 4.7
22 Nucleus 1H
23 Acquired Size 8192
24 Spectral Size 16384
NC
CN
N
N
NO2
8.94 8.93 8.60 8.59 8.57 8.56 8.34 8.31 8.16 8.14 7.96 7.93 7.90 7.82 7.79 7.59 7.58
3.34
2.50
0.52 1.00 1.33 1.25 1.79 1.11 0.41 1.00
f1 (ppm)

^{1}H NMR of 6b

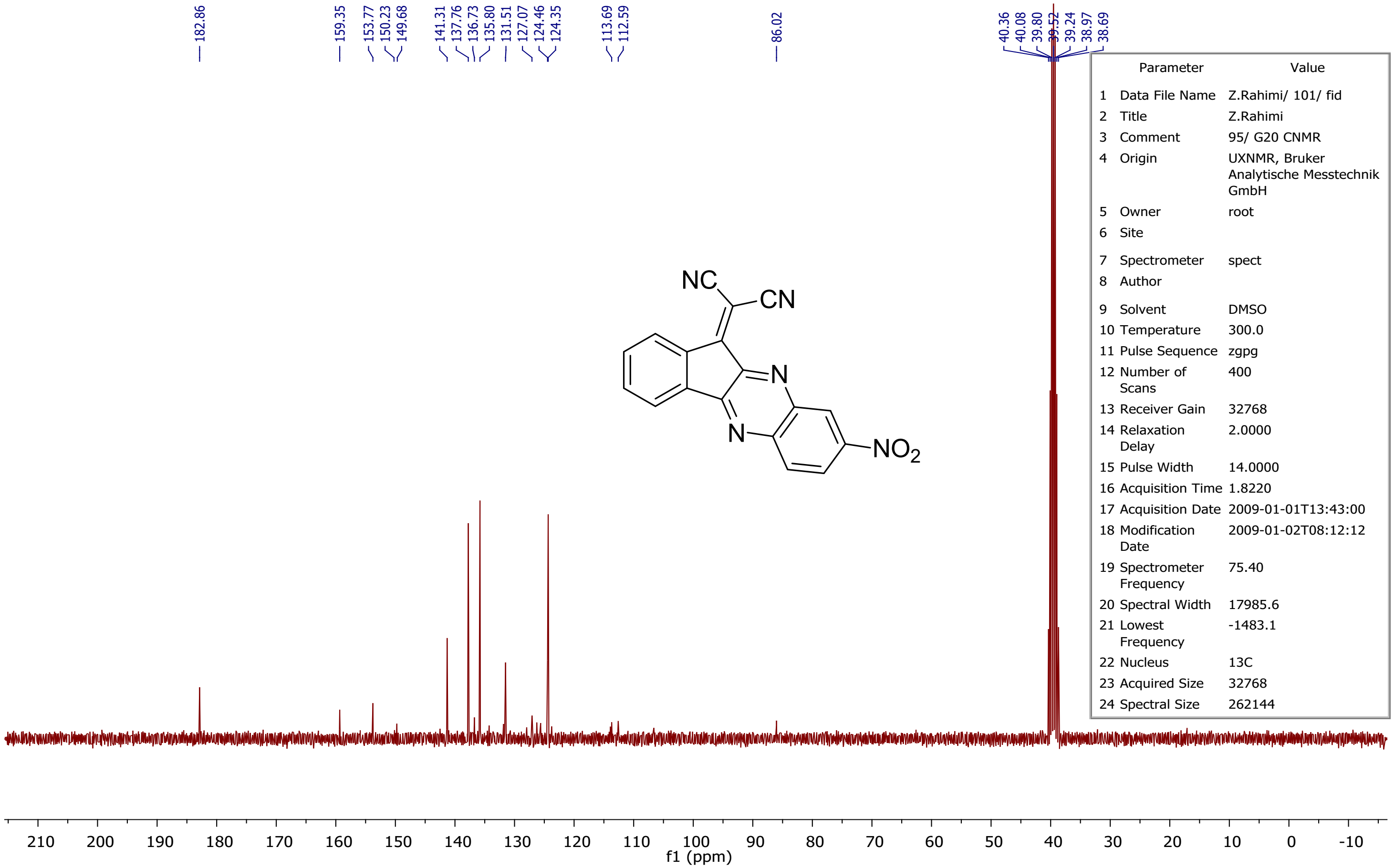

	Parameter	Value
1	Data File Name	Z.Rahimi/ 101/ fid
2	Title	Z.Rahimi
3	Comment	95/ G20 CNMR
4	Origin	UXNMR, Bruker Analytische Messtechnik GmbH
5	Owner	root
6	Site	
7	Spectrometer	spect
8	Author	
9	Solvent	DMSO
10	Temperature	300.0
11	Pulse Sequence	zgpg
12	Number of Scans	400
13	Receiver Gain	32768
14	Relaxation Delay	2.0000
15	Pulse Width	14.0000
16	Acquisition Time	1.8220
17	Acquisition Date	2009-01-01T13:43:00
18	Modification Date	2009-01-02T08:12:12
19	Spectrometer Frequency	75.40
20	Spectral Width	17985.6
21	Lowest Frequency	-1483.1
22	Nucleus	13C
23	Acquired Size	32768
24	Spectral Size	262144

^{13}C NMR of 6b

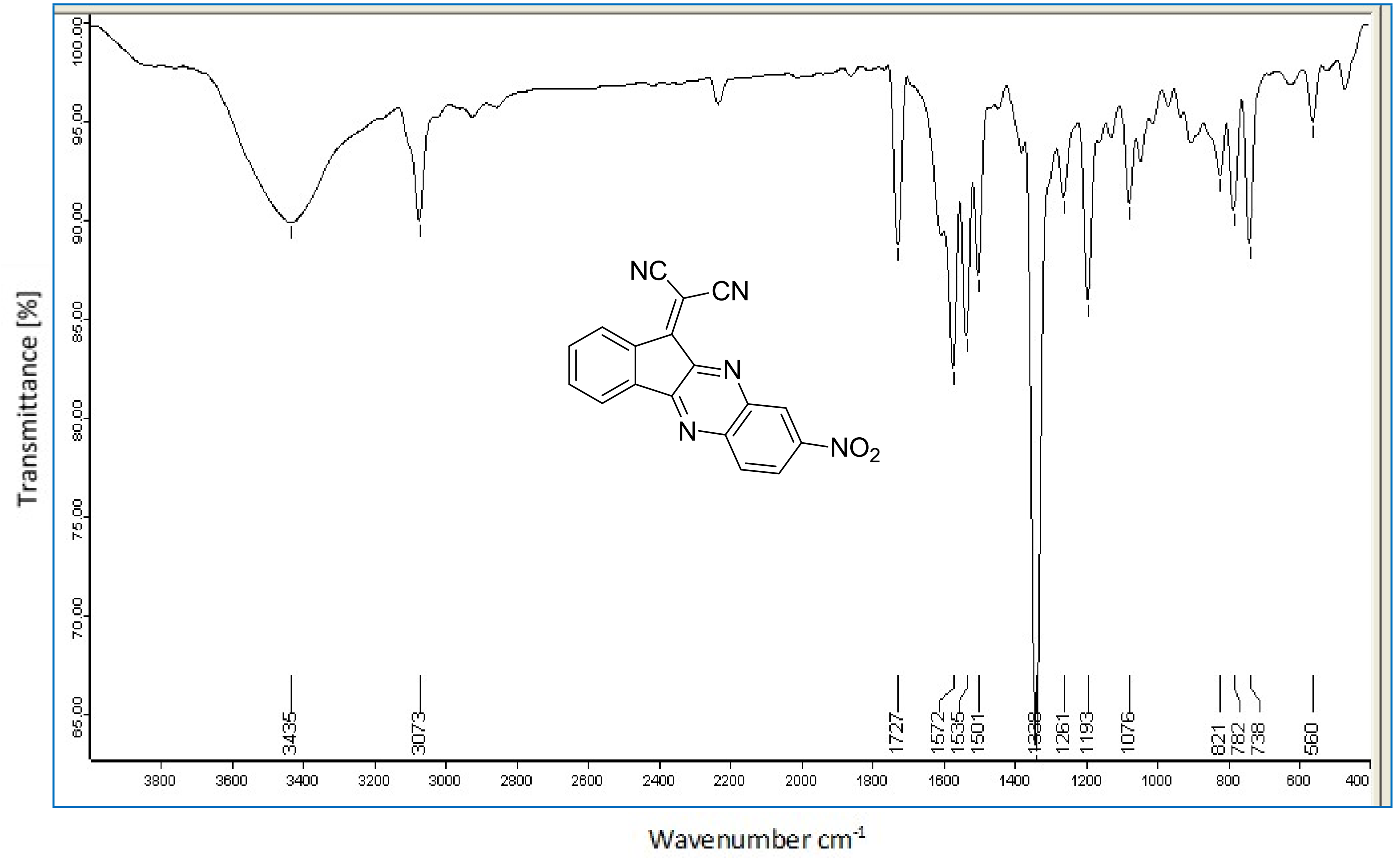

Transmittance [%]
100.00
95.00
90.00
85.00
80.00
75.00
70.00
65.00
3435
3073
1727
1572
1535
1501
1338
1261
1193
1076
821
782
738
560
3800
3600
3400
3200
3000
2800
2600
2400
2200
2000
1800
1600
1400
1200
1000
800
600
400
NC
CN
N
N
NO2
Wavenumber cm-1

IR of 6b

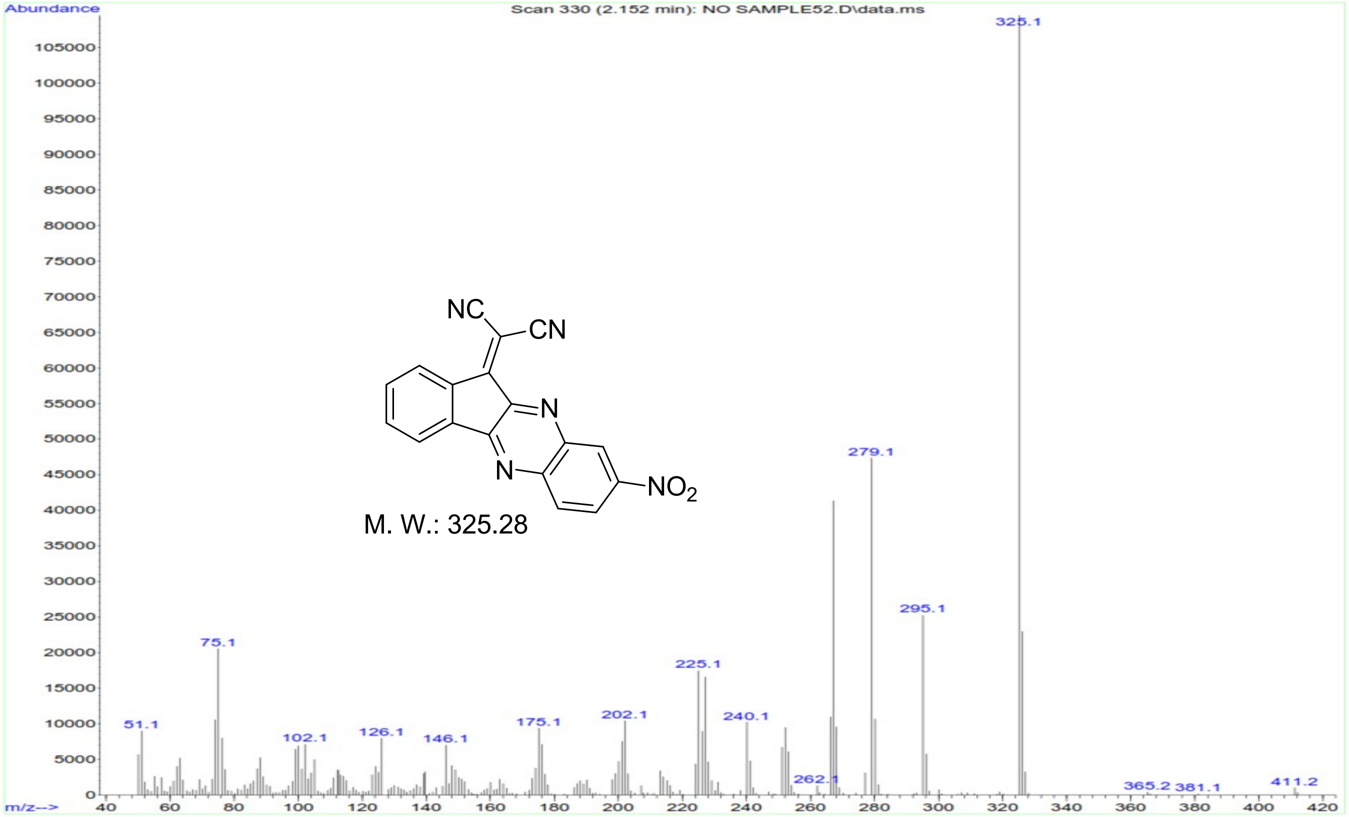
Abundance
Scan 330 (2.152 min): NO SAMPLE52.D\data.ms
325.1
279.1
295.1
225.1
240.1
262.1
202.1
175.1
146.1
126.1
102.1
75.1
51.1
365.2
381.1
411.2
m/z-->
NC
CN
N
N
NO2
M. W.: 325.28

MS of 6b

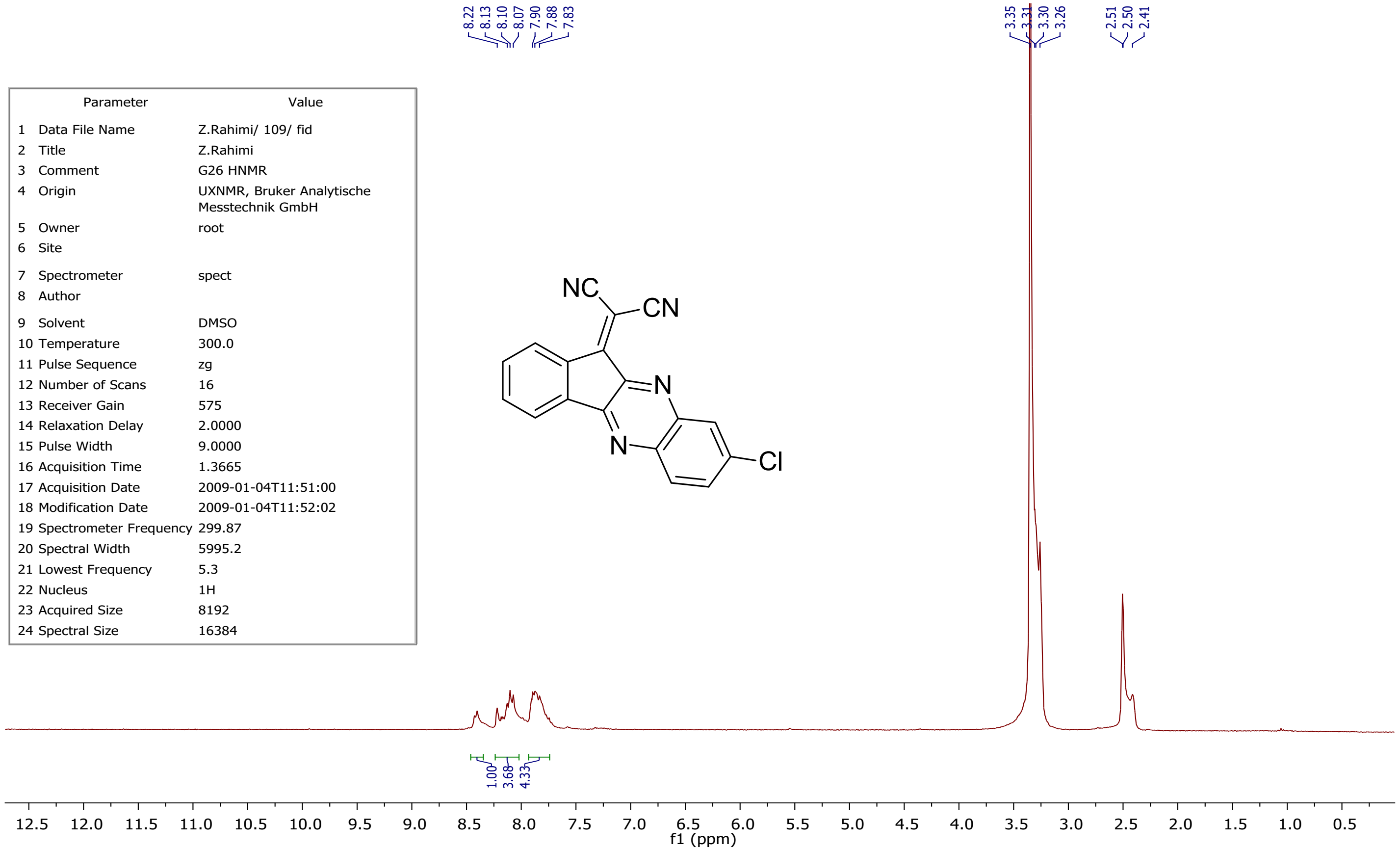

	Parameter	Value
1	Data File Name	Z.Rahimi/ 109/ fid
2	Title	Z.Rahimi
3	Comment	G26 HNMR
4	Origin	UXNMR, Bruker Analytische Messtechnik GmbH
5	Owner	root
6	Site	
7	Spectrometer	spect
8	Author	
9	Solvent	DMSO
10	Temperature	300.0
11	Pulse Sequence	zg
12	Number of Scans	16
13	Receiver Gain	575
14	Relaxation Delay	2.0000
15	Pulse Width	9.0000
16	Acquisition Time	1.3665
17	Acquisition Date	2009-01-04T11:51:00
18	Modification Date	2009-01-04T11:52:02
19	Spectrometer Frequency	299.87
20	Spectral Width	5995.2
21	Lowest Frequency	5.3
22	Nucleus	1H
23	Acquired Size	8192
24	Spectral Size	16384

^{1}H NMR of 6c

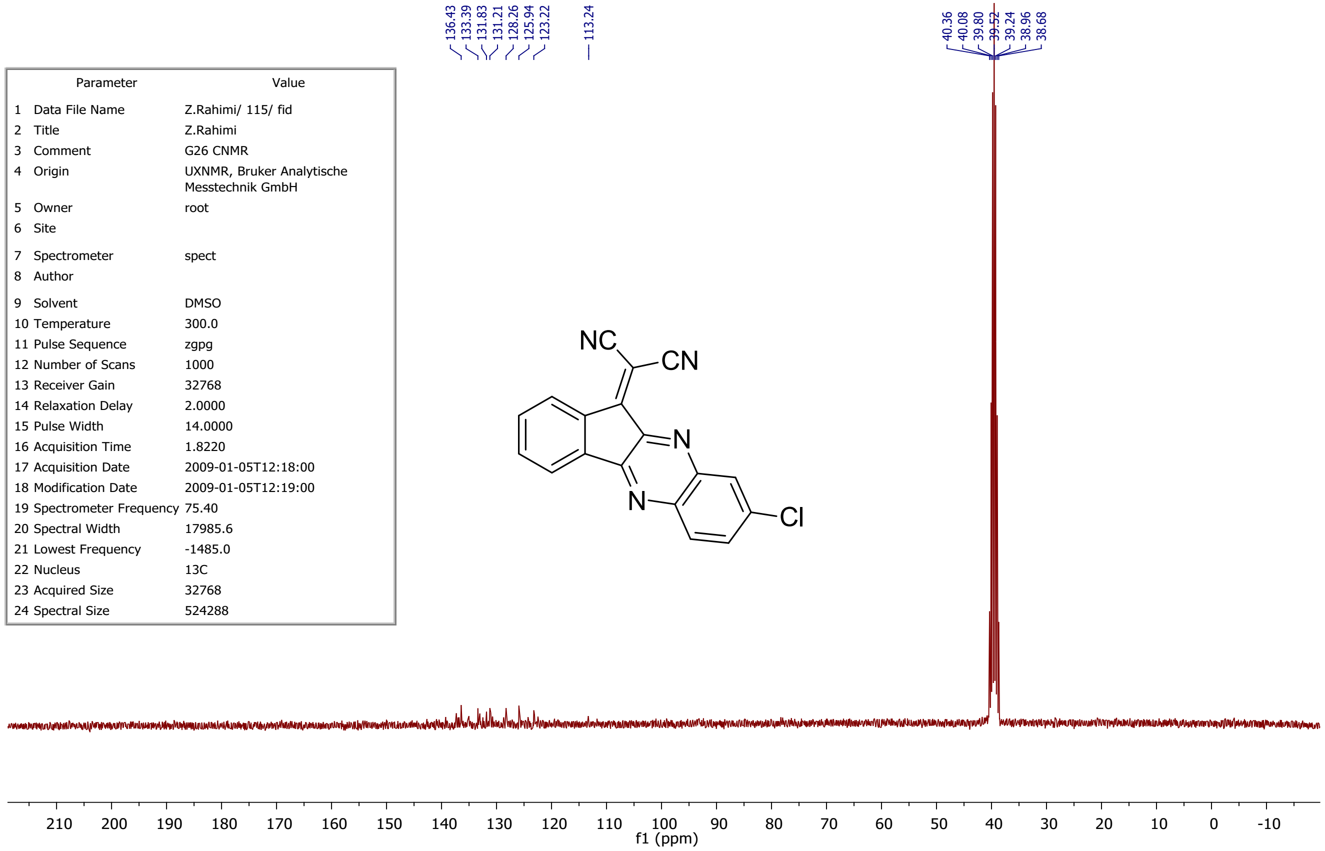

	Parameter	Value
1	Data File Name	Z.Rahimi/ 115/ fid
2	Title	Z.Rahimi
3	Comment	G26 CNMR
4	Origin	UXNMR, Bruker Analytische Messtechnik GmbH
5	Owner	root
6	Site	
7	Spectrometer	spect
8	Author	
9	Solvent	DMSO
10	Temperature	300.0
11	Pulse Sequence	zgpg
12	Number of Scans	1000
13	Receiver Gain	32768
14	Relaxation Delay	2.0000
15	Pulse Width	14.0000
16	Acquisition Time	1.8220
17	Acquisition Date	2009-01-05T12:18:00
18	Modification Date	2009-01-05T12:19:00
19	Spectrometer Frequency	75.40
20	Spectral Width	17985.6
21	Lowest Frequency	-1485.0
22	Nucleus	13C
23	Acquired Size	32768
24	Spectral Size	524288

^{13}C NMR of 6c

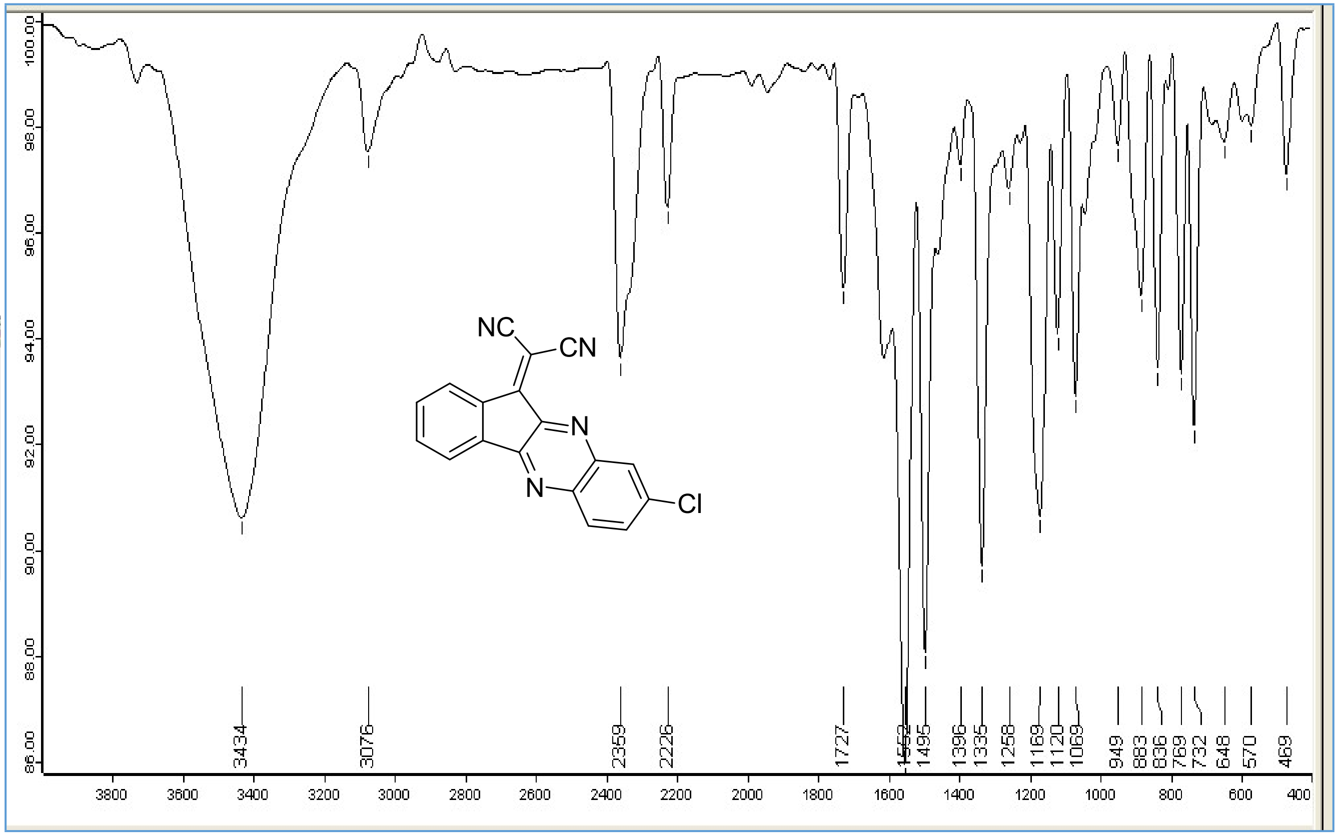

100.00
98.00
96.00
94.00
92.00
90.00
88.00
86.00
3434
3076
2359
2226
1727
1552
1495
1396
1335
1258
1169
1120
1069
949
883
836
769
732
648
570
469
3800
3600
3400
3200
3000
2800
2600
2400
2200
2000
1800
1600
1400
1200
1000
800
600
400
NC
CN
N
N
Cl

IR of 6c

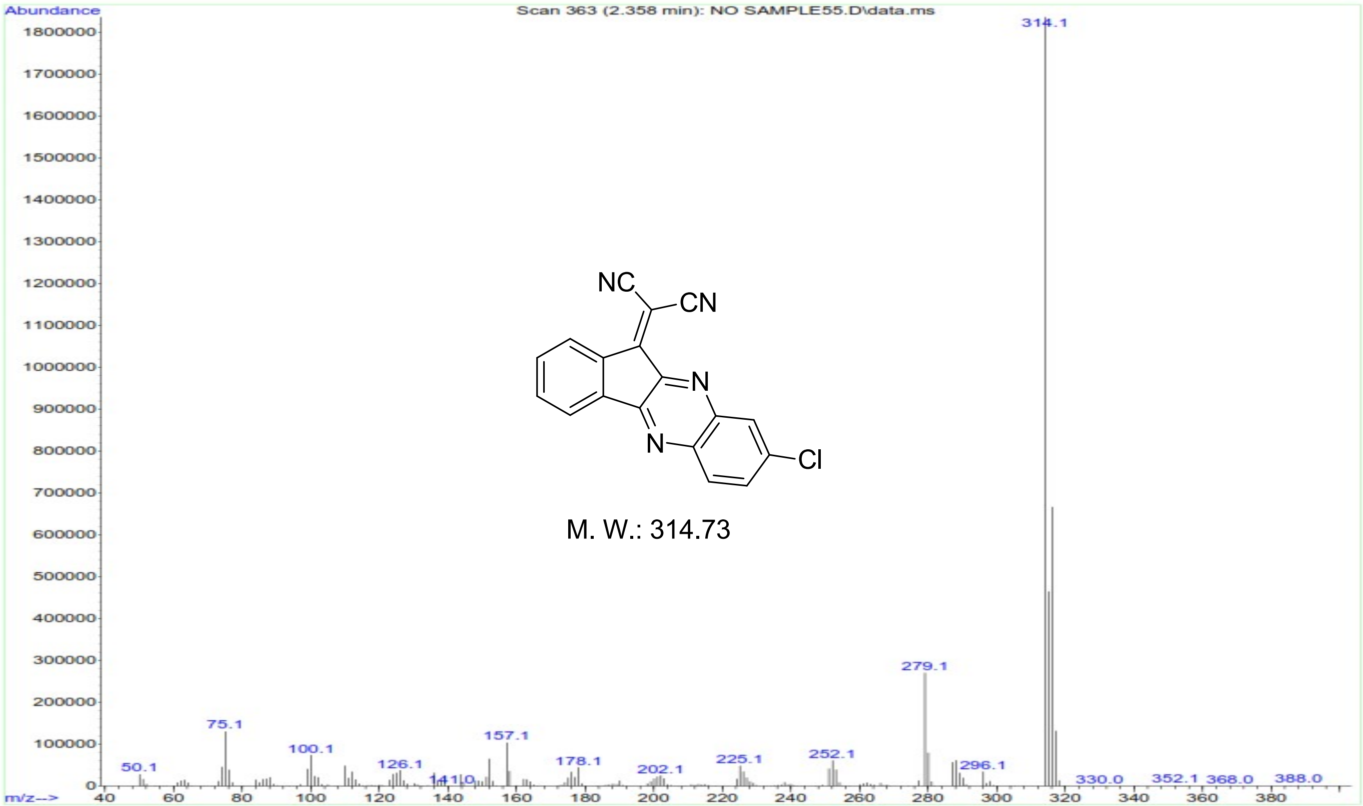
Abundance
Scan 363 (2.358 min): NO SAMPLE55.D\data.ms
314.1
279.1
75.1
100.1
157.1
50.1
126.1
141.0
178.1
202.1
225.1
252.1
296.1
330.0
352.1
368.0
388.0
m/z-->
NC
CN
N
N
Cl
M. W.: 314.73

MS of 6c

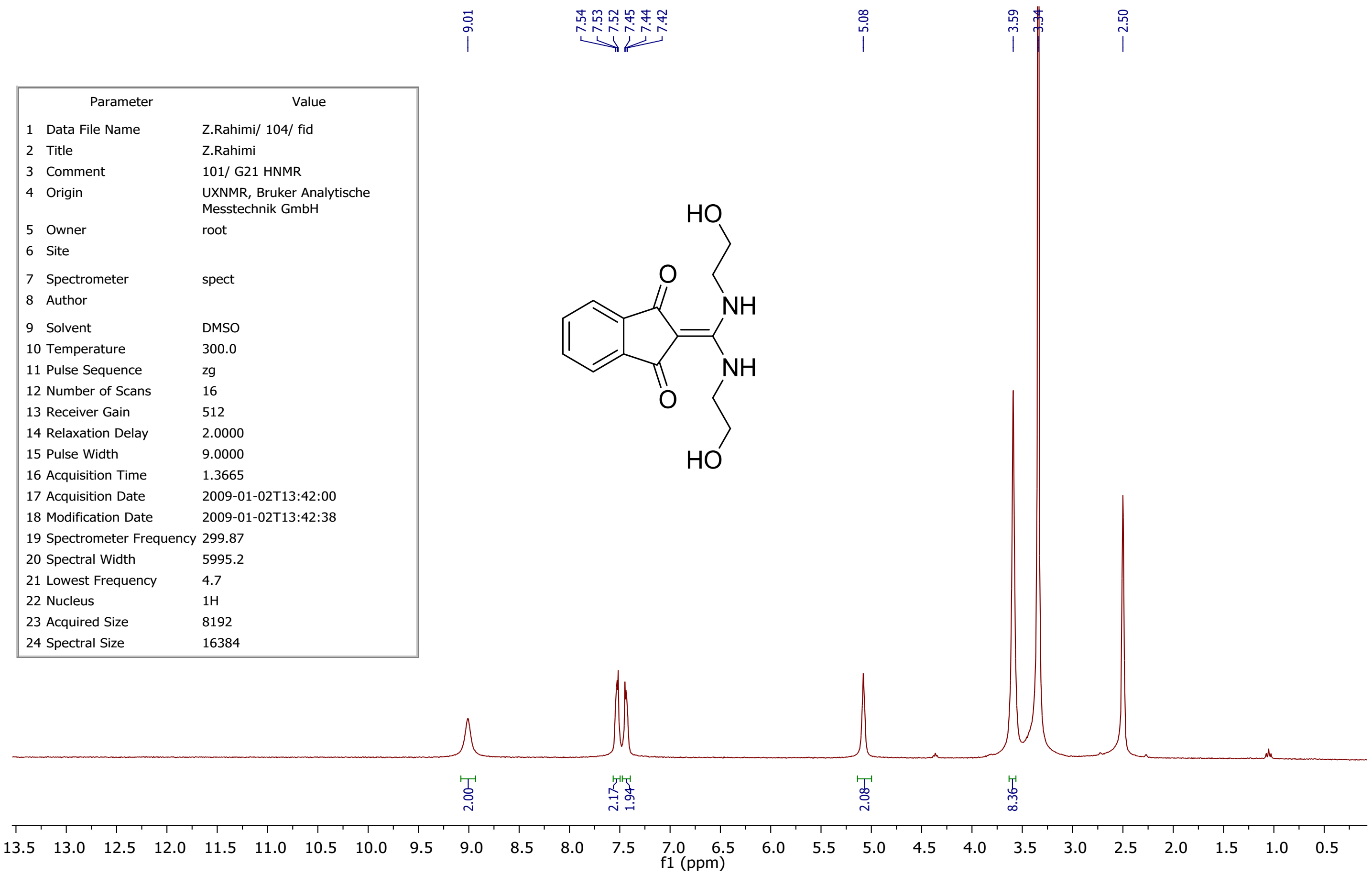

	Parameter	Value
1	Data File Name	Z.Rahimi/ 104/ fid
2	Title	Z.Rahimi
3	Comment	101/ G21 HNMR
4	Origin	UXNMR, Bruker Analytische Messtechnik GmbH
5	Owner	root
6	Site	
7	Spectrometer	spect
8	Author	
9	Solvent	DMSO
10	Temperature	300.0
11	Pulse Sequence	zg
12	Number of Scans	16
13	Receiver Gain	512
14	Relaxation Delay	2.0000
15	Pulse Width	9.0000
16	Acquisition Time	1.3665
17	Acquisition Date	2009-01-02T13:42:00
18	Modification Date	2009-01-02T13:42:38
19	Spectrometer Frequency	299.87
20	Spectral Width	5995.2
21	Lowest Frequency	4.7
22	Nucleus	1H
23	Acquired Size	8192
24	Spectral Size	16384

^{1}H NMR of 8

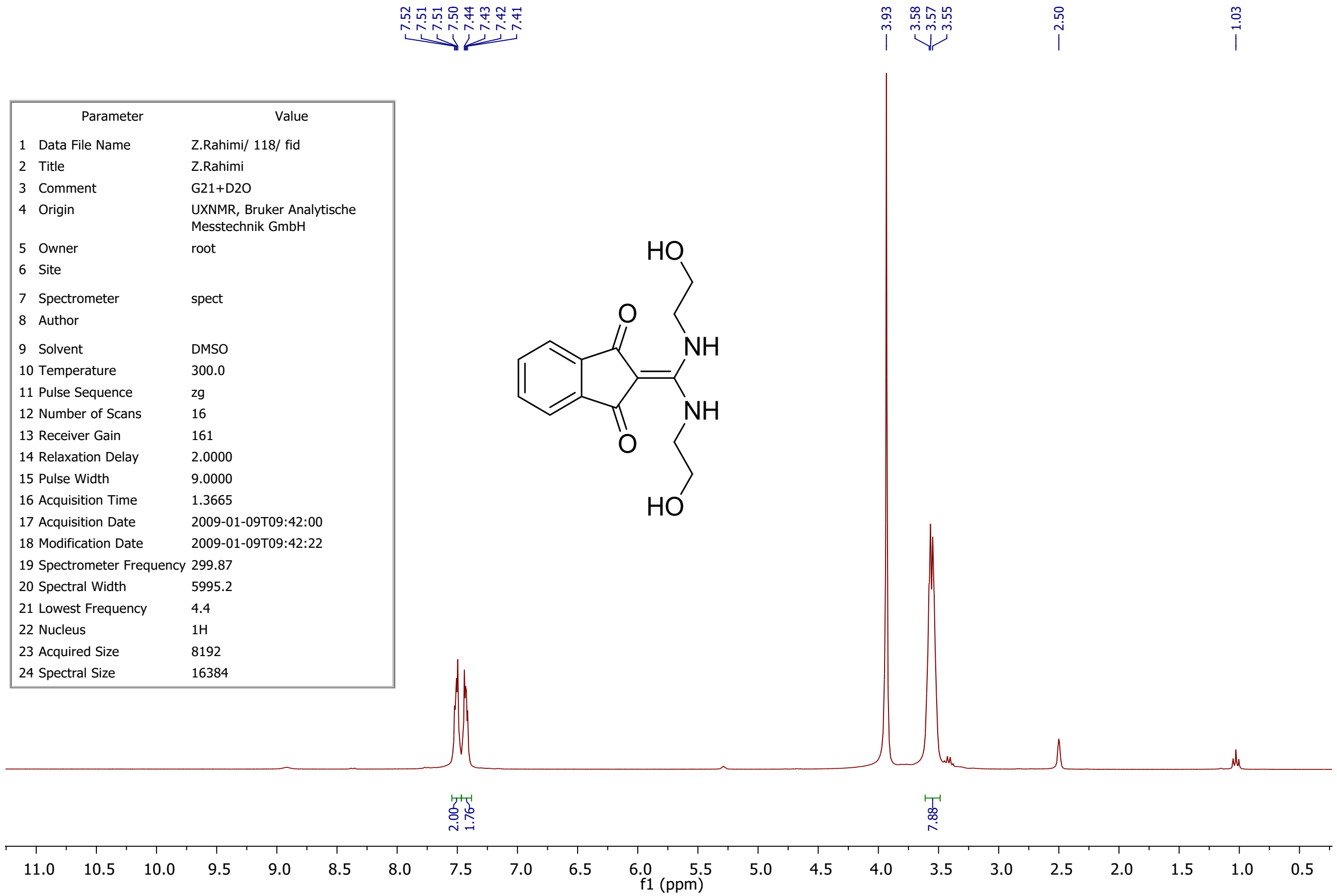

	Parameter	Value
1	Data File Name	Z.Rahimi/ 118/ fid
2	Title	Z.Rahimi
3	Comment	G21+D2O
4	Origin	UXNMR, Bruker Analytische Messtechnik GmbH
5	Owner	root
6	Site	
7	Spectrometer	spect
8	Author	
9	Solvent	DMSO
10	Temperature	300.0
11	Pulse Sequence	zg
12	Number of Scans	16
13	Receiver Gain	161
14	Relaxation Delay	2.0000
15	Pulse Width	9.0000
16	Acquisition Time	1.3665
17	Acquisition Date	2009-01-09T09:42:00
18	Modification Date	2009-01-09T09:42:22
19	Spectrometer Frequency	299.87
20	Spectral Width	5995.2
21	Lowest Frequency	4.4
22	Nucleus	1H
23	Acquired Size	8192
24	Spectral Size	16384

^{1}H-NMR with D_2O exchange of 8

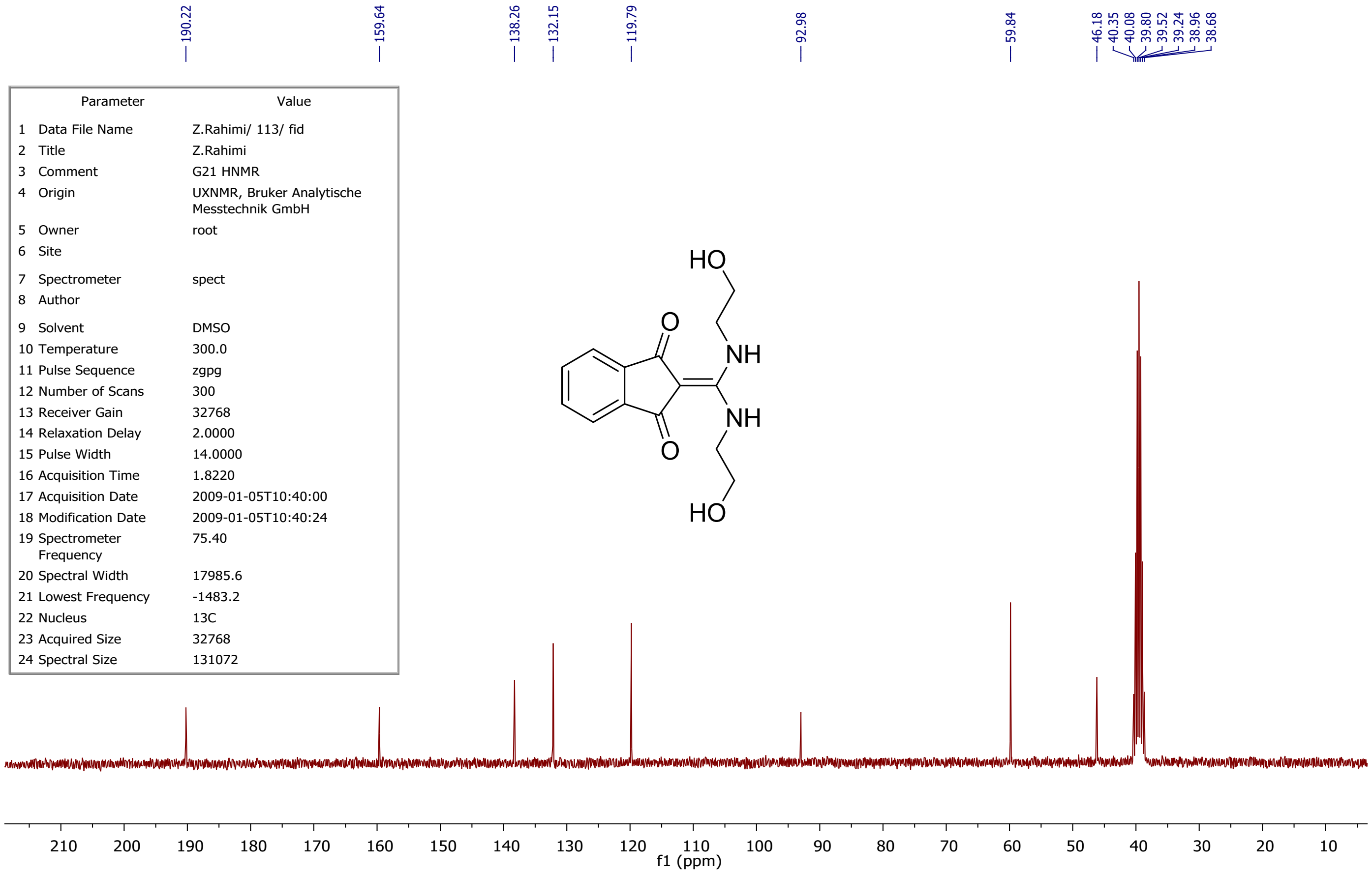

	Parameter	Value
1	Data File Name	Z.Rahimi/ 113/ fid
2	Title	Z.Rahimi
3	Comment	G21 HNMR
4	Origin	UXNMR, Bruker Analytische Messtechnik GmbH
5	Owner	root
6	Site	
7	Spectrometer	spect
8	Author	
9	Solvent	DMSO
10	Temperature	300.0
11	Pulse Sequence	zgpg
12	Number of Scans	300
13	Receiver Gain	32768
14	Relaxation Delay	2.0000
15	Pulse Width	14.0000
16	Acquisition Time	1.8220
17	Acquisition Date	2009-01-05T10:40:00
18	Modification Date	2009-01-05T10:40:24
19	Spectrometer Frequency	75.40
20	Spectral Width	17985.6
21	Lowest Frequency	-1483.2
22	Nucleus	13C
23	Acquired Size	32768
24	Spectral Size	131072

^{13}C NMR of 8

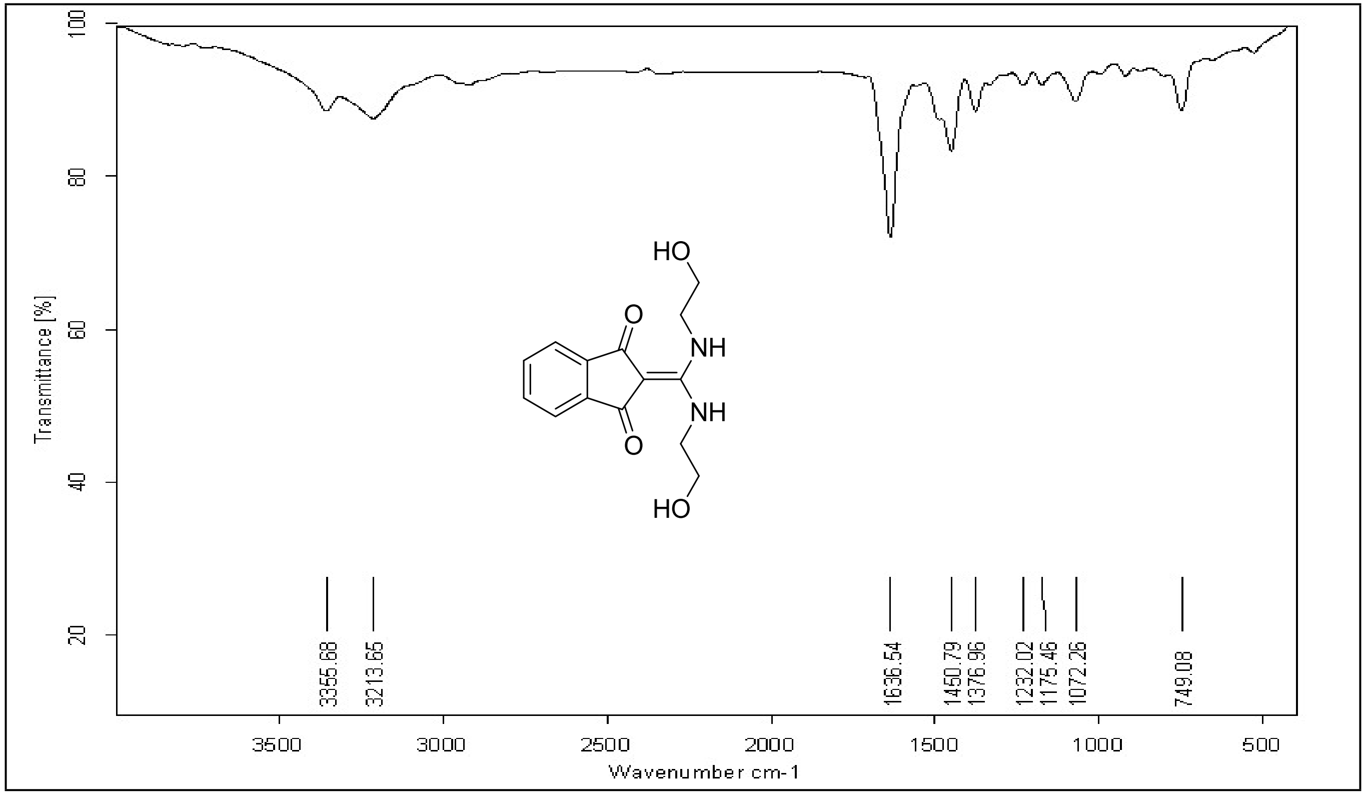
Transmittance [%]
100
80
60
40
20
3355.68
3213.65
1636.54
1450.79
1376.96
1232.02
1175.46
1072.26
749.08
3500
3000
2500
2000
1500
1000
500
Wavenumber cm-1
HO
O
NH
NH
O
HO

IR of 8

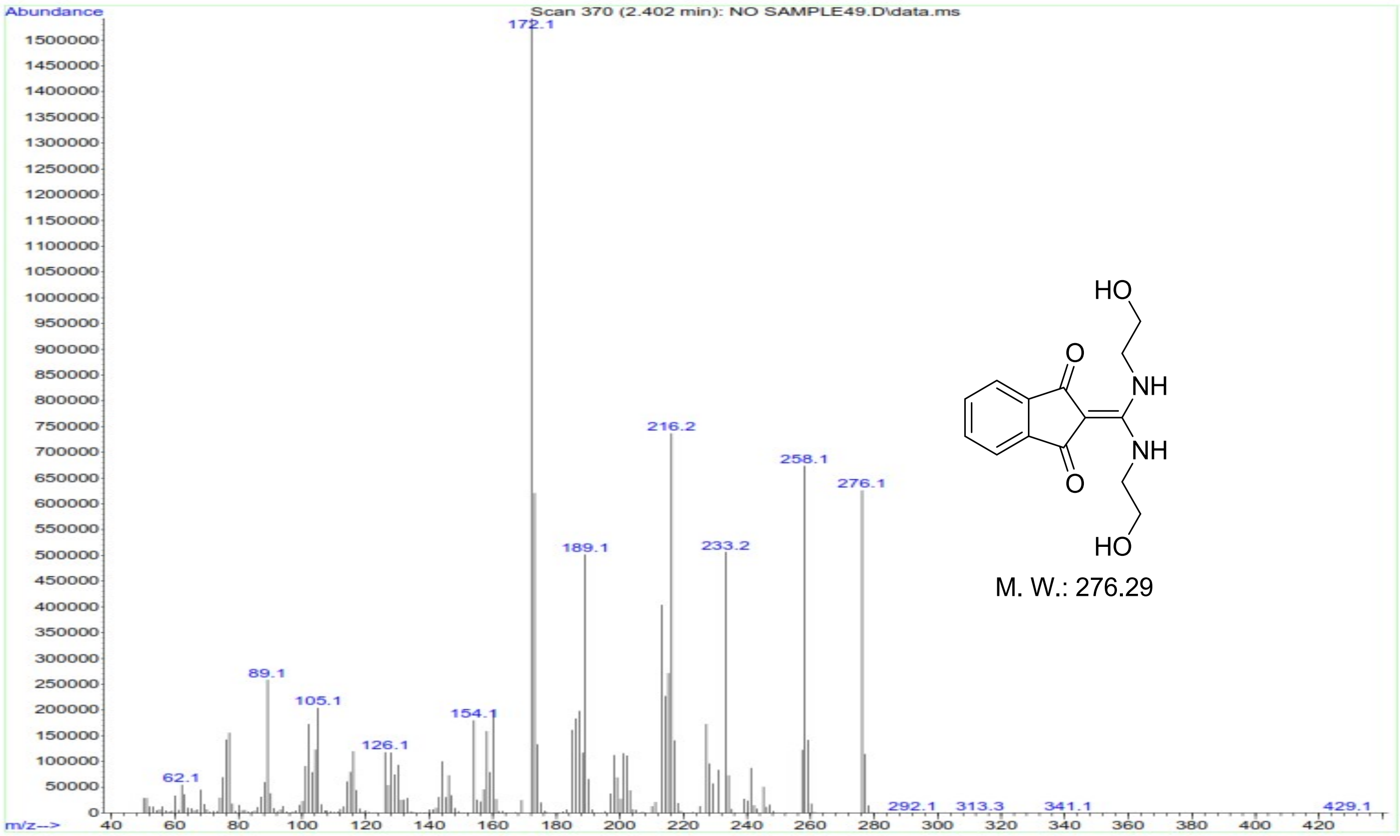
Abundance
Scan 370 (2.402 min): NO SAMPLE49.D\data.ms
172.1
216.2
258.1
276.1
189.1
233.2
89.1
105.1
126.1
154.1
62.1
292.1
313.3
341.1
429.1
m/z-->
HO
O
NH
NH
O
HO
M. W.: 276.29

MS of 8

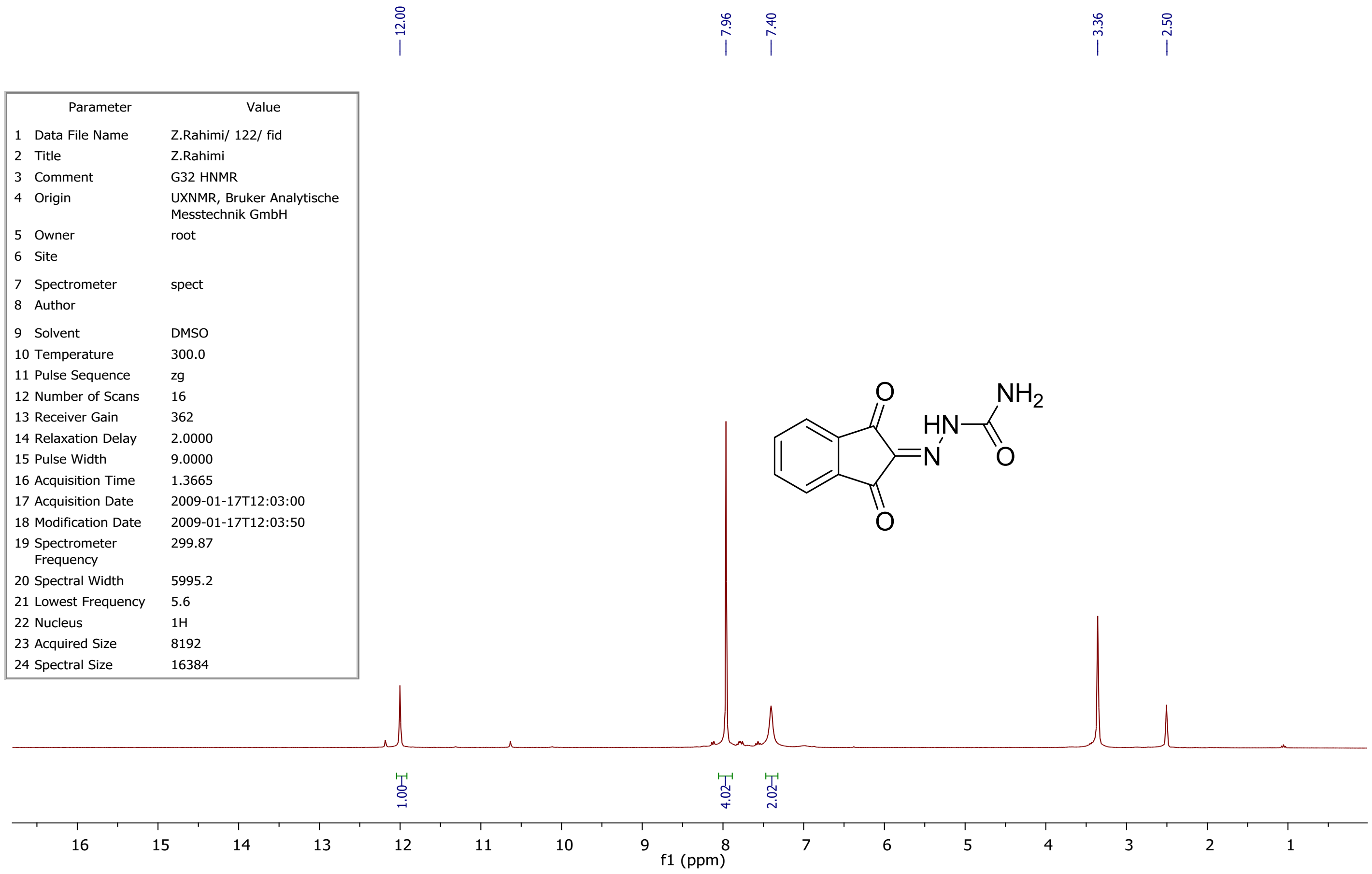

	Parameter	Value
1	Data File Name	Z.Rahimi/ 122/ fid
2	Title	Z.Rahimi
3	Comment	G32 HNMR
4	Origin	UXNMR, Bruker Analytische Messtechnik GmbH
5	Owner	root
6	Site	
7	Spectrometer	spect
8	Author	
9	Solvent	DMSO
10	Temperature	300.0
11	Pulse Sequence	zg
12	Number of Scans	16
13	Receiver Gain	362
14	Relaxation Delay	2.0000
15	Pulse Width	9.0000
16	Acquisition Time	1.3665
17	Acquisition Date	2009-01-17T12:03:00
18	Modification Date	2009-01-17T12:03:50
19	Spectrometer Frequency	299.87
20	Spectral Width	5995.2
21	Lowest Frequency	5.6
22	Nucleus	1H
23	Acquired Size	8192
24	Spectral Size	16384

^{1}H NMR of 10

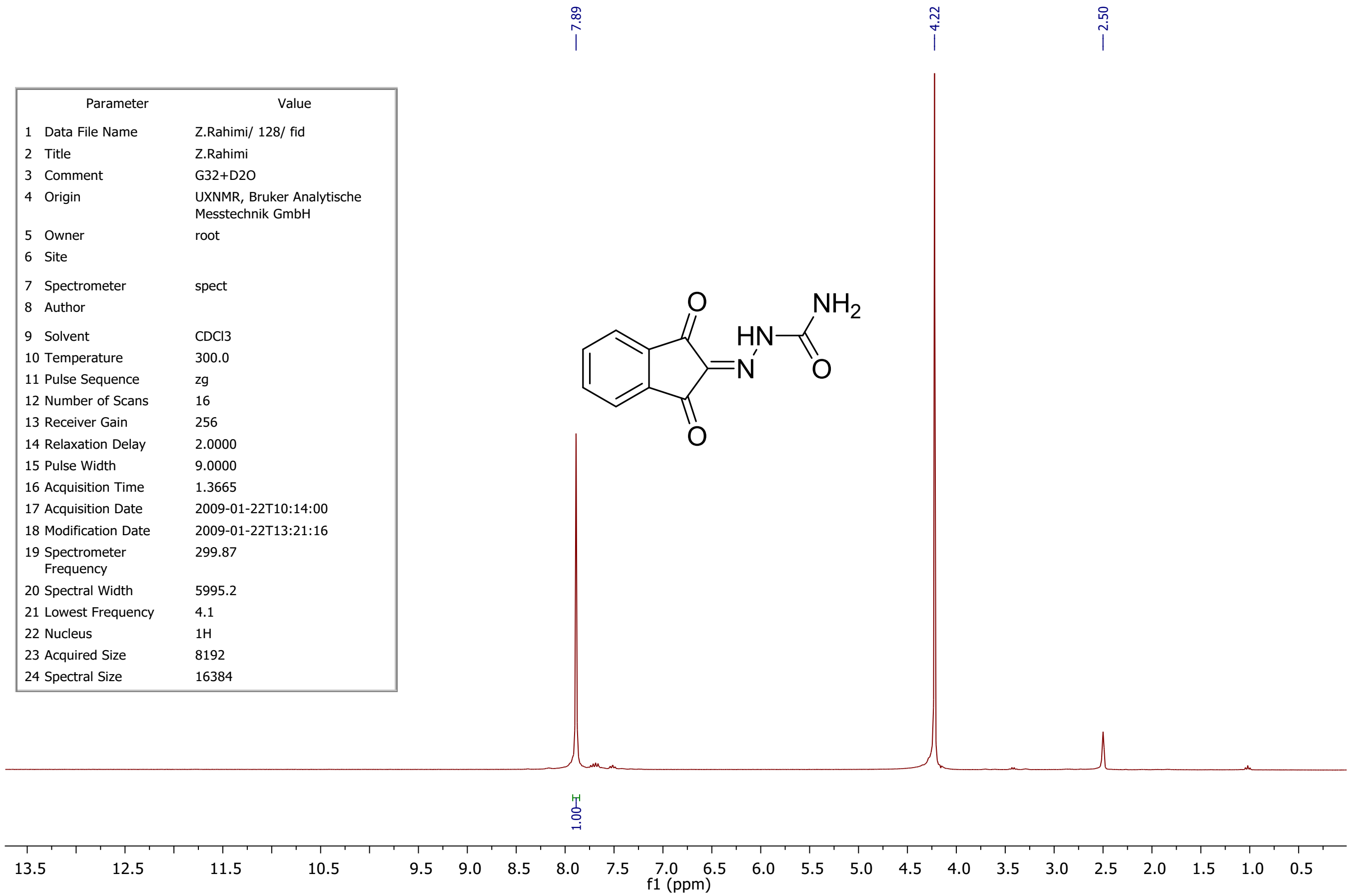

	Parameter	Value
1	Data File Name	Z.Rahimi/ 128/ fid
2	Title	Z.Rahimi
3	Comment	G32+D2O
4	Origin	UXNMR, Bruker Analytische Messtechnik GmbH
5	Owner	root
6	Site	
7	Spectrometer	spect
8	Author	
9	Solvent	CDCl3
10	Temperature	300.0
11	Pulse Sequence	zg
12	Number of Scans	16
13	Receiver Gain	256
14	Relaxation Delay	2.0000
15	Pulse Width	9.0000
16	Acquisition Time	1.3665
17	Acquisition Date	2009-01-22T10:14:00
18	Modification Date	2009-01-22T13:21:16
19	Spectrometer Frequency	299.87
20	Spectral Width	5995.2
21	Lowest Frequency	4.1
22	Nucleus	1H
23	Acquired Size	8192
24	Spectral Size	16384

^{1}H-NMR with D_2O exchange of 10

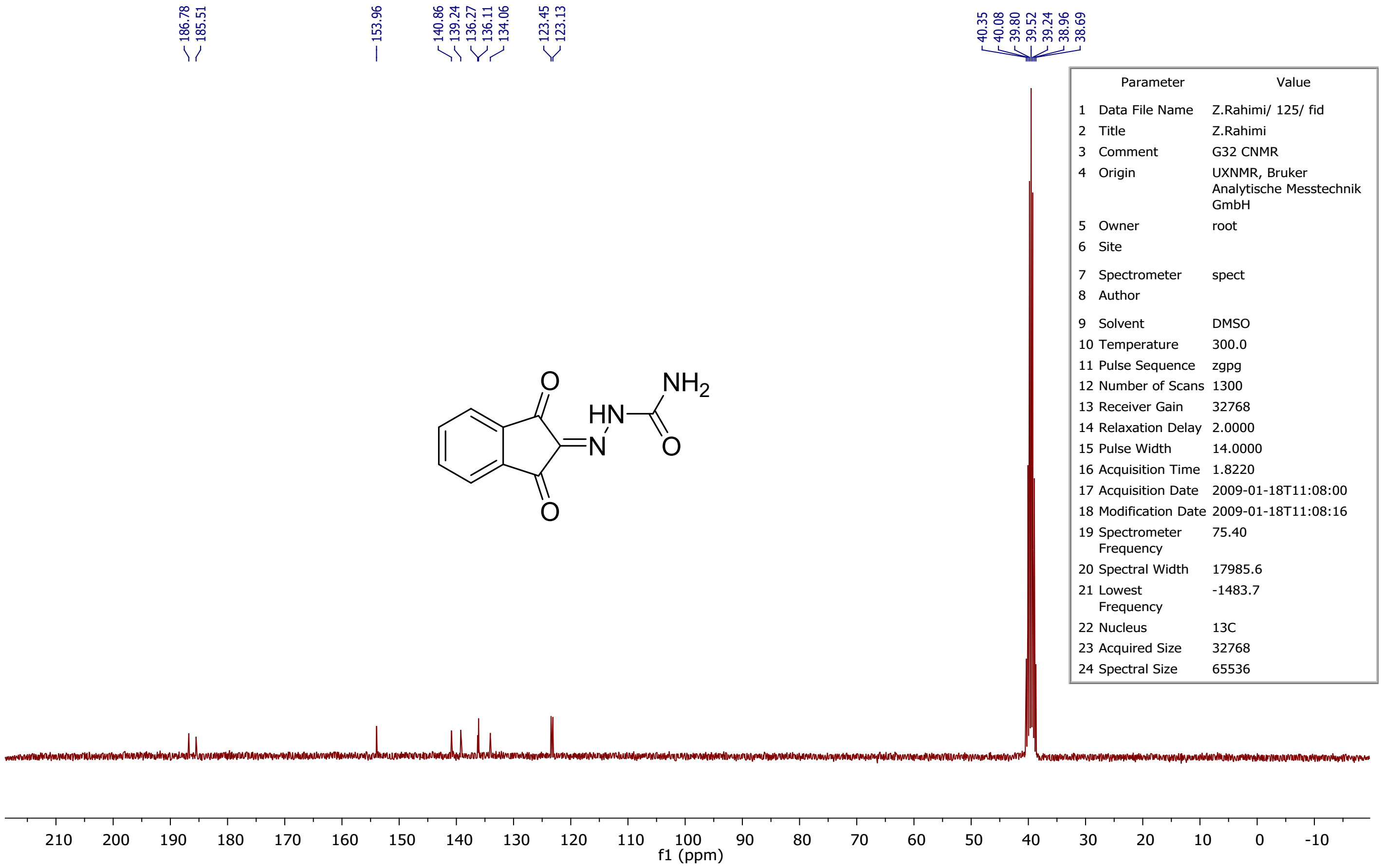
186.78
185.51
153.96
140.86
139.24
136.27
136.11
134.06
123.45
123.13
40.35
40.08
39.80
39.52
39.24
38.96
38.69
Parameter Value
1 Data File Name Z.Rahimi/ 125/ fid
2 Title Z.Rahimi
3 Comment G32 CNMR
4 Origin UXNMR, Bruker Analytische Messtechnik GmbH
5 Owner root
6 Site
7 Spectrometer spect
8 Author
9 Solvent DMSO
10 Temperature 300.0
11 Pulse Sequence zgpg
12 Number of Scans 1300
13 Receiver Gain 32768
14 Relaxation Delay 2.0000
15 Pulse Width 14.0000
16 Acquisition Time 1.8220
17 Acquisition Date 2009-01-18T11:08:00
18 Modification Date 2009-01-18T11:08:16
19 Spectrometer Frequency 75.40
20 Spectral Width 17985.6
21 Lowest Frequency -1483.7
22 Nucleus 13C
23 Acquired Size 32768
24 Spectral Size 65536
O
NH2
HN
N
O
O
210 200 190 180 170 160 150 140 130 120 110 100 90 80 70 60 50 40 30 20 10 0 -10
f1 (ppm)

^{13}C NMR of 10

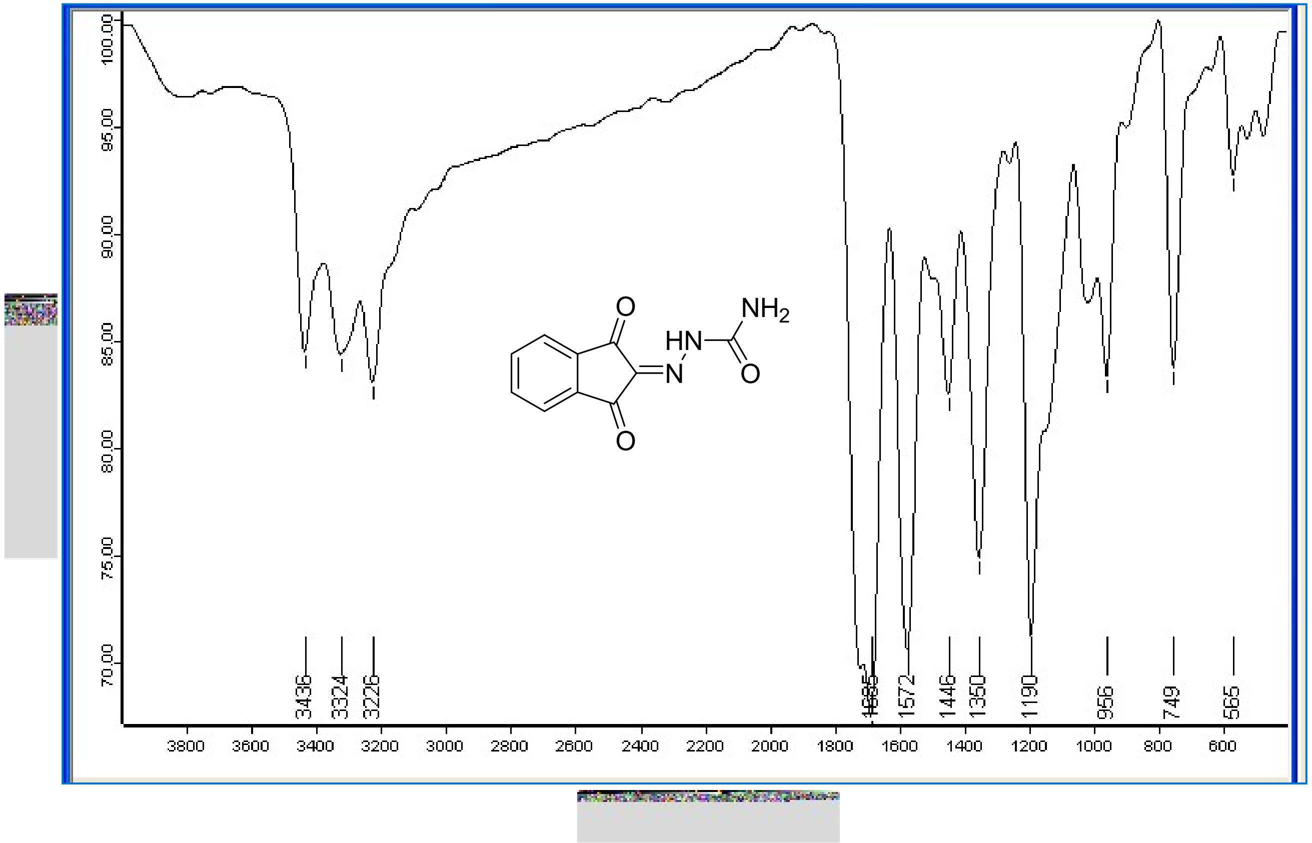
100.00
95.00
90.00
85.00
80.00
75.00
70.00
3436
3324
3226
1685
1572
1446
1350
1190
956
749
565
3800
3600
3400
3200
3000
2800
2600
2400
2200
2000
1800
1600
1400
1200
1000
800
600
O
O
NH2
HN
N
O

IR of 10

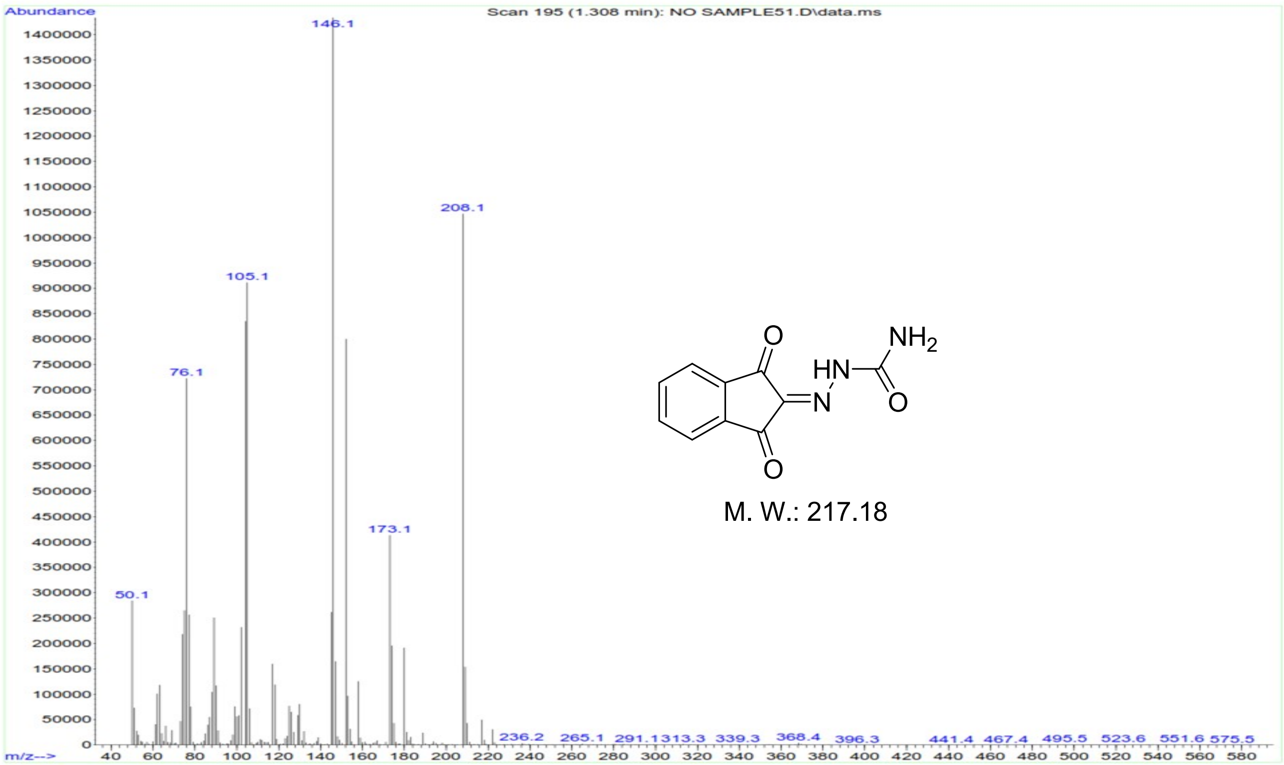
Abundance
Scan 195 (1.308 min): NO SAMPLE51.D\data.ms
146.1
208.1
105.1
76.1
173.1
50.1
m/z-->
M. W.: 217.18

MS of 10

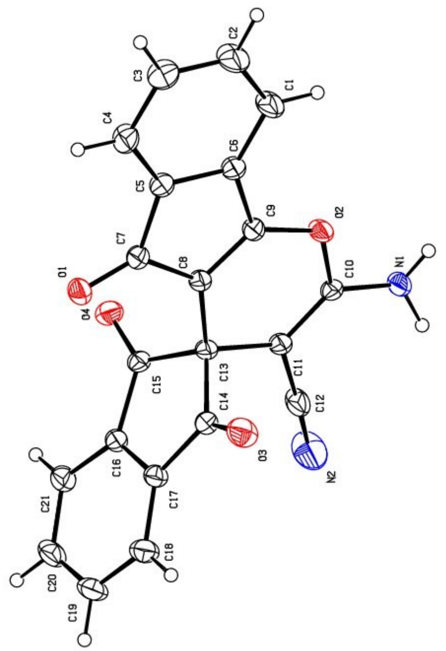

Figure 2. The X-ray structure of compound **12**.

	Parameter	Value
1	Data File Name	Z.Rahimi/ 178/ fid
2	Title	Z.Rahimi
3	Comment	G/ 77 HNMR
4	Origin	UXNMR, Bruker Analytische Messtechnik GmbH
5	Owner	root
6	Site	
7	Spectrometer	spect
8	Author	
9	Solvent	DMSO
10	Temperature	300.0
11	Pulse Sequence	zg
12	Number of Scans	16
13	Receiver Gain	228
14	Relaxation Delay	2.0000
15	Pulse Width	9.0000
16	Acquisition Time	1.3665
17	Acquisition Date	2009-06-27T09:01:00
18	Modification Date	2009-06-27T08:01:22
19	Spectrometer Frequency	299.87
20	Spectral Width	5995.2
21	Lowest Frequency	4.1
22	Nucleus	1H
23	Acquired Size	8192
24	Spectral Size	16384

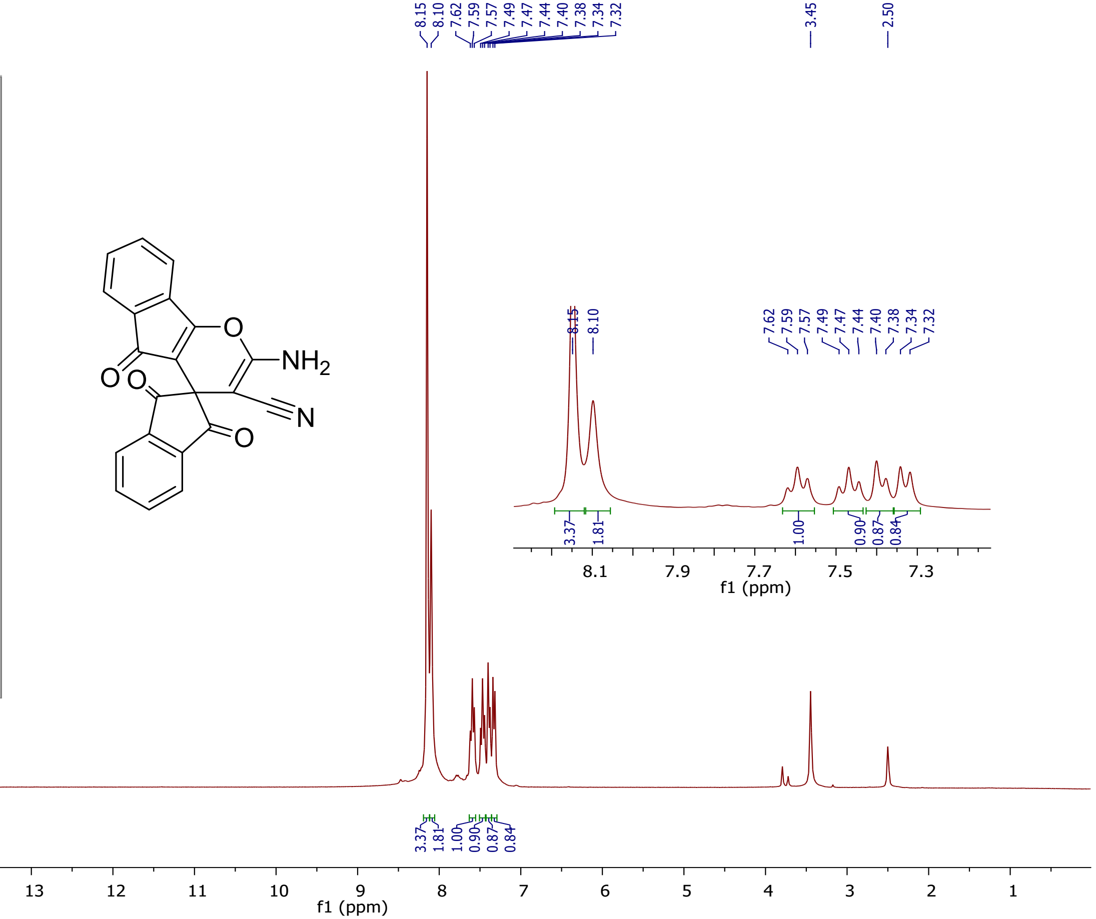

^{1}H NMR of 12

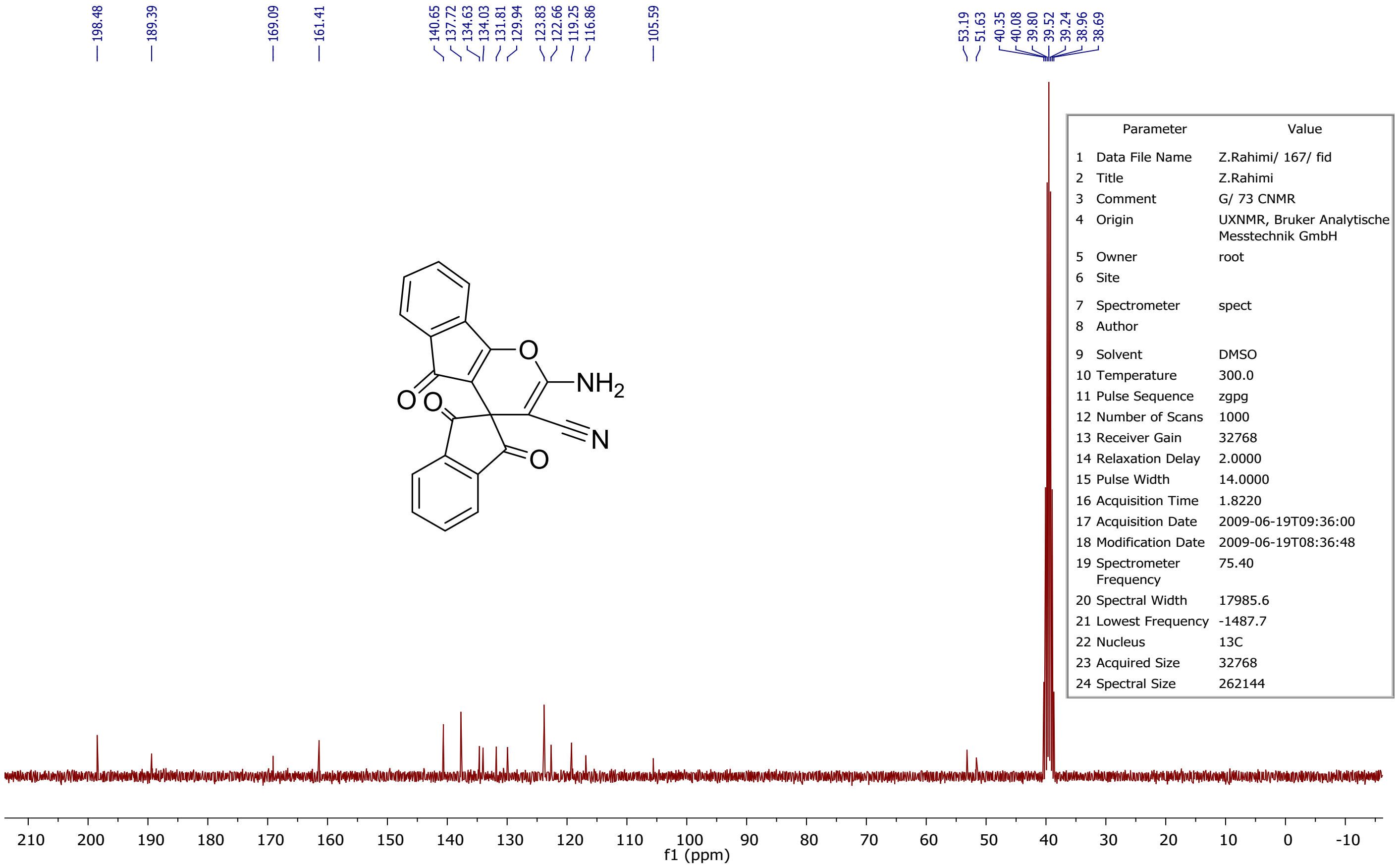

	Parameter	Value
1	Data File Name	Z.Rahimi/ 167/ fid
2	Title	Z.Rahimi
3	Comment	G/ 73 CNMR
4	Origin	UXNMR, Bruker Analytische Messtechnik GmbH
5	Owner	root
6	Site	
7	Spectrometer	spect
8	Author	
9	Solvent	DMSO
10	Temperature	300.0
11	Pulse Sequence	zgpg
12	Number of Scans	1000
13	Receiver Gain	32768
14	Relaxation Delay	2.0000
15	Pulse Width	14.0000
16	Acquisition Time	1.8220
17	Acquisition Date	2009-06-19T09:36:00
18	Modification Date	2009-06-19T08:36:48
19	Spectrometer Frequency	75.40
20	Spectral Width	17985.6
21	Lowest Frequency	-1487.7
22	Nucleus	13C
23	Acquired Size	32768
24	Spectral Size	262144

^{13}C NMR of 12

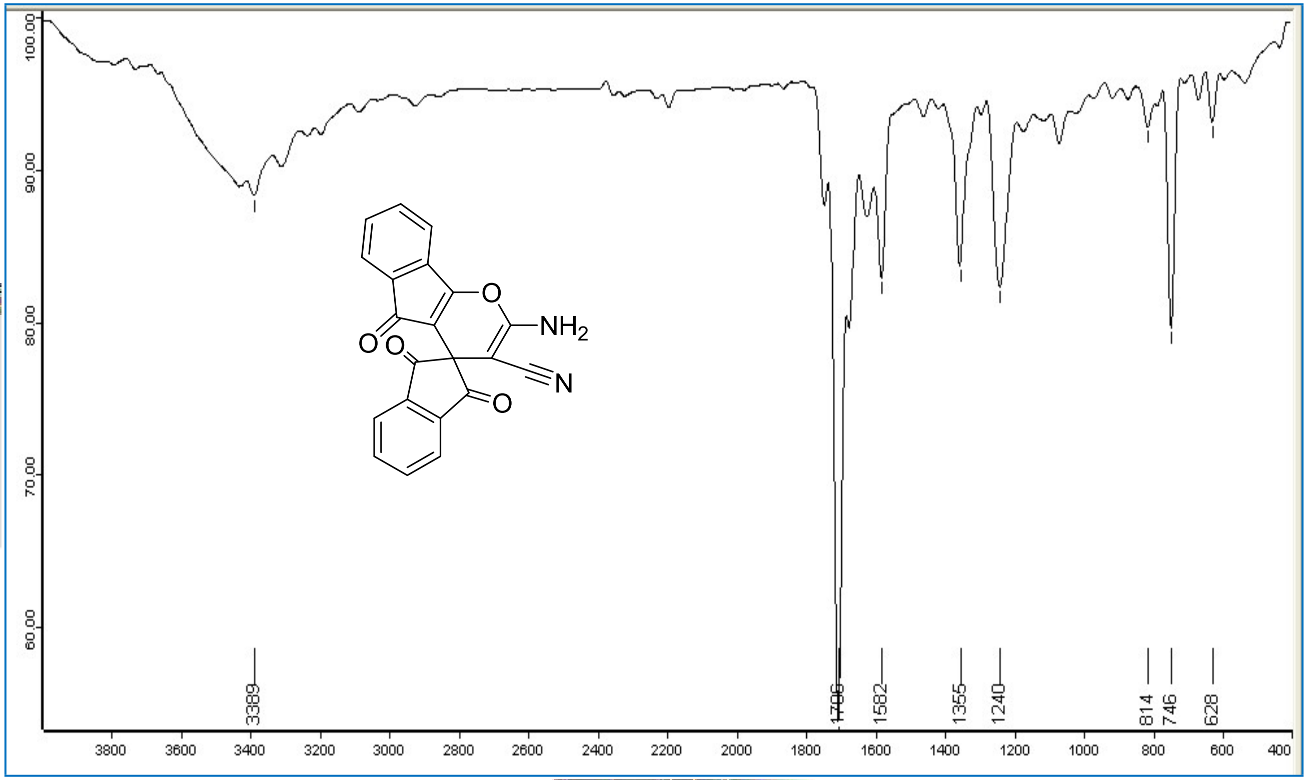
100.00
90.00
80.00
70.00
60.00
3389
1706
1582
1355
1240
814
746
628
3800
3600
3400
3200
3000
2800
2600
2400
2200
2000
1800
1600
1400
1200
1000
800
600
400
O
NH2
O
O
N
O

IR of 12

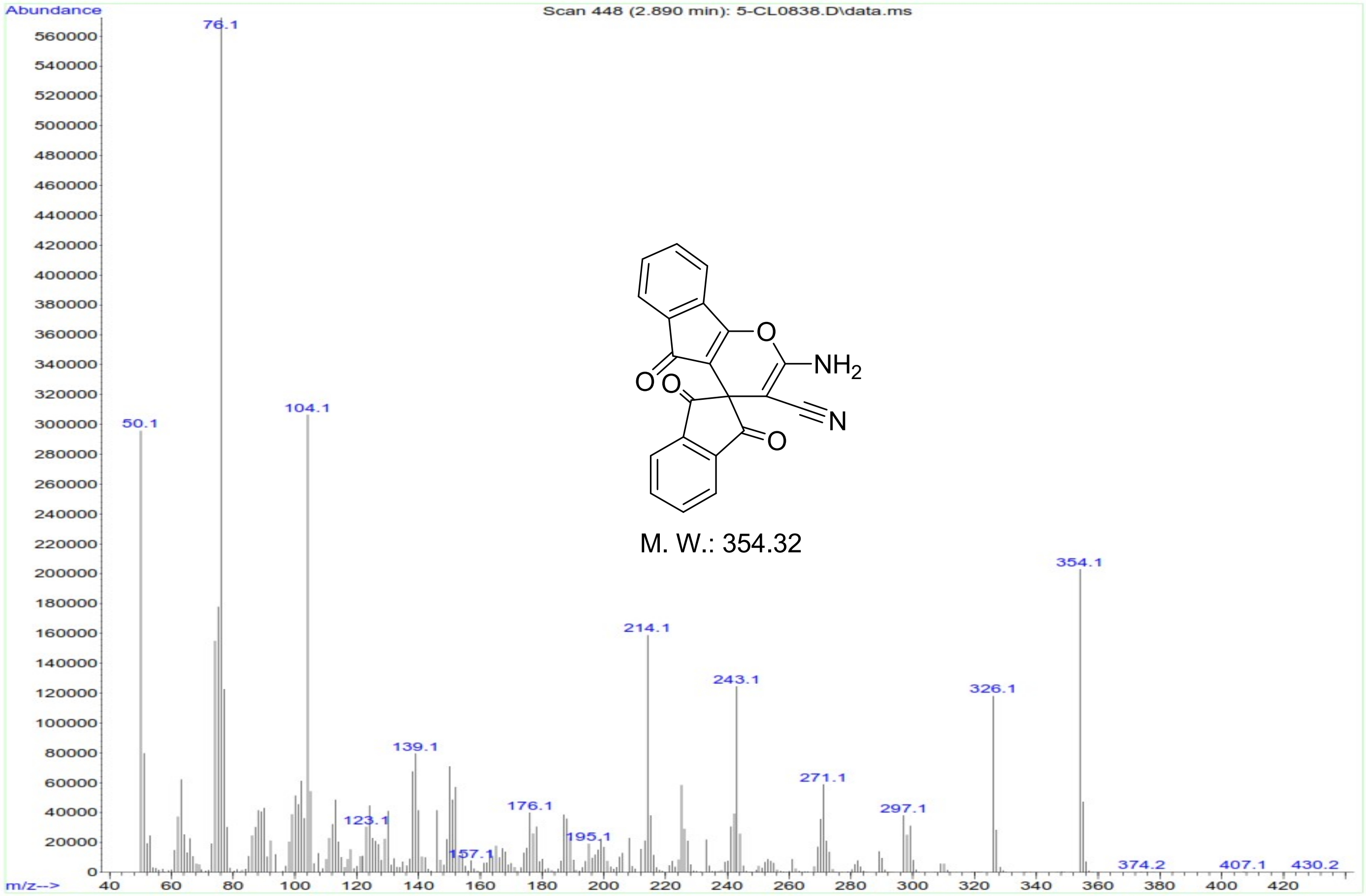

Abundance
Scan 448 (2.890 min): 5-CL0838.D\data.ms
76.1
104.1
50.1
354.1
214.1
243.1
326.1
139.1
271.1
297.1
176.1
123.1
195.1
157.1
374.2
407.1
430.2
m/z-->
O
NH2
N
O
O
O
M. W.: 354.32

MS of 12

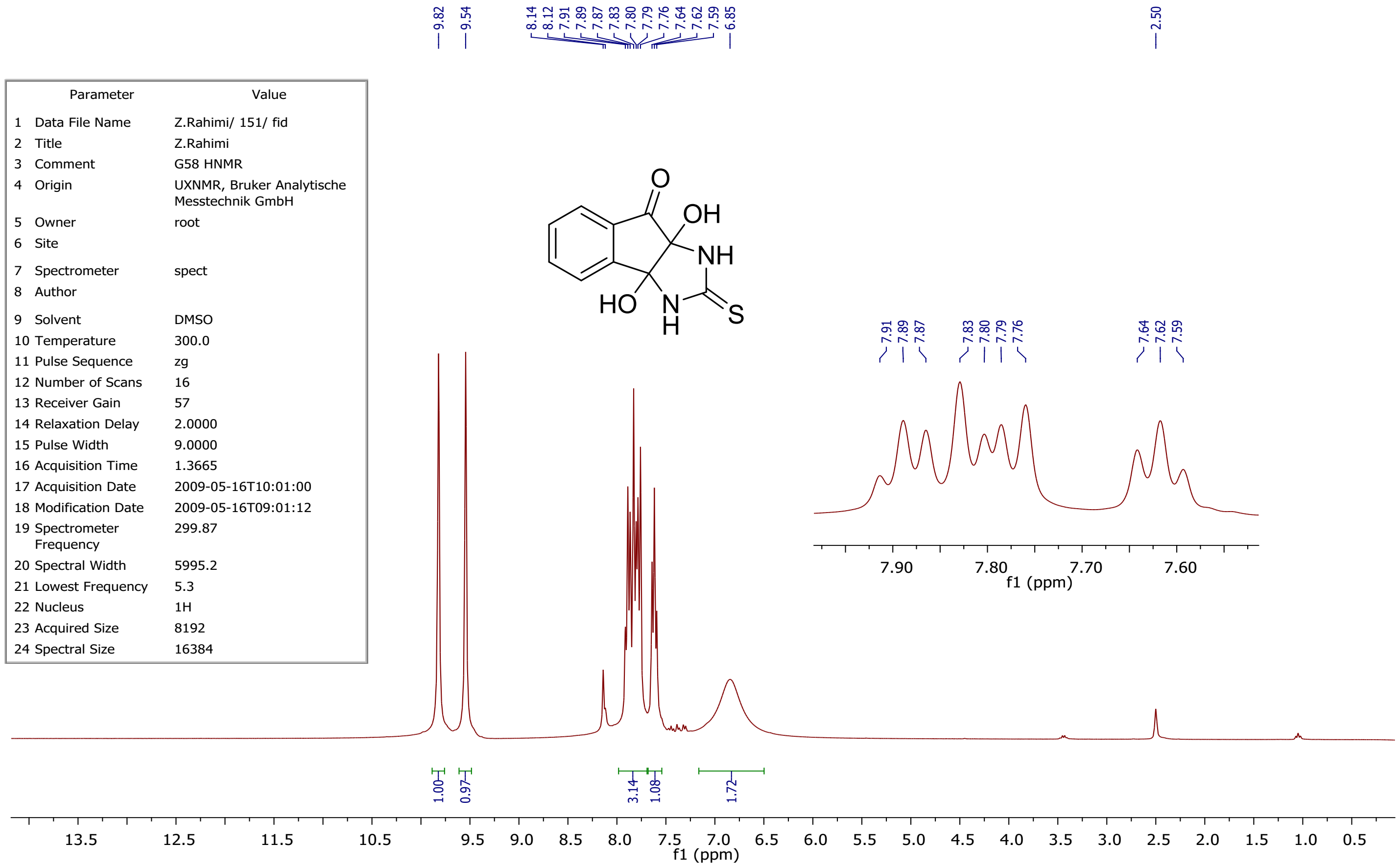

	Parameter	Value
1	Data File Name	Z.Rahimi/ 151/ fid
2	Title	Z.Rahimi
3	Comment	G58 HNMR
4	Origin	UXNMR, Bruker Analytische Messtechnik GmbH
5	Owner	root
6	Site	
7	Spectrometer	spect
8	Author	
9	Solvent	DMSO
10	Temperature	300.0
11	Pulse Sequence	zg
12	Number of Scans	16
13	Receiver Gain	57
14	Relaxation Delay	2.0000
15	Pulse Width	9.0000
16	Acquisition Time	1.3665
17	Acquisition Date	2009-05-16T10:01:00
18	Modification Date	2009-05-16T09:01:12
19	Spectrometer Frequency	299.87
20	Spectral Width	5995.2
21	Lowest Frequency	5.3
22	Nucleus	1H
23	Acquired Size	8192
24	Spectral Size	16384

^{1}H NMR of 14

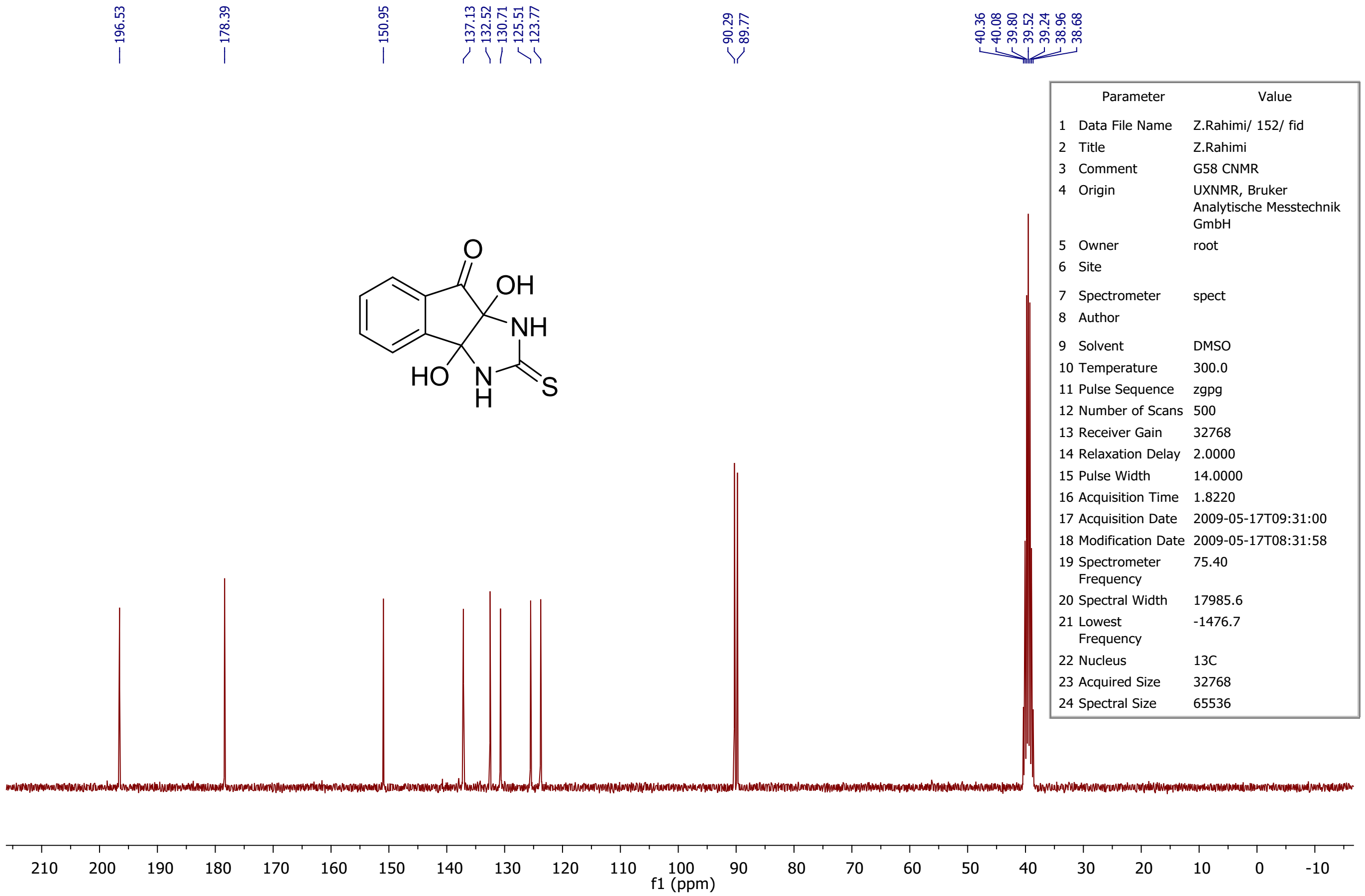
196.53
178.39
150.95
137.13
132.52
130.71
125.51
123.77
90.29
89.77
40.36
40.08
39.80
39.52
39.24
38.96
38.68
O
OH
NH
HO
N
H
S
Parameter Value
1 Data File Name Z.Rahimi/ 152/ fid
2 Title Z.Rahimi
3 Comment G58 CNMR
4 Origin UXNMR, Bruker Analytische Messtechnik GmbH
5 Owner root
6 Site
7 Spectrometer spect
8 Author
9 Solvent DMSO
10 Temperature 300.0
11 Pulse Sequence zgpg
12 Number of Scans 500
13 Receiver Gain 32768
14 Relaxation Delay 2.0000
15 Pulse Width 14.0000
16 Acquisition Time 1.8220
17 Acquisition Date 2009-05-17T09:31:00
18 Modification Date 2009-05-17T08:31:58
19 Spectrometer Frequency 75.40
20 Spectral Width 17985.6
21 Lowest Frequency -1476.7
22 Nucleus 13C
23 Acquired Size 32768
24 Spectral Size 65536
210 200 190 180 170 160 150 140 130 120 110 100 90 80 70 60 50 40 30 20 10 0 -10
f1 (ppm)

^{13}C NMR of 14

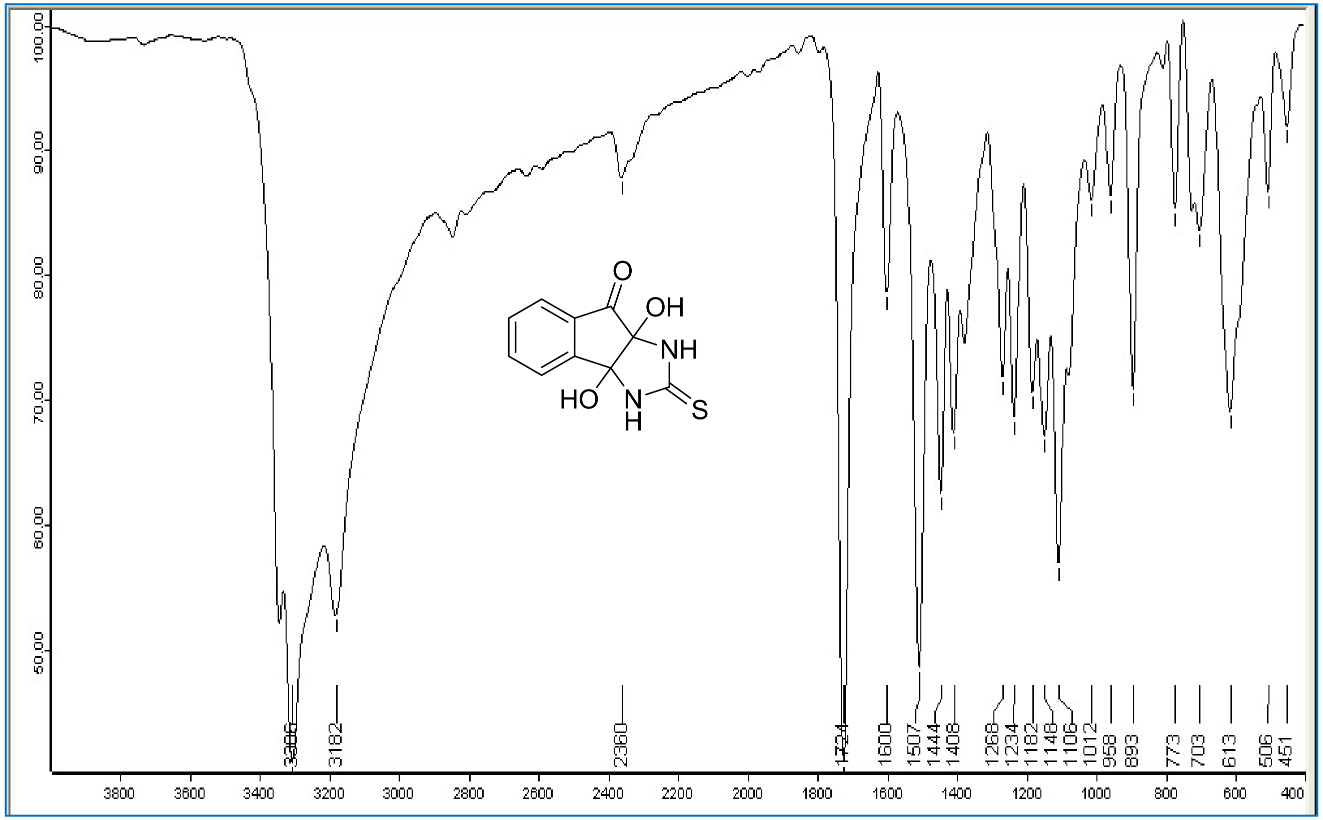
100.00
90.00
80.00
70.00
60.00
50.00
3306
3182
2360
1724
1600
1507
1444
1408
1268
1234
1182
1148
1106
1012
958
893
773
703
613
506
451
3800
3600
3400
3200
3000
2800
2600
2400
2200
2000
1800
1600
1400
1200
1000
800
600
400
O
OH
NH
HO
N
H
S

IR of 14

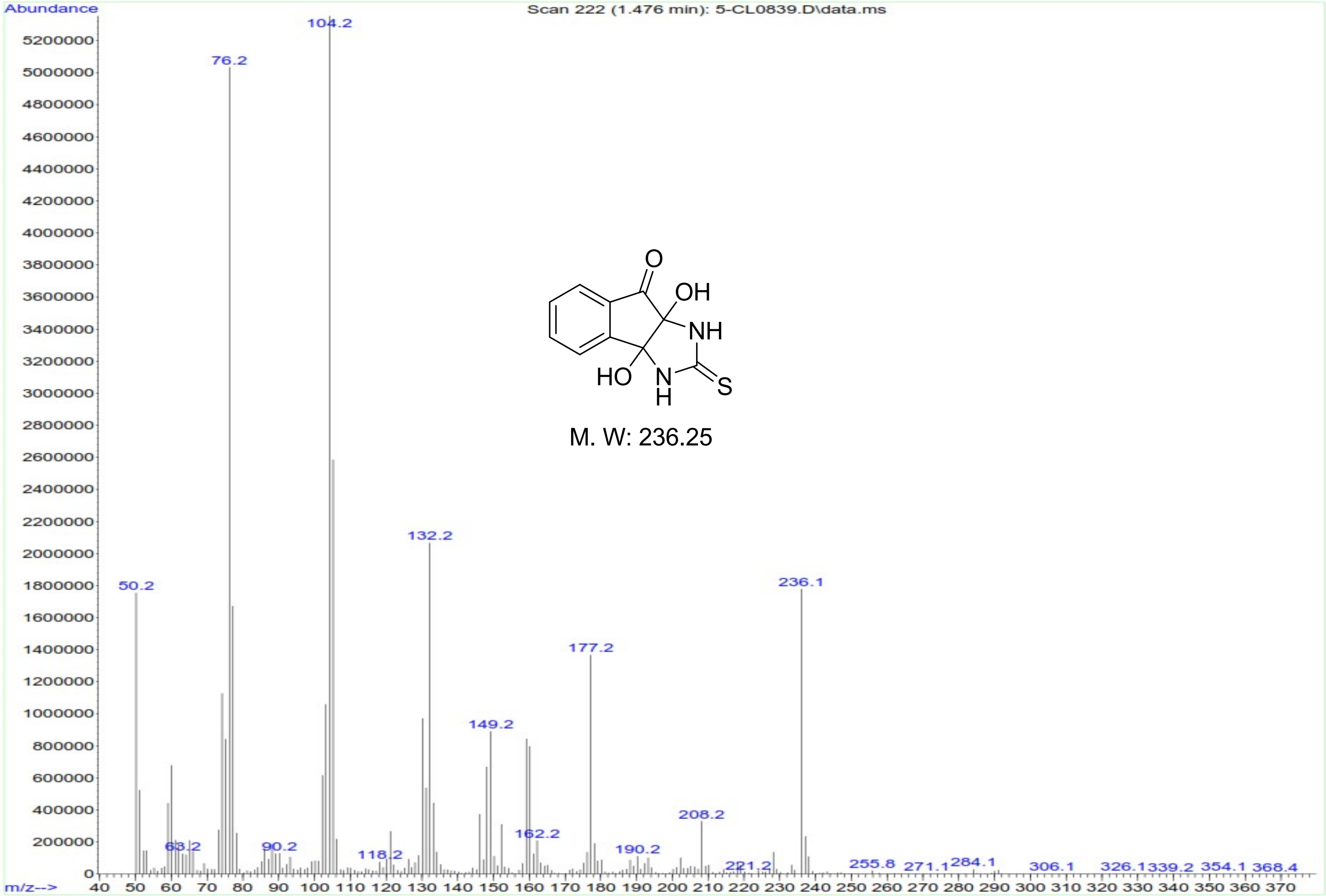

Abundance
Scan 222 (1.476 min): 5-CL0839.D\data.ms
104.2
76.2
132.2
50.2
236.1
177.2
149.2
162.2
208.2
63.2
90.2
118.2
190.2
221.2
255.8
271.1
284.1
306.1
326.1
339.2
354.1
368.4
m/z-->
O
OH
NH
HO
N
H
S
M. W: 236.25

MS of 14
